# Extrahepatic Gene Editing In Vivo Using Organic Solvent‐Free Lipid Nanoparticles

**DOI:** 10.1002/smll.202511489

**Published:** 2026-03-31

**Authors:** Michael Streiber, Na Liu, Laurianne Simon, Franziska Adermann, Vivien Bachmann, Lucas Gath, Stephanie Hoeppener, Stephanie Schubert, Oliver Werz, Vincent Lapinte, Marie Morille, Michael Bauer, Adrian T. Press, Ulrich S. Schubert, Anja Traeger

**Affiliations:** ^1^ Laboratory of Organic and Macromolecular Chemistry (IOMC) Friedrich Schiller University Jena Jena Germany; ^2^ Jena Center for Soft Matter (JCSM) Friedrich Schiller University Jena Jena Germany; ^3^ Department of Anesthesiology and Intensive Care Medicine Jena University Hospital Jena Germany; ^4^ ICGM, Univ. Montpellier, CNRS, ENSCM Montpellier France; ^5^ Department of Pharmaceutical/Medicinal Chemistry, Institute of Pharmacy Friedrich Schiller University Jena Jena Germany; ^6^ Institut Universitaire De France (IUF) Paris France; ^7^ IRMB, Univ Montpellier, INSERM Montpellier France; ^8^ Center For Sepsis Control and Care Jena University Hospital Jena Germany; ^9^ Medical Faculty Friedrich Schiller University Jena Jena Germany

**Keywords:** CRISPR‐Cas9, extrahepatic delivery, gene delivery, human primary cells, organic solvent‐free lipid nanoparticles, PEG alternative

## Abstract

Targeted therapy, which modifies genes and their expression, holds great promise for treating a variety of diseases, including cancer, inborn errors of metabolism, and acute and chronic inflammatory and infectious conditions. However, it also presents challenges related to RNA delivery, immune responses, side effects of delivery vectors, and the need for individualized formulations. To overcome these limitations, the choice of lipids and formulation processes might be re‐evaluated, with a focus on eliminating critical components, such as poly(ethylene glycol) (PEG) and ethanol. Thus, a purely water‐based formulation for lipid nanoparticles was developed, offering a material‐efficient, time‐saving process with high reproducibility. Initially, a stealth lipid containing poly(2‐methyl‐2‐oxazoline) (PMeOx) was used, and the formulation was later expanded to include approved lipids. These nanoparticles not only efficiently transfect primary human immune cells but also effectively deliver multiple nucleotides in CRISPR‐Cas9 applications. Moreover, an in vivo comparison revealed that the nanoparticles exhibited preferential transfection in extrahepatic tissues. This distinguishes them from conventional cholesterol‐rich lipid nanoparticles, which primarily target the liver regardless of the application route.

## Graphical abstract

This article highlights a novel, organic solvent‐free lipid nanoparticle (LNP) formulation that avoids cholesterol, thereby reducing hepatic accumulation and immune activation. These BLNPs enable potent, flexible, and scalable gene delivery, supporting personalized medicine and diverse nucleic acid applications. The successful use of CRISPR‐Cas9 for gene editing was demonstrated through the delivery of multiple sgRNA and Cas9 mRNA.

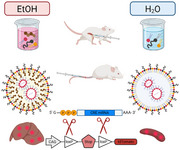

## 1 Introduction

Since the breakthrough of mRNA lipid nanoparticle (LNP) vaccines, gene transfer has been at the forefront of medical research worldwide, raising high hopes. At least 30 LNP‐based therapies are currently approved or have progressed beyond phase I clinical trials [1]. Due to the broader application of this technology, the scientific community has become aware of limitations of the LNPs, such as extrahepatic and targeted transport, stability, and immune activation [2, 3]. These limitations are also a challenge for promising applications, such as CRISPR‐Cas9 based therapies against major health challenges of modern civilization, including diabetes, cancer, and autoimmune diseases [4, 5, 6]. The availability of effective drug formulations is essential for such applications and LNPs emerged as an important vehicle for the transport of nucleic acids [7, 8, 9]. Advances are also expected to have a major medical impact in the field of congenital diseases, including inborn errors of metabolism, where gene editing, even at low levels of efficacy, is known to have broad therapeutic access [10, 11]. Even in infections, and in particular in sepsis, targeted therapy to the liver and editing the properties of immune cells to rebalance the host response represents an application with the potential to fundamentally change the understanding and treatment of these diseases [12, 13, 14].

The approval of mRNA vaccines, the ongoing clinical trials and the approval of other promising therapeutic nucleic acids or therapeutic targets illustrates the feasibility of such editing approaches [15, 16]. While lipid‐formulated mRNA, siRNA, or antisense oligonucleotides (ASOs) have been accepted by the European Medicines Agency (EMA) and the USA Food and Drug Agency (FDA), a vector‐free plasmid‐based DNA (pDNA) therapy has so far only been authorized in Japan [17]. pDNA offers advantages such as a higher stability, longer half‐life, and longtime expressions [18]. The recent approval of the world's first CRISPR‐Cas9 therapy, CASGEVY, as a cellular therapy for sickle cell disease paves the way for further therapeutic targets, and LNPs can be an important tool in this journey [19]. With the first patient‐specific CRISPR‐LNP therapy, this development reached its latest peak in May 2025 and provides a promising foundation for future advances in LNP‐based personalized medicine [20].

The current LNP drugs Patisiran (Onpattro), BNT162b (Comirnaty), and mRNA‐1273 (Spikevax) contain four different lipid types [21]. The main component of most formulations is a pH‐responsive ionizable lipid [22]. Variations in ionizable lipids affect nucleic acid delivery [23]. To increase stability and improve pharmacokinetic and pharmacodynamic properties, stealth lipids, mostly based on poly(ethylene glycol) (PEG) (PEG lipids), are added to the formulation [24]. However, the use of PEG‐lipids is being increasingly scrutinized. Due to frequent PEG contact in daily life, for example the utilization in cosmetics, the presence of anti‐PEG antibodies is widely spread in modern societies [25, 26]. The exposure to PEG‐containing pharmaceuticals, such as the PEG‐based COVID‐19 vaccines triggers this development even further [27, 28].

The reported immune response against PEG may speed up the clearance, and it can induce hypersensitivity reactions [29, 30]. Considering this dilemma, it is important to explore alternative stealth lipids. Other polymers came into the spotlight as potential PEG substitutes, e.g., zwitterionic polymers [31, 32], polysarcosines [33, 34], and poly(2‐oxazoline)s [35, 36], such as poly(2‐methyl‐2‐oxazoline) (PMeOx) [37, 38]. The use of novel polymers offers the advantage that they are not commonly present in everyday consumer products, making prior patient exposure unlikely and thereby reducing the risk of pre‐existing adverse immune responses. This is particularly relevant since anti‐PEG antibodies have shown no cross‐reactivity with other polymers, such as poly(2‐oxazoline) [39]. However, whether long‐term administration may eventually lead to antibody formation requires investigation in dedicated long‐term studies. In general, stealth lipids typically bear different types of lipids anchors, like saturated and unsaturated alkyl chains, as end groups of a stealth polymer [40, 41]. One example is lipopolyoxazolines, such as PMeOx‐lipids, exhibiting comparable tissue distribution and blood circulation time as common PEG‐lipids [42]. In addition, PMeOx showed less liver accumulation, likely due to its higher hydrophilicity compared to PEG [43].

Besides ionizable and stealth lipids, LNPs contain additional helper lipids. Phospholipids, such as DSPC, improve the encapsulation and stability, while cholesterol supports membrane fusion and avoids plasma protein binding leading to a prolonged blood circulation time [44, 45, 46]. However, cholesterol has also been shown to have a critical influence on the hepatic accumulation of LNPs and influences immunological effects [2, 3]. Reducing or eliminating cholesterol in the formulation thus facilitates the easier targeting of therapeutic goals beyond the liver. The absence of cholesterol also enables alternative and innovative production methods for LNPs, as this highly hydrophobic compound makes the use of organic solvents mandatory for LNP production [47, 48, 49].

A common production technique for LNPs is based on microfluidics or jet mixing but also other mixing techniques like vortex mixing are suitable [50]. The organic lipid phase and the aqueous phase are rapidly mixed in these processes and subsequently the solvent is removed, and the formulation is adjusted to physiological conditions [51, 52]. This formulation process is time‐consuming and limited to down‐scaling, which is essential for decentralized applications of personalized medicines requiring small batches. From an industrial perspective, the use of organic solvents is associated with increasing manufacturing costs as well as safety concerns and ethanol are controversial in the Muslim community. Additionally, current guidelines in both the European Pharmacopoeia and the United States Pharmacopeia mandate the removal of ethanol before parenteral application in pharmaceutical products [52, 53]. Organic solvent‐free manufacturing is one way of overcoming these drawbacks.

In this work, a rapid, reproducible, and robust method for the preparation of water‐based LNPs, therefore described as “blue” lipid nanoparticles (BLNP), was developed, which effectively overcomes the existing limitations. The absence of pure cholesterol was initially compensated for by an alternative stealth polymer. PMeOx, linked to a cholesterol hemisuccinate (CHEMS) group, was used as a cholesterol‐containing stealth lipid while providing water solubility. This enabled an aqueous LNP formulation without losing the beneficial properties of cholesterol within only three‐lipid component LNP, whose physicochemical properties were characterized in detail. The BLNPs revealed high efficiency and biocompatibility in HEK293T cells as well as in primary human leukocytes and were able to deliver mRNA as well as pDNA. The successful co‐delivery of Cas9 mRNA and sgRNA, enabling CRISPR‐Cas9, was ultimately demonstrated with a knockout in 94% of the targeted cells with a single treatment only. Finally, the concept of a cholesterol‐free and aqueous “blue” formulation with FDA‐approved components (SM‐102, DSPC, DMG‐PEG) was explored, showing comparable efficiencies in vitro and successful gene delivery in vivo. Thus, we have developed an innovative type of LNP (BLNP) that can be produced without organic solvents or cholesterol, making it particularly relevant for small‐scale, application‐based production, such as individualized CRISPR‐Cas9 therapy.

## 2 Results and Discussion

### 2.1 Blue Lipid Nanoparticle Formulation (BLNP)

**FIGURE 1:**
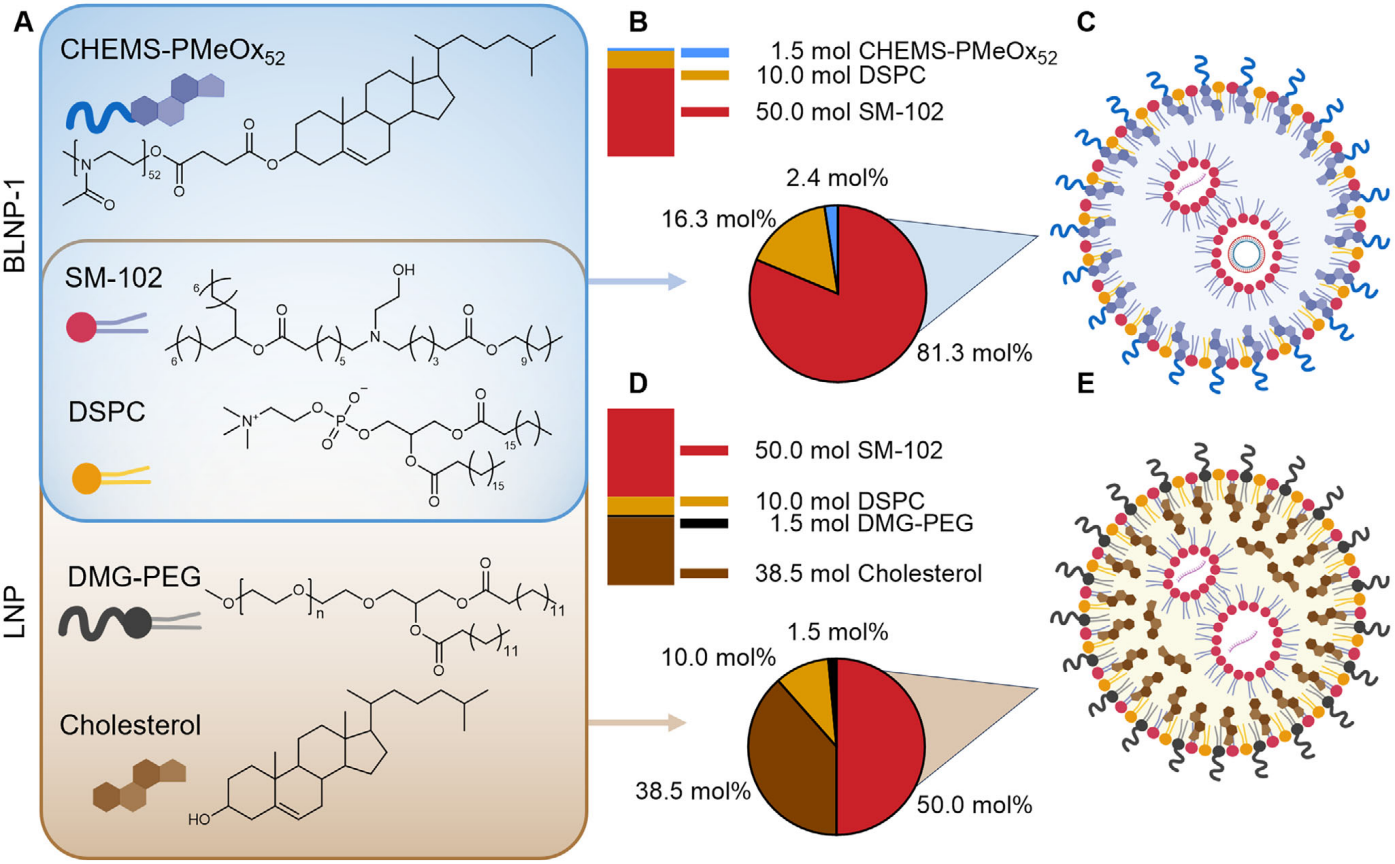
Comparison of LNP mRNA‐1273 and BLNP‐1. (A) Overview of lipids used for BLNP‐1 and LNP formulation. Blue bordered BLNP‐1, brown bordered LNP. (B) Normalized composition (50 mol SM‐102) and molar lipid ratio (mol%) of BLNP‐1. The illustration demonstrates that although BLNPs exhibit increased relative molar fractions of individual components, the net molar amount required to deliver an equivalent amount of nucleic acid is identical when the same N/P ratio is applied. (C) Schematic representation of BLNP‐1. (D) Normalized composition (50 mol SM‐102) and mol% of LNP. (E) Schematic representation of LNP mRNA‐1273.

Cholesterol, an essential component of LNPs, requires the use of organic solvents for LNP production due to its hydrophobic nature, as indicated by its hydrophilic‐lipophilic balance (HLB) value of 1.6. The HLB value is a numerical scale (ranging from 0 to 20) that indicates the relative affinity of a molecule for water (hydrophilicity) or oil (lipophilicity). Lower HLB values correspond to more lipophilic compounds, while higher values indicate greater hydrophilicity. This parameter is crucial in emulsification and nanoparticle formulation, as it influences the solubility and stability of lipid‐based systems [47, 54]. To enable organic solvent free LNP production, pure cholesterol had to be removed from the formulation. To overcome this problem while retaining the positive properties of cholesterol in LNPs, an alternative lipid was used. Thus, a cholesterol‐linked poly(2‐methyl‐2‐oxazoline) (CHEMS‐PMeOx_52_), characterized by high biocompatibility and excellent water solubility, was utilized as an alternative stealth lipid [55]. The ionizable lipid and the phospholipid added to the formulation were inspired by approved LNPs, resulting in a three‐component BLNP‐1 (Figure 1). To work in an aqueous environment, materials generally need to have a certain balance between hydrophobic and hydrophilic properties. Lipids are considered to have good water dispersibility and emulsification with HLB values between 7 and 9 [47]. The phospholipid DSPC with an HLB of 7.2 exhibits such properties. The ionizable lipid SM‐102 also fulfills these conditions, especially in its protonated form, and has been dispersed in an acidic aqueous buffer. An overview of all lipids used, including a comparison with a commercial LNP formulation (mRNA‐1273) and their corresponding HLB values, is summarized in Table S1.

**FIGURE 2:**
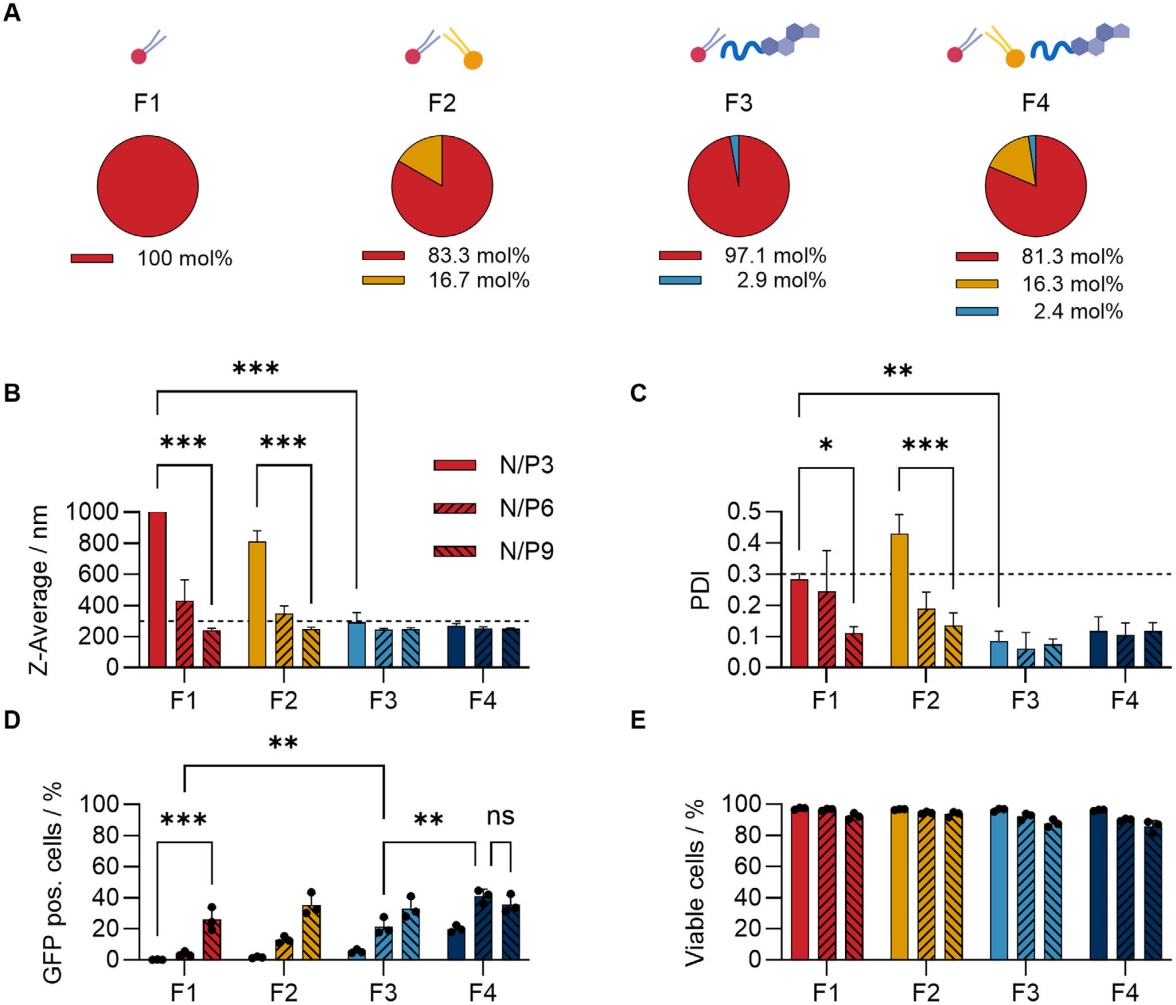
Influence of single lipids in aqueous formulations. (A) Compositions of different formulations (F1‐F4). (B) DLS measurements (Z‐Average) of F1‐F4 encapsulating pDNA (GFP). Each formulation is shown from left to right at N/P 3, 6, 9 (nitrogen to phosphate ratio). Dashed line represents 300 nm, indicating the maximal accepted range. (C) DLS measurements (PDI) of F1‐F4. Dashed line represents PDI 0.3, indicating the maximal accepted range. (D) GFP pos. cells detected via flow cytometry measurement of F1‐F4 after 24 h transfection with 3.0 µg mL^−1^ pDNA in HEK293T cells. (E) Viable cells determined via flow cytometry measurement of F1‐F4 with conditions described in (E). Data shown as mean + s.d. (B,C) *n*  =  3 replicates. (D,E) *n* = 3 biological replicates, individual data points applied). *p ≤ 0.05, **p ≤ 0.01, ***p ≤ 0.001, derived from a two‐way analysis of variance (ANOVA).

To distinguish the influence of individual lipid components in an aqueous manufacturing process, different lipid formulations (F1‐F4, Figure 2A) were tested. Starting with the ionizable lipid alone (F1), DSPC (F2), CHEMS‐PMeOx_52_ (F3), or both components together (F4) were systematically added. After the initial mixing of the lipids, the pDNA (GFP) was added by vortex mixing to achieve nucleic acid binding. A comparable simple mixing method was also established for classic LNPs before [56].

The lipid mixture was complexed with pDNA (GFP) at various ratios of nitrogen (SM‐102) to phosphate (of the genetic material), also known as the N/P ratio. DLS analysis revealed that incorporating CHEMS‐PMeOx_52_ into the formulation significantly reduced both, particle size (Z‐Average) and polydispersity index (PDI) (Figure 2B,C and Figure S1). At N/P 3, the ionizable lipid alone is not forming nanoparticles (F1, Z‐Average > 1000 nm) whereas adding CHEMS‐PMeOx_52_ (F3) enables nanoparticle formation even at N/P 3 (Z‐Average 290 nm). The size reduction is a common feature for stealth lipids, proving the impact of CHEMS‐PMeOx_52_ on potential LNP formulations [15]. The stealth properties of CHEMS‐PMeOx_52_ were further demonstrated in the work of our co‐workers, where extracellular vesicles were POxylated with this polymer. The study by Simon et al. showed a prolonged circulation time in the bloodstream [57]. In addition, the change in particle size with increasing N/P ratio was mainly due to an increased electrostatic repulsion of protonated amines at higher N/P ratios. This effect was most pronounced for F1, as opposed to N/P 3. At N/P 9 nanoparticles were formed without the addition of other lipids (240 nm).

Additionally, the transfection efficiency (amount of GFP positive cells) and viability in HEK293T cells were investigated via flow cytometry (Figure 2D,E). The addition of DSPC as well as CHEMS‐PMeOx_52_ increased transfection efficiency (F1 3%, F2 13%, F3 22% at N/P 6). It is of note that all formulated compositions exhibited remarkable biocompatibility, with no apparent signs of toxicity (Figure 2E). Revealing a synergistic effect between composition and the N/P ratio, F4 (SM‐102/DSPC/CHEMS‐PMeOx_52_/81.3/16.3/2.4) at N/P 6 emerged as the optimal composition (41% GFP pos. cells). N/P 6 was further selected because of its optimal balance between efficiency and reduced material requirements; a ratio also utilized in approved mRNA vaccines [58]. Notably, the relative molar fractions of the individual lipids are higher compared to those commonly reported for conventional LNP formulations, particularly for the ionizable lipid (≈ 50 mol% in standard LNPs compared to ≈ 80 mol% in BLNPs). Importantly, the effective mass, or absolute amount, of formulation material per delivered nucleic acid remains unchanged (Figure 1B,D), as all final formulations were prepared using an identical N/P ratio of 6. The increased molar percentages, therefore, result from the deliberate omission of cholesterol, which otherwise constitutes a substantial component of classical LNP formulations. A minimal N/P ratio is generally preferred, as increasing lipid content may enhance the relevance of potential adverse effects of the materials, such as cytotoxicity.

**FIGURE 3:**
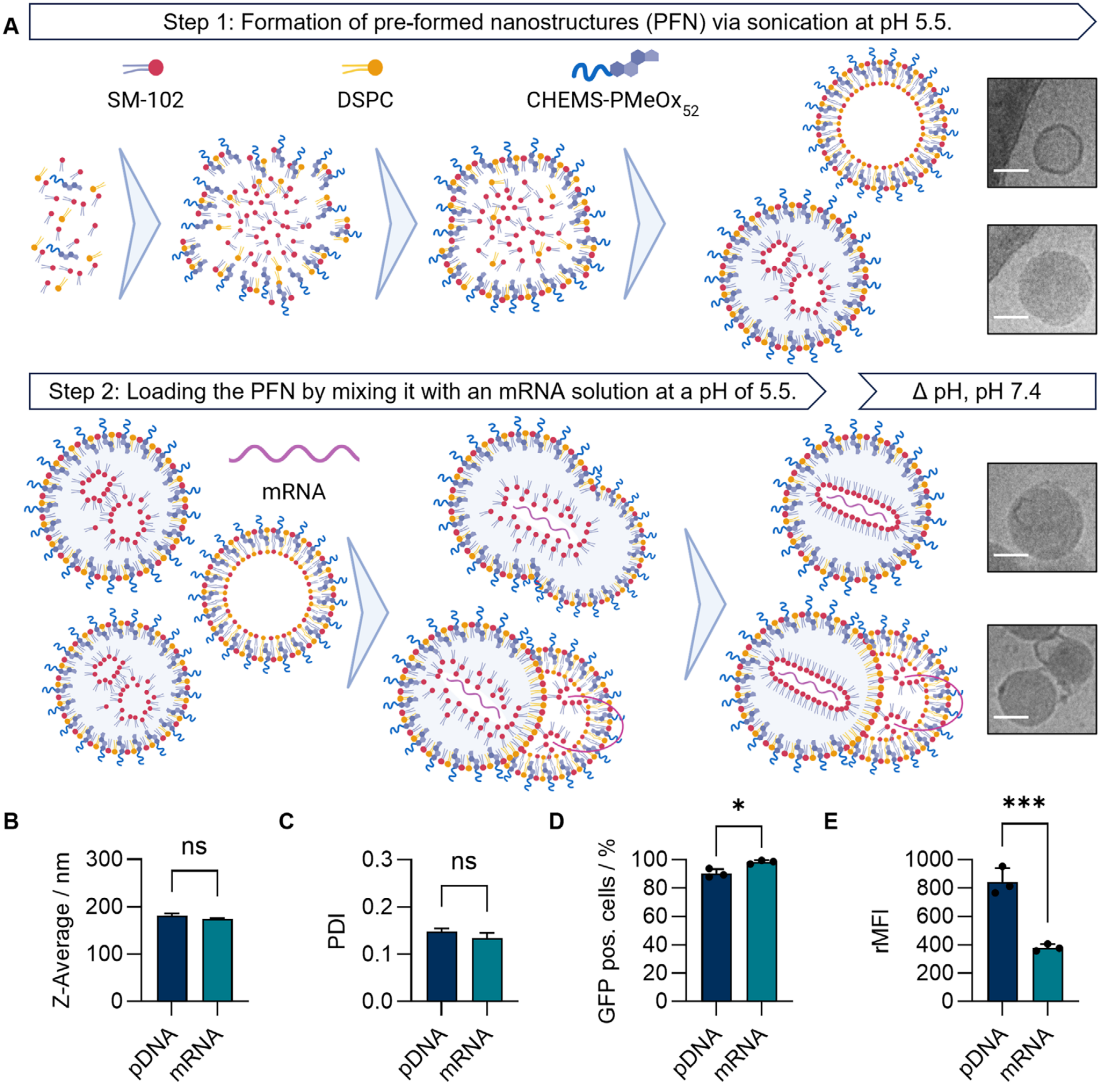
Investigations of improved BLNP‐1 formulation. (A) Proposed mechanism of BLNP formation exemplarily for mRNA. Sonication rearranges lipids in aqueous buffer (20 mM sodium acetate, pH 5.5) resulting in unloaded pre‐formed nanostructures (PFN). Mixing the PFNs with a mRNA solution at a pH of 5.5 results in nucleic acid binding and fusion, which leads to final entrapment. The pH value of the nanoparticle suspension was neutralized with PBS. The intermediate and final nanostructures are represented by cryo‐TEM observations. The scale bar is 50 nm. Additional images can be found in Figure S5. (BC) DLS measurements (Z‐Average, PDI) of BLNP‐1 loaded with mRNA (GFP) or pDNA (GFP). (D,E) Transfection investigations in HEK293T cells (GFP pos. cells, rMFI) of BLNP‐1 loaded with mRNA (GFP) or pDNA (GFP). Data shown as mean + s.d. (B,C) *n*  =  3 replicates; (D,E) 3 biological replicates, individual data points applied). *p ≤ 0.05, ***p ≤ 0.001, derived from a two‐way analysis of variance (ANOVA).

Following the initial identification of an optimal molar composition, the formulation was studied by cryo‐transmission electron microscopy (cryo‐TEM), identifying also the formation of sheets in the nanoparticle solution (Figure S2). Therefore, the formulation procedure was optimized to obtain small and monodisperse BLNPs and an improved transfection efficiency. Thus, prior to nucleic acid entrapment, the homogeneity of the aqueous lipid suspension was improved by sonication instead of simple vortex mixing. Sonication represents a common high‐energy mixing technique to ensure a homogenous lipid mixture [59]. With this procedure, the lipids rearrange and form pre‐formed nanostructures (PFN) in the absence of nucleic acids. The morphology of these nanoparticles was investigated by cryo‐TEM (Figure S3), revealing predominantly spherical assemblies of particulate and vesicular structures. Due to the absence of cholesterol, BLNPs show only a few biphasic nanoparticles which are indicated by significant differences in the electron contrast within the nanoparticles, also known as blebs [60]. The PFNs were subsequently mixed with nucleic acid (GFP) at a pH value of 5.5. Because the conditions remained acidic, the negatively charged nucleic acid attached to the positively charged PFN surface. The mixing procedure resulted in PFN fusion and lipid rearrangement, leading to the entrapment of nucleic acids. Following encapsulation, the pH value of the nanoparticle suspension was neutralized with phosphate‐buffered saline (PBS) for stabilization, resulting in the final BLNP. The proposed mechanism of BLNP formation is illustrated in Figure 3A. Nanoparticles below 200 nm (Z‐Average) (Figure 3B) were formed. The entrapment of nucleic acids in pre‐formed and loosely arranged LNPs is also described by Kulkarni et al. showing efficient siRNA entrapment in the absence of ethanol [61]. Recently, Tanaka et al. reported a comparable approach for mRNA LNPs, in which the authors highlighted the importance of the pH value during the entrapment of mRNA in pre‐formed vesicles [62]. Loaded BLNP‐1 formulated with sonication revealed smaller sizes between 170 and 190 nm in comparison to previous formulations prepared by vortex mixing (Figure 2B, F4, 250 nm). To investigate the influence of the nucleic acid admixing to PFN and the pH value change in terms of particle integrity and size, further DLS and cryo‐TEM measurements were performed and the PFN as well as particles at pH 5.5 and pH 7.4 were measured (Figures S4 and S5). It could be shown that the loading with nucleic acids does not affect the particle size, whereas the pH value change leads to higher Z‐Average values (130–180 nm). This increase in size due to the pH value change is also common and expected for standard LNPs [61]. The sizes are comparable to cholesterol‐rich LNPs prepared as reference particles (Figure S6). As indicated by cryo‐TEM observations, BLNPs exhibited comparable heterogeneity to LNPs. Similar to PFNs, the proportion of the typical bleb structure was reduced [63, 64]. In addition, the transfection efficiency using pDNA (GFP) increased from 40.9% (vortex mixing, F4) to 90.3% with BLNP‐1 (Figure 3D), comparable to the LNP reference (Figure S7).

To expand the spectrum of potential applications, the delivery of mRNA (GFP) was subsequently investigated. It was found that BLNP‐1 loaded with mRNA or pDNA revealed no significant differences in terms of size and size distribution (Figure 3B,C). Minor differences in encapsulation efficiency between the two nucleic acids were observed (Figures S8 and S9 and Table S2; pDNA: 80.3%, mRNA: 74.8%). The quantitative assay revealed a lower mRNA binding for BLNP‐1 compared to the LNP control (89.7%) (Figure S9). This may be attributed to the absence of pure cholesterol, which has been proposed to enhance nucleic acid encapsulation by increasing membrane rigidity [45]. In conventional LNPs, cholesterol typically makes up a substantial fraction, often exceeding 30 mol% [58]. Notably, despite the application of multiple washing steps to the control LNPs to remove organic solvent and free mRNA, residual free mRNA was still detected. A preliminary investigation that used a herringbone chip microfluidic mixer instead of a vortex mixer did not improve the mRNA encapsulation efficiency (Figure S10). However, the experiments demonstrated the compatibility of microfluidic processing with BLNP technology. This could be crucial for scaling‐up production and future optimization. Differences in biological performance were observed between pDNA and mRNA formulations. The number of GFP‐expressing cells showed a slight increase when using mRNA compared to pDNA, rising from 90% to 98% (Figure 3D). However, the amount of GFP, measured as relative mean fluorescence intensity (rMFI) values, was significantly lower (*p* < 0.001) compared to pDNA (Figure 3E). The use of pDNA for gene delivery holds great promise for various therapeutic areas, primarily due to its ability to enable a stable and long‐lasting expression of the gene of interest [65]. Both nucleic acids are applicable in various gene delivery scenarios and support the flexibility and robustness of the BLNP platform.

An overview of all BLNP formulations and nucleic acids used is provided in Table 1.

**TABLE 1:** Overview of BLNP formulations and nucleic acids.

Nanoparticle	Formulation method	SM‐102 mol%	DSPC mol%	Stealth lipid mol%	Nucleic acid	Abbreviation
**BLNP‐1**	Sonication	81.3	16.3	2.4 (CHEMS‐PMeOx_52_)	pDNA (GFP)	BLNP‐1
					mRNA (GFP)	
					mRNA (Cas9), sgRNA (Anti‐GFP)	BLNP‐1_Cas9_
					mRNA (tdTomato)	BLNP‐1_tdT_
					mRNA (Cy5‐GFP)	BLNP‐1_Cy5_
					mRNA (CRE)	BLNP‐1_CRE_
**BLNP‐2**	Sonication	81.3	16.3	2.4 (DMG‐PEG)	pDNA (GFP)	BLNP‐2
					mRNA (GFP)	
					mRNA (CRE)	BLNP‐2_CRE_

### 2.2 CRISPR‐Cas9 Framework—Evaluation of mRNA and pDNA Transfection

**FIGURE 4:**
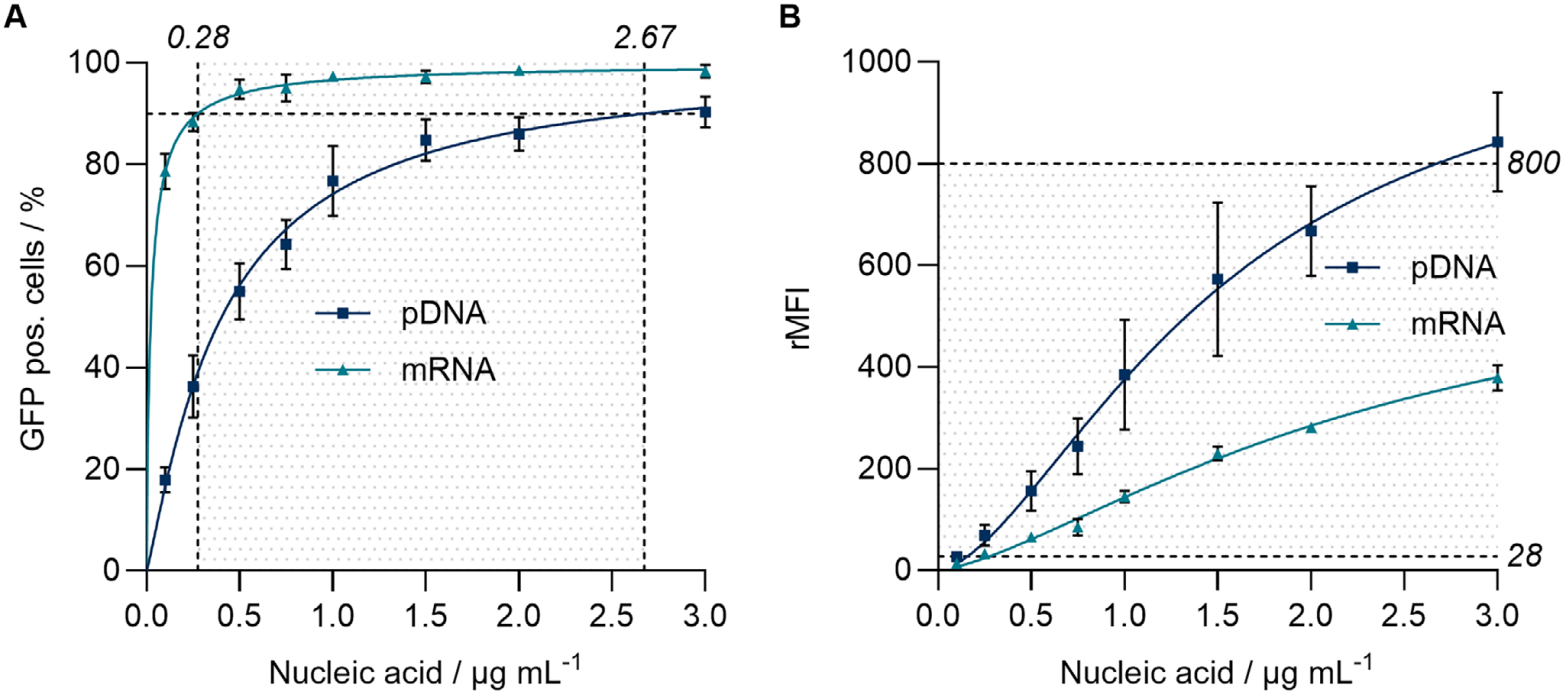
Concentration‐dependent gene expression of pDNA and mRNA. HEK293T cells were transfected with BLNP‐1 containing pDNA (GFP) or mRNA (GFP) and measured after 24 h via flow cytometry. Different concentrations of pDNA and mRNA were applied by diluting the formulation before adding it to the cells. Dotted lines and shade mark (A) nucleic acid concentration where 90% cells are GFP pos. (TC_90_) and (B) rMFI values at TC_90_. Italic numbers represent TC_90_ and rMFI at TC_90_. Data shown as mean ± s.d. (*n* = 3 biological replicates). Connecting lines derived by a nonlinear regression conducted with GraphPad Prism.

Addressing diseases prevalent in modern societies, gene therapy, particularly CRISPR‐Cas9 has come into spotlight. Different strategies are reported to enable gene editing [66, 67] introducing Cas9 by plasmids, mRNA, or the delivery of the ribonucleoprotein combined with the specific sgRNA [18, 68]. Given the gene delivery potential of BLNP‐1, the delivery of mRNA and pDNA was investigated in more detail to elucidate which nucleic acid is favored for successful CRISPR‐Cas9 strategy. In detail, dose‐response GFP expression was investigated via flow cytometry in HEK293T cells in the range of 0.10–3.00 µg mL^−1^ nucleic acid (Figure 4). The results demonstrate a notable distinction in transfection efficiency. Delivery of mRNA (GFP) induced gene expression in nearly 100% of the cells at significantly lower nucleic acid concentrations than pDNA. Using mRNA, 90% of the cells were transfected at 0.28 µg mL^−1^ (TC_90_) rendering mRNA to be tenfold more efficient than pDNA (GFP) (Figure 4A, TC_90_ = 2.67 µg mL^−1^). However, pDNA exhibited significantly higher protein production at a starting concentration of 0.75 µg mL^−1^ (Figure 4B, rMFI). This can be explained by the transcription of pDNA resulting in a larger number of mRNA molecules at the cellular level. A similar behavior has been observed with commercial lipofectamine, which also reveals increased protein expression with pDNA [69].

Another advantage of mRNA is the rapid intracellular accumulation of the expressed protein compared to pDNA. A preliminary experiment demonstrated that 90% of the cells were transfected within 2.9 h using mRNA, which is comparable to the conventional LNP standard (2.3 h) (Figure S11) [39]. In contrast, pDNA required 18.1 h to reach a similar level of transfection. Additionally, differences in transfection homogeneity were observed, as reflected in the fluorescence intensity histograms. Narrower distributions indicate more uniform transfections across the cell population. Although pDNA resulted in slightly higher overall expression levels, the signal exhibited a more diffuse cellular distribution (Figure S12). This finding is consistent with that reported by the Kataoka research group [70].

Based on the kinetic studies, we conclude that mRNA is a superior choice to pDNA for our CRISPR‐Cas9 strategy. The fact that lower amounts of nucleic acid are sufficient to transfect the majority of cells is favorable [71]. Furthermore, the time‐dependent aspect may be critical when delivering two nucleic acids, Cas9 and the corresponding sgRNA, simultaneously. Delayed Cas9 expression, as observed with pDNA, could prevent the formation of functional Cas9‐sgRNA ribonucleoprotein complexes (RNP) in a timely manner. The use of mRNA is also favored by its approval status, degradation potential, and the reduced immunogenicity by nucleotide modifications [72].

### 2.3 CRISPR‐Cas9 Application With BLNP

**FIGURE 5:**
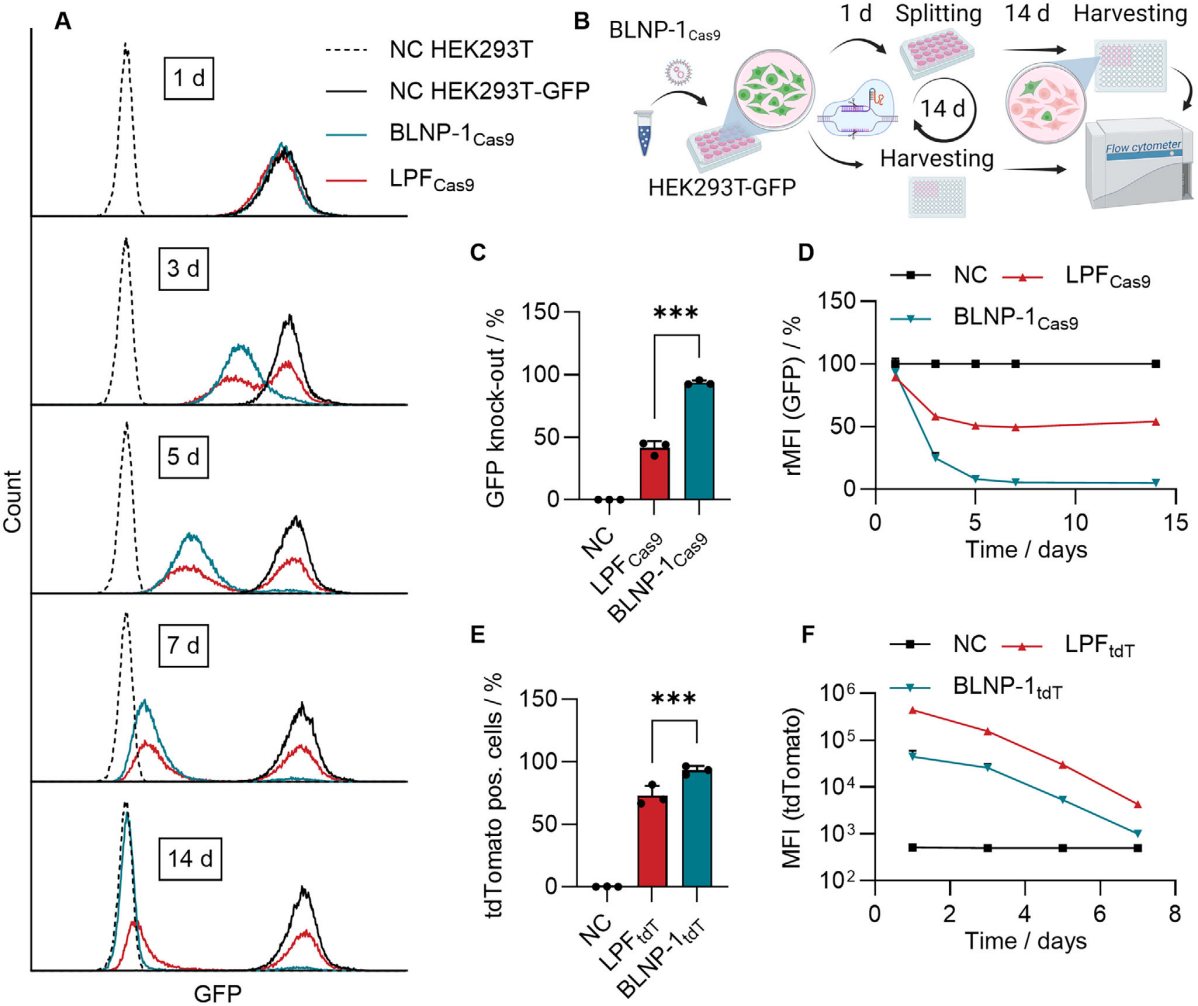
CRISPR‐Cas9 knockout of GFP with BLNP. (A) Histograms of viable single cells analyzed via flow cytometry after 1–14 days of incubation. Histograms are normalized to 10 000 events. Counts are represented as the means of three biological replicates. (B) CRISPR‐Cas9 assay scheme, HEK293T‐GFP cells were treated with BLNP‐1 loaded with Cas9 mRNA and anti‐GFP sgRNA (BLNP‐1_Cas9_), incubated for 14 d and measured in between. (C) Knockout efficiency after 14 d of incubation. (D) rMFI reduction over time. The rMFI/% values are obtained by normalization to the control (black). (E,F) Transfection of HEK293T cells with tdTomato mRNA (tdT). tdTomato pos. cells after 24 h (E) and tdTomato MFI decay over time (F). NC represents negative controls using free nucleic acid without nanoparticles. Lipofectamine (LPF) was used as positive control. Data shown as mean (A, D, F) and mean + s.d. (C, E) (*n* = 3 biological replicates, individual data points applied). ***p ≤ 0.001 derived from a two‐way analysis of variance (ANOVA).

Since the sgRNA and the Cas9 protein must be present in the same cell, co‐delivery is envisaged, particularly regarding potential in vivo applications [73]. Thus, Cas9 mRNA as well as sgRNA matching the target gene (GFP) were co‐formulated. GFP expression stable HEK293T cells (HEK293T‐GFP) were treated with BLNP‐1 containing Cas9 mRNA and sgRNA (BLNP‐1_Cas9_) and analyzed by flow cytometry. Different time points were aimed for up to 14 days (Figure 5B). Lipofectamine messenger max was used as a positive control (LPF_Cas9_). The fluorescence of treated cells was reduced in a time‐dependent manner using BLNP‐1_Cas9_ (Figure 5A). After 14 days, the fluorescence of cells corresponded to the standard HEK293T cells (dashed line). BLNP‐1_Cas9_ treatment induced a knockout in 94% of all single cells leading to a total rMFI reduction of 95%, which is significantly higher than those obtained with LPF_Cas9_ (42% knockout) (Figure 5C,D). This high knockout efficiency is comparable to or surpasses that of other LNP studies investigating knockout effects in vitro [74].

To further distinguish the influence of expressed proteins (MFI, rMFI), an additional transfection evaluation was added to the experimental setup. Thus, standard HEK293T and HEK293T‐GFP were seeded and treated in parallel, necessitating the change of the reporter gene to tdTomato (tdT). Since LPF commonly results in high MFI values, it was used as a control [18]. LPF_tdT_ revealed a 10‐fold increase in the MFI compared to BLNP‐1_tdT_. (Figure 5F). Taking into account that LPF_tdT_ transfected fewer cells (Figure 5E, 72% compared to 93%), it can be assumed that transfection efficiency proves to be more relevant than high protein numbers for certain therapeutic approaches, such as CRISPR‐Cas9 gene editing. This underscores the rationale that protein expression needs to be tailored for specific applications, as high expression levels are not necessarily required. Another disadvantage of high performing vectors in terms of protein levels is the slowed down turnback to a basal level after a potential treatment, proven by Figure 5F.

Mutations most likely vary from patient to patient, which makes it less attractive to target them with conventional large‐scale batch‐to‐batch drugs, making personalized medicine a powerful tool to overcome this hurdle [20, 75, 76, 77, 78]. It was shown that BLNP can be produced reproducible on small scale with high CRISPR‐Cas9 knockout efficiencies (relative s.d. < 2% of three independent biological replicates). This requirement could be critical for personalized medicine, especially since it can be implemented in local health centers rather than large industrial facilities, where a one‐size‐fits‐all therapy approach is ineffective [77, 79].

### 2.4 Transfection of Primary Human Monocytes

Primary cells of the immune system represent a key player in various diseases [80] and particularly in life‐threatening infection and sepsis that is characterized as dysregulated host response, including immune response, to infection [14]. These are a diverse group of disorders characterized by host specific origins. Besides T‐cells, monocytes, as direct precursors to macrophages, are at the center of interest for immune therapy [81].

**FIGURE 6:**
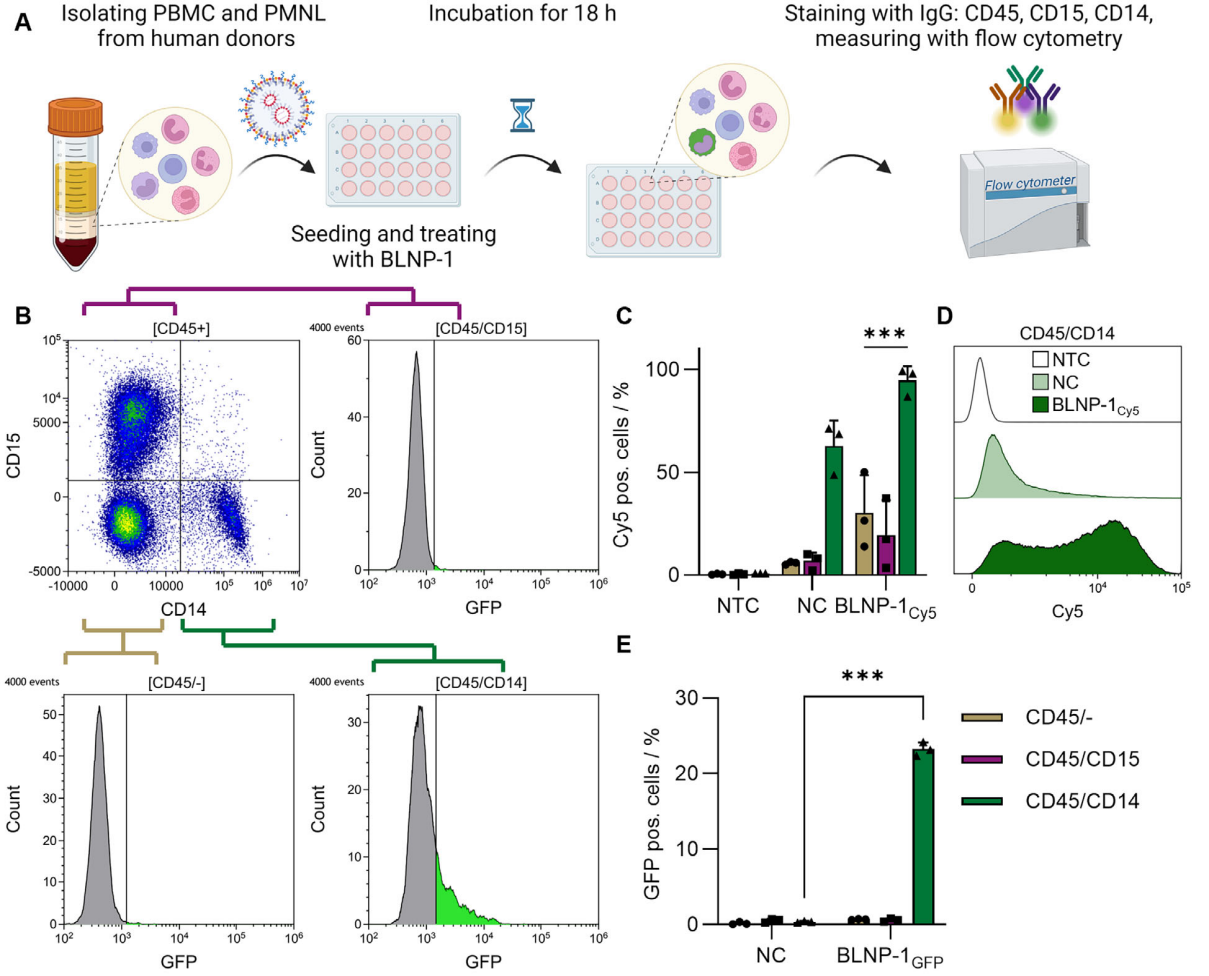
Transfection and uptake of BLNP‐1 in human primary leukocytes. (A) Workflow of transfection of human leukocytes with BLNP‐1 (GFP mRNA). (B) Whole CD45 positive cells get separated by staining for CD15 and CD14 and separately analyzed. C Uptake was measured by using a Cy5‐labeled mRNA via flow cytometry analysis after 4 h. Buffer treated cells (NTC) and free mRNA treated cells (NC) were used as negative controls. Data shown as mean + s.d. (*n* = 3 biological replicates; individual data points applied). (D) Fluorescence intensity distributions (Cy5) of CD14^+^ cells. Histograms represent the mean of *n* = 3 biological replicates. The data reveal an increased uptake of Cy5‐labeled mRNA when encapsulated in BLNP‐1_Cy5_ compared to free mRNA (NC). (E) GFP pos. cells analyzed by flow cytometry. Data shown as mean + s.d. (*n* = 3 biological replicates; individual data points applied). ***p ≤ 0.001 derived from a two‐way analysis of variance (ANOVA).

To evaluate the potential of BLNP‐1 for these targets, the gene delivery potential to primary human cells was investigated. Peripheral blood mononuclear cells (PBMC) and polymorphonuclear leukocytes (PMNL) were isolated from healthy volunteers (Figure 6A) and treated with BLNP‐1 encapsulating GFP mRNA ex vivo. After separation via flow cytometry using different cell‐specific antibodies targeting surface markers (cluster of differentiation, CD) for CD45, CD15, and CD14 (Figure 6B), significant mRNA expression was observed exclusively in CD14^+^ monocytes (Figure 6E). Additional uptake studies utilizing a Cy5‐labeled mRNA (BLNP‐1_Cy5_) revealed that the CD14^+^ monocytes are the predominant target of BLNP‐1 (Figure 6C, 95% Cy5 pos. cells). Lymphocytes (30% Cy5 pos. cells) and neutrophils (CD15^+^) (20% Cy5 pos. cells) showed reduced Cy5 signals. This is remarkable since monocytes only represent 8% of the total leukocyte count whereas neutrophils comprise 50% and have a pronounced phagocytosis activity [82]. The more pronounced phagocytic behavior of neutrophils may also explain the nearly absent mRNA translation observed in these cells, as phagocytosis is typically associated with rapid intracellular degradation. In contrast, monocytes possess endocytic capabilities that may favor intracellular processing pathways compatible with mRNA translation [83]. The literature describes several strategies for targeting monocytes, including passive approaches based on particle size or surface charge, as well as active targeting via specific receptors such as folate or scavenger receptors [83, 84]. However, in the present study serum was present in the assay environment, which likely promoted protein corona formation on the particle surface. This may have facilitated recognition and uptake via Fc receptors expressed by the cells [85]. The interaction of BLNP‐1 with serum proteins was further supported by hemolysis assays, in which red blood cells (RBCs) were exposed to high concentrations of PFNs. Hemolytic effects were observed in the absence of serum, whereas no hemolysis occurred in the presence of 10% fetal calf serum (FCS) or in whole blood measurements (Figure S13). Notably, free mRNA (NC) was also taken up by CD14^+^ monocytes; however, examining the fluorescence intensity, BLNP‐1 showed an advantage (Figure 6D). The successful gene transfer into primary human monocytes suggests that a potential application of CRISPR‐Cas9 is also possible. This opens a wide range of potential therapeutic applications since gene editing recently went into focus for clinical translation [16, 19].

Ex vivo treatments are also a viable approach, as demonstrated in studies using LNPs for T‐cell engineering. In this process, T cells are genetically modified outside the body to enhance their therapeutic properties, such as by introducing receptors that improve the recognition and elimination of cancer cells [86]. LNP‐based immunotherapy also offers a wide range of applications. For instance, treated macrophages can be utilized in tumor therapy [87].

To further evaluate cell toxicity in immune cells, a LDH assay was conducted using the previously described PBMCs and PMNLs. The results showed no significant LDH release for either BLNPs or conventional LNPs. Similarly, cytokine profiling revealed no noteworthy abnormalities. Only moderate IL‐1β release was detected in two donors for the LNPs, while TNFα and IL‐6 levels were consistently negative, supporting their low immunogenicity (Figure S14). Additional evidence supporting the biocompatibility of CHEMS‐PMeOx_52_ is provided by Simon et al., who reported no impairment of hepatic function following intravenous administration of POxylated vesicles [57].

### 2.5 Blue Formulation for Approved Lipids—In Vitro and In Vivo Studies

The previously described stealth lipid (CHEMS‐PMeOx_52_) constitutes the initial basis for the aqueous formulation. We compared this material with the commercial and FDA approved stealth lipid DMG‐PEG 2000 (DMG‐PEG) instead of CHEMS‐PMeOx_52_. DMG‐PEG was also shown to be suitable for BLNP formulation (BLNP‐2). BLNP‐2 (SM‐102, DSPC, and DMG‐PEG) revealed a smaller diameter (Z‐Average 130 nm, PDI 0.1) compared to BLNP‐1 and fell within the size range the conventional control LNP, independent of the nucleic acid used (Figure S15A,B,S16). Transfection of HEK293T cells was investigated and revealed the efficacy of more than 95% positive cells using GFP mRNA (Figure S15C,D). Of note, both BLNP‐1 and BLNP‐2 showed no adverse effects in vitro (Figure S17). The cytotoxic behavior was assessed using the PrestoBlue assay on L929 cells, and no reduction in their relative metabolic activity was detected. Both particles displayed low zeta potential values below ± 2 mV (Figure S18), which is in the expected range for shielded LNP measured in PBS [88]. This nearly neutral zeta potential is favorable for prolonged circulation time and reduced nonspecific interactions with cellular membranes, which could otherwise increase cytotoxicity, and is characteristic of conventional PEGylated LNPs [89]. Overall, BLNP formulations containing mRNA or pDNA remained physically stable with respect to particle size and PDI during storage at 4°C for at least 12 weeks (Figures S19–S21), and were more stable under these conditions than when stored at –80°C. In particular, freezing induced aggregation of mRNA‐loaded particles, as indicated by an approximately threefold increase in Z‐Average and a PDI > 0.5 (Figure S19A–D). This observation aligns with prior reports showing that LNPs in aqueous suspension typically exhibit greater stability under refrigeration than freezing, presumably due to structural stress associated with freeze‐thaw cycles [90]. Despite this apparent physical stability, storage at 4°C was associated with a gradual loss of functional activity in the BLNP system, as reflected by a time‐dependent decrease in in vitro transfection efficiency (Figure S22). Taken together, these findings suggest that aqueous storage, whether refrigerated or frozen, is not optimal for maintaining long‐term functional performance of BLNP formulations. Strategies to overcome this limitation include the addition of cryoprotectants such as sucrose prior to freezing, as well as freeze‐drying of the nanoparticles [51, 91]. Accordingly, we explored lyophilization of BLNPs, a well‐established method for the stable transport and storage of sensitive biomaterials that has recently gained increasing attention in the context of LNP formulations [92, 93]. As cryoprotectants are essential for this process, we investigated the use of 8.7% sucrose, which has been shown to be effective for LNPs in previous studies [94]. The results clearly demonstrate that BLNPs, particularly BLNP‐2, remain stable in terms of size and dispersity (Figure S23A–C). BLNP‐1 exhibited a slight increase in Z‐Average when sucrose was added (from 170 to 200 nm), which increased further to 220 nm after 1 day of storage and reconstitution in PBS. This effect was less pronounced for BLNP‐2. Notably, samples freeze‐dried without sucrose showed an increase in the PDI, as reflected by the DLS intensity curves (Figure S23A). These findings are consistent with the observed biological performance. Samples without cryoprotectants exhibited nearly complete loss of transfection efficiency, while samples containing sucrose maintained biological activity (Figure S23D,E). However, a slight decrease in transfection efficiency, particularly for mRNA‐loaded particles, was still observed (Figure S23E). Future studies should build on these storage findings with the aim of preserving biological function under practical and simplified storage conditions. At present, freeze‐drying in the presence of a cryoprotectant appears to be the most promising strategy for BLNP formulations, although subsequent storage under frozen conditions remains advisable.

**FIGURE 7:**
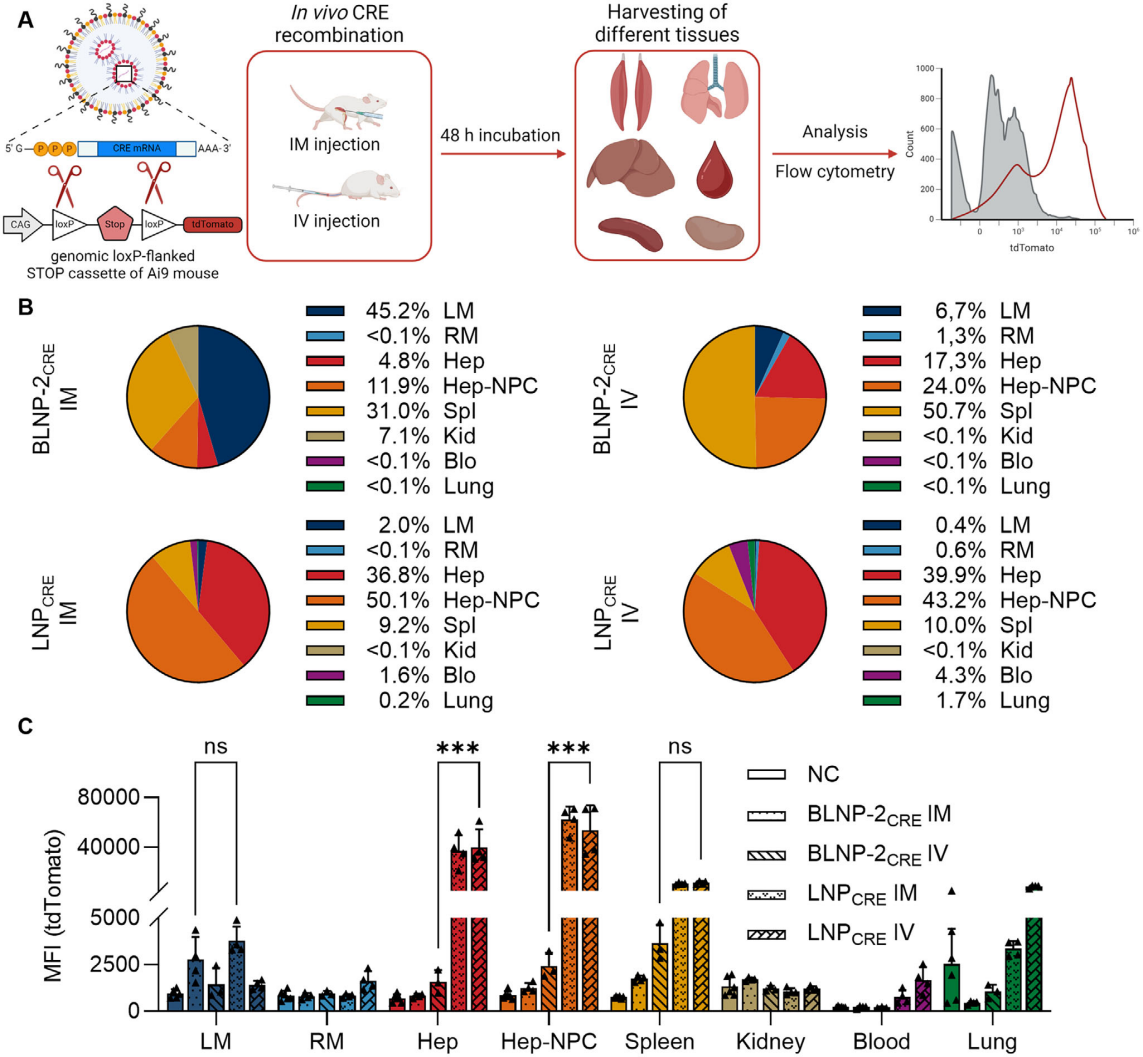
Flow cytometry analysis of different tissues from the Ai9/Cre mouse model. (A) Ai9 mice were treated with 5 µg of CRE mRNA intramuscularly (IM) or intravenously (IV). After 48 h cells from different tissues, including injected muscle (LM), non‐injected muscle (RM), liver (hepatocytes (Hep), hepatic non‐parenchymal cells (Hep‐NPC)) including hepatic immune cells, endothelial cells, stellate cells, and cholangiocytes, spleen (Spl), kidney (Kid), blood (Blo), and lung were collected and analyzed via flow cytometry. A cholesterol‐rich LNP and non‐treated animals were used as controls. (B) Relative tissue distribution for each nanoparticle and injection method. The distribution was calculated using the relative mean fluorescence intensity of single cells. (C) Mean fluorescence intensity (tdTomato) of single cells for non‐treated mice (NC, *n* = 6), BLNP‐2_CRE_ IM (*n* = 4), BLNP‐2_CRE_ IV (*n* = 3), LNP_CRE_ IM (*n* = 4), and LNP_CRE_ IV (*n* = 4). Data shown as mean + s.d. (individual data points applied). ***p ≤ 0.001 derived from a two‐way analysis of variance (ANOVA).

These in vitro results suggest that cholesterol is not essential for efficient mRNA delivery. To further investigate the effect of cholesterol on in vivo expression and biodistribution, we conducted animal studies comparing BLNP‐2 with conventional LNP formulations. The LNP lipid molar composition was analogous to mRNA‐1273 and the preparation was adapted from a previous protocol [39]. The loxP‐flanked tdTomato reporter mouse Gt(ROSA)26Sor^9(CAG‐tdTomato)Hze^/J (Ai9) was employed [95], and nanoparticles containing synthetic Bacteriophage P1 Cyclization recombination (Cre) mRNA were administered one‐sided in the murine hind‐limb muscle (IM) and intravenously (IV). The animals revealed no signs of abnormality in the preclinical score, which included general condition, spontaneous reactivity, and pain. No significant differences in body weight were observed during the experiment (Figure S24). After 48 h, hind‐limb muscles, lung, liver, spleen, kidney, and blood were collected. A single cell suspension was prepared from each tissue and analyzed for its tdTomato expression using flow cytometry to compare the cellular Cre‐recombination between IM and IV administration (Figure 7A).

Following intravenous administration, the strongest signal for the BLNP‐2_CRE_ was detected in splenocytes (Figure 7C). In this tissue, most of the cells (72%, Figure S25) were CD45^+^ leukocytes. For this reason, we conclude that BLNP‐2_CRE_ predominantly transfects these cells. In contrast, the cholesterol‐containing standard LNP was detected predominantly in the liver (Hep, Hep‐NPC). Although transfection was also observed in the spleen, over 80% of the relative detected signals were in hepatic tissue (Figure 7B). This predominant liver expression was also reported in a similar mouse model by Kauffman et al. [95]. Notably, this was the case after both intravenous and intramuscular administration. This represents a significant difference compared to the BLNP‐2_CRE_, which induced significantly less tdTomato expression in the liver. Only 0.2% of hepatocytes appear tdTomato‐positive after intravenous administration of BLNP‐2_CRE_, compared to 2.4% with LNP_CRE_ (Figure S26), representing a 10‐fold difference. A connection with cholesterol distribution patterns seems feasible. Cholesterol is reported to induce LNP_CRE_ uptake through ApoE binding, establishing an LDL receptor affinity that enables active uptake [96, 97]. This could explain the high liver affinity of conventional cholesterol‐containing LNP. The effect of reduced liver expression after decreasing the amount of cholesterol was also demonstrated by Kawaguchi et al. [98]. A reduction in the molar amount of cholesterol led to decreased liver expression. BLNP‐2_CRE_ contains no cholesterol, which may explain the reduced liver tropism. Other mechanisms, such as the previously discussed protein‐mediated uptake by immune cells including monocytes, may be more pronounced and could account for the observed splenic expression [85]. Future studies focusing on cell‐specific biodistribution will be necessary to better understand these mechanisms.

Another interesting finding is the signal distribution after intramuscular administration. The local signal intensity in the injected muscle was identical for both BLNP‐2_CRE_ and LNP_CRE_. Locally, the LNP_CRE_ showed no advantage in terms of efficacy. As previously mentioned, after intramuscular application, the efficacy of the LNP_CRE_ is primarily observed in the liver. However, for the success of vaccination, efficacy in hepatocytes is irrelevant, as the transfection of antigen‐presenting cells is crucial for this purpose [99].

## 3 Conclusion

The widespread use of mRNA lipid nanoparticle (LNP) vaccines has brought gene transfer to the forefront of global medical research and generated considerable optimism. However, as this technology advances, challenges related to extrahepatic transport and immune activation will need to be addressed. This emphasizes the benefits of the blue formulation approach enabling the production of potent nanoparticles without significant amounts of cholesterol avoiding the frequently observed hepatic accumulation. BLNPs further represent a promising advancement, facilitating in vivo gene delivery with a streamlined production process. The use of organic solvents, which typically require additional purification steps, is entirely avoided. The production of preformed lipid assemblies also allows small‐scale and flexible approaches, which are advantageous for laboratory screening or personalized medicine in inflammatory disease entities where immune cells are of particular interest. It is possible to produce a higher amount of pre‐assembled BLNP and then separately use flexible with modified experiment or patient‐specific active pharmaceutical ingredients. This is proven by utilizing similar lipid mixtures for the entrapment of different nucleic acids. Future studies should expand to in vivo investigations, including comprehensive pharmacokinetic analyses and in vivo toxicity assessments, to eventually achieve clinical readiness.

Furthermore, these results suggest a re‐evaluation of the role of cholesterol in LNPs, indicating that pure cholesterol is not mandatory for gene expression, in particular not for immune cells. These findings contribute to the evolving understanding of the role of cholesterol in LNPs prompting reconsideration of conventional LNP formulations to enhance understanding of its functions in various contexts. Cholesterol‐related side effects, such as an increased immune response and hepatic accumulation, can be very counterproductive in certain diseases and are avoided by the formulation described here.

## 4 Experimental Section

The detailed experimental section is included in the Supporting Information.

### 4.1 Ethics

Human leukocyte concentrates were provided by the Department of Transfusion Medicine at the University Hospital of Jena, Thuringia, Germany. The transfection on studies on human PBMCs and leukocytes was approved by the local ethical committee (approval no. 5050‐01/17) and was performed in accordance with guidelines and regulations.

All experiments on animals were conducted in accordance with German laws and regulations following protocols approved by the Thuringian State Administrative Office, Thuringia, Germany (Reg. No. UKJ‐20‐013).

### 4.2 Formulation of Blue Lipid Nanoparticles by Sonication (BLNP)

For preparation of BLNP stock solutions of the ionizable lipid SM‐102 (Hölzel, Cat No. DCC‐DC52025‐1 g), the phospholipid DSPC (Avanti, Cat No. 850365P) and the stealth lipids CHEMS‐PMeOx_52_ [55], or DMG‐PEG 2000 (Avanti, Cat No. 880151P) were prepared in 20 mM sodium acetate buffer with a pH value of 5.5 (NaOAc) (Thermo‐Fisher Scientific Inc., Cat No. AM9740). For buffer dilution, UltraPure water (Invitrogen, Cat No. 12 060 346) was used. Prior to formulation, stock solutions were adjusted to room temperature and homogenized by placing them in the ultrasonic bath for 15 min at room temperature (RT). The lipids were then mixed in NaOAc to get the defined lipid molar ratios and treated 2 times for 20 s on ice with an ultrasonic homogenizer (Hielscher Ultrasonics GmbH, Cat No. UP200St) (P 100 W, C 100%, A 20%) to produce unloaded pre‐formed nanostructures (PFN). The particles were incubated for 10 min at room temperature. A mixture of the nucleic acid with a concentration of 120 µg mL^−1^ was prepared in NaOAc (MM). The BLNP were loaded by mixing it with the MM 1 + 1 and incubated for 15 min at room temperature. To receive the final BLNP the mixture was supplemented 1 + 1 with PBS (Capricorn, Cat No. PBS‐1A).

### 4.3 Transfection of HEK293T Cells With pDNA (GFP) and mRNA (GFP)

HEK293T cells (DSMZ, Cat No. ACC 635) were cultivated at 37°C in humidified 5% (v/v) CO_2_ atmosphere (incubation) in Dulbecco's Modified Eagle Medium (DMEM) Low Glucose (1 g L^−1^) (Capricorn, Cat No. DMEM‐LPA). The media was supplemented with 10% (v/v) fetal bovine serum (FBS) (Capricorn, Cat No. FBS‐11A), 100 U mL^−1^ penicillin (Capricorn, Cat No. PS‐B) and 100 µg mL^−1^ streptomycin (Capricorn, Cat No. PS‐B) (culture medium). For the experiments, 0.2 × 10^6^ cells mL^−1^ were seeded in 500 µL culture medium containing 10 mM HEPES (test medium) in a 24‐well plate and cultivated for 24 h. 1 h before the treatment, the medium was changed to 450 µL test medium. Cells were treated with nanoparticles prepared as described above with pDNA (Addgene, Cat No. 54 767, isolated with the EndoFree Plasmid Mega Kit (5) (Qiagen Cat No. 12 381)) or PureBoost Modified (m1Ψ) EGFP mRNA (Cellerna, Cat No. 2001M‐1MG) to get a final nucleic acid concentration of 3.0 µg mL^−1^. To achieve concentrations below 3.0 µg mL^−1^, BLNP was diluted with a 1 + 1 mixture of PBS (Capricorn, Cat No. PBS‐1A) and 20 mM NaOAc buffer (pH 5.5). After 24 h incubation, the supernatant was transferred to a fresh 24‐well plate. The cells were immediately detached with trypsin‐EDTA, resuspended in the corresponding supernatant, and analyzed by flow cytometry (CytoFLEX, Beckman Coulter, U.S.). At least 10 000 events were measured, and viable single cells were analyzed by forward and sideward scatter (FSC/SSC). Fluorescence was measured at *λE*_x_ = 488 nm with a 525/40 nm bandpass filter (FITC channel). Positive cells were identified by gating against the cells treated with only nucleic acid (MM). A detailed gating strategy is provided in the supporting information (Figure S27). Analysis was conducted with CytExpert V 2.5.0.77.

### 4.4 GFP Knockout in HEK293‐GFP Stable Cells Using CRISPR‐Cas9

To evaluate knockout efficiency with CRISPR‐Cas9, HEK293‐GFP stable cells (AMSBIO, Cat No. SC001) were used. 0.2×10^6^ cells mL^−1^ were seeded in 500 µL test medium in a 24‐well plate and cultivated for 24 h. 1 h before the treatment, the medium was changed to 450 µL fresh test medium. Cells were treated with 50 µL BLNP‐1_Cas9_ prepared as described before, containing 15 µg mL^−1^ mRNA (Cas9) (OZ Biosciences, Cat No. MRNA25‐100) and 15 µg mL^−1^ sgRNA (Anti‐GFP) (Synthego, Cat No. GRWCY6G; G*G*U*CUACAAGACCCGCGCCG + modified Scaffold) to get a final nucleic acid concentration of 3.0 µg mL^−1^. sgRNA sequence was determined by using CRSIPOR web tool [100]. Additional details are provided in the Supporting Information (sgRNA information). Reference cells were additionally treated with BLNP‐1_tdT_ containing 30 µg mL^−1^ mRNA (tdTomato) (OZ Biosciences, Cat No. MRNA2‐100) to observe transfection. Furthermore, cells were treated with Lipofectamine MessengerMAX (Thermo Fisher Scientific Inc., Cat No. LMRNA001) as positive control (LPF) containing the corresponding nucleic acids as described above and manufactured as described by the manufacturer. After 24 h incubation, the supernatant was removed. The cells were immediately detached with trypsin‐EDTA and resuspended in 1 mL culture medium. 500 µL of the respective well were transferred to a fresh 24‐well plate (split 1 + 1) and cultivated further. The remaining cells were analyzed by flow cytometry (1 d). At least 10 000 events were measured, and viable single cells were analyzed by forward and sideward scatter (FSC/SSC). Fluorescence was measured at *λE*_x_ = 488 nm with an 510/20 nm bandpass filter (with signal attenuation) (B510‐GFP‐ND1 channel) (GFP) and at *λE*_x_ = 561 nm with an 585/42 nm bandpass filter (Y585‐PE channel) (tdTomato). Cells were cultivated further for 14 days and additionally measured as described above after 3, 5, 7, and 14 days. During the initial 7 days, the cells were consistently split into a 1:1 ratio. Subsequently, from day 7 onward, splitting was adjusted to prevent exceeding 90% confluence until day 14. A detailed gating strategy is provided in Figure S28. Analysis was conducted with Kaluza V 2.2.1.

### 4.5 Transfection of Human Primary PBMC and PMNL With BLNP‐1

The PBMC and PMNL seeded as described in the Supporting Methods (Cell Isolation and Culture of Human Primary PBMC and PMNL) were treated with BLNP‐1 containing mRNA (GFP) prepared in a similar way as for the transfection of HEK293T cells. After 18 h of incubation the cells were centrifuged for 5 min, 400 rcf. Subsequently, the supernatant was removed gently and 0.2 M PBS‐EDTA (VWR, Cat No. A2937 0500) was added to detach adherent cells. After 3 min of incubation, cells were transferred to 96‐well plates (V‐bottom) and centrifuged for 5 min at 4°C, 400 rcf. The supernatant was removed, and the cells were washed with BD Pharmingen Stain Buffer (FBS) (BD, Cat No. 554 656) (FACS‐buffer). Antibody cocktail (ABC) containing PE‐CD45 (BioLegend, Cat No. 304 039), PerCP‐CD15 (BioLegend, Cat No. 323 018), and APC‐CD14 (BioLegend, Cat No. 301 808) antibodies was prepared in FACS‐buffer and after an additional washing step 100 µL of ABC was added to each well and the plates were incubated in the dark for 30 min at 4°C. After incubation 100 µL FACS‐buffer was added immediately and cells were resuspended and centrifuged again 5 min at 4°C, 400 rcf and washed with FACS‐buffer and measured with flow cytometry. Viable single cells were identified by FSC/SSC. 488 nm laser was applied with a 510/20 nm bandpass (with signal attenuation) filter to detect GFP and a 690/50 nm bandpass filter to detect CD15. 561 nm laser was applied with a 585/42 nm bandpass filter to detect CD45 and 638 nm laser was applied with a 660/10 nm bandpass filter to detect CD14. A detailed gating strategy is provided in Figure S29. Analysis was conducted with Kaluza V 2.2.1.

### 4.6 Uptake Studies in Human Primary PBMC and PMNL With BLNP‐1

The PBMC and PMNL seeded as described above were treated with BLNP‐1 prepared as described before, loaded with Cy5 labeled mRNA (OZ Biosciences, Custom made) (BLNP‐1_Cy5_). Measurement after 4 h was done as described above by replacing the CD14 antibody with a BV510‐CD14 (BioLegend, Cat No. 367 124), detected using a 405 nm laser with a 525/40 nm bandpass filter. The uptake was detected by measuring the Cy5 signal in viable single cells by applying a 638 nm laser using a 660/10 bandpass filter. A detailed gating strategy is provided in Figure S30. Analysis was conducted with Kaluza V 2.2.1.

### 4.7 Animals

B6.Cg‐Gt(ROSA)26Sor^9(CAG‐tdTomato)Hze^/J (Ai9) mice (RRID: IMSR_JAX:007909) were obtained from Jackson Laboratory (USA) and bred homozygous in the animal facility of the Jena University Hospital, Germany. This strain features a loxP‐flanked STOP cassette that is strategically placed to inhibit the CAG‐promoter driven transcription of tdTomato, a variant of the monomeric red fluorescent protein. When a Bacteriophage P1 Cre recombinase is expressed in Ai9 mice, the cells expressing the Cre undergo a significant change: the STOP cassette is excised in the tissues where Cre recombinase is active, leading to robust tdTomato fluorescence in those specific areas [101]. The animals were housed in a 12 h light/dark cycle at 22°C with 40%–50% humidity. They were fed a standard rodent chew with free access to acidified drinking water. The genotypes of all animals were confirmed. Therefore, DNA was extracted from ear punches and amplified using the KAPA mouse genotyping Kit (Merck, #KK7302). Primers for the wildtype allele (forward: 5’‐ AAGGGAGCTGCAGTGGAG TA‐3’, reverse: 5’‐ CCGAAAATCTGT GGGAAGTC‐3’), mutant allele (forward: 5’‐ GGCATTAAAGCAGCGTATCC‐3’, reverse: 5’‐ CTG TTCCTGTACGGCATGG‐3’) were used. The PCR was performed with the following settings: initial denaturation (94°C, 5 min) with 28 cycles amplification (94°C × 30 s – 58°C × 30s – 72°C × 30 s), followed by a final extension at 72°C for 5 min to ensure complete extension of any remaining single‐stranded DNA, end with indefinite hold at 4°C with the thermocycler of Biometra TAdvanced (Analytik Jena, Jena, Germany). The amplified products were analyzed by agarose gel electrophoresis with 0.5 µg/mL ethidium bromide in a 2% conventional agarose gel in Tris‐Acetate‐EDTA (TAE) buffer (0.04 M Tris, 0.02 M Acedic Acid, 1 mM EDTA in type 1 water; pH 8.3) (all materials from Carl Roth, Germany) and visualized under UV light with G:Box chemi XRQ (Syngene) to confirm the targeted band for the mutant at 196 bp.

### 4.8 In Vivo Transfection of Ai9 Mice

8–12 weeks old male and female Ai9 mice were used for the experiments. Groups of 2 males and 2 females were injected intramuscularly or intravenously with 5 µg mRNA formulated in BLNP‐2_CRE_ or control LNP_CRE_ as previously described in theExperimental Section. The mRNA (OZ Biosciences, Cat No. MRNA26 1000) used encodes for a Bacteriophage P1 Cre recombinase. The injections were carried out under isoflurane anesthesia and post‐Meloxicam analgesia. Mice were incubated and scored for 48 h before sacrificing them for tissue collection and cell isolation with an intraperitoneal injected Ketamine and Xylazin overdose. To generate a baseline, for each experiment, cells isolated from untreated Ai9 mice were included. Whole blood was collected immediately after death via cardiac puncture. A single cell suspension and snap‐frozen specimen for tdTomato protein and its mRNA quantifications were prepared from the lungs, liver, spleen, kidneys, left and right hind‐limb muscle.

### 4.9 Single Cell Isolation and Flow Cytometry

Single cell suspensions were prepared from blood, and all solid tissues were harvested. 100 µL of whole blood was lysed with 1 mL 1X red blood cell (RBC) lysis buffer (Biolegend, Cat No. 420 302) at room temperature for 5 min, and stopped with 9 mL 1X DPBS (Carl Roth GmbH, Cat No. 9150.1). The leukocytes were collected in a pellet by centrifugation (500 rcf, 5 min, room temperature). The RBC lysis was repeated to increase the leukocyte purity. To isolate splenocytes, one third spleen was cut into small pieces, grounded with a plunger, and strained through 100 µm sterilized nylon mesh (laborversand, Cat No. PAS3). The splenocytes were then collected by centrifugation (500 rcf, 5 min, 4°C), resuspended in 1 mL RBC lysis buffer to perform the same lysis processing for the blood, and washed once with staining buffer (5% FBS, 5 nM EDTA in DPBS). Liver, lung, kidney, left and right muscle were cut into small pieces and digested in a 2 mL RPMI1640 (PAN BIOTECH, Cat No. P04‐16515) solution of 0.2 mg/mL collagenase IV (Worthington, Cat No. 44B24 298) at 37°C for 45 min in a 12‐well plate, and stopped with the same volume of RPMI with 10% FBS (Thermo Fisher Scientific, Cat No. 10 270 106) The digested tissue was filtered through a 100 µm nylon mesh. The liver cell suspension was first centrifuged for 5 min at 50 rcf under ambient temperature pelleting the hepatocytes. The supernatant was transferred to a new tube and then centrifuged at 500 rcf for 5 min to collect liver non‐parenchymal cells (NPCs) that include for example, immune cells (e.g., Kupffer cells, Lymphocytes, Stellate cells), and Endothelial cells. Liver NPCs were further lysed same as whole blood and washed once with staining buffer. Suspensions from the lung, kidney, and muscles were centrifuged at 500 rcf for 5 min, followed by the same RBC lysis, and washed one time with staining buffer.

The isolated cells were analyzed via flow cytometry. Single cells were identified by FSC/SSC. 561 nm laser using a 585/42 bandpass filter (PE‐Channel) was applied to detect tdTomato signals (CytoFlex, Beckmann Coulter). A detailed gating strategy for LM and Spleen is provided in Figure S31. Analysis was conducted with Kaluza V 2.2.1 (Beckmann Coulter).

To additionally identify the amount of CD45^+^ cells in the spleen samples additional cell suspensions were incubated with 50 µL Fc‐blocking buffer (Miltenyi, Cat No. 130‐092‐575) at 4°C for 10 min. The blocked samples were then stained with PerCP‐CD45 (Biolegend, Clone: 30‐F11, Cat No. 103 130) at 4°C in the dark for 30 min. After one wash with staining buffer at 500 rcf for 5 min and resuspended in 200 µL stain buffer. PerCP‐CD45 was measured using a 488 nm laser with a 690/50 nm bandpass filter. A detailed gating strategy for naive spleen is provided in Figure S32. Analysis was conducted with Kaluza V 2.2.1 (Beckmann Coulter).

### 4.10 Statistical Analysis

Statistical analyses were performed using GraphPad Prism (version 10.3.1). Data presentation (e.g., mean ± s.d. or mean + s.d.) and statistical analysis methods are described in the figure descriptions. One‐way or two‐way analysis of variance (ANOVA) was performed to compare multiple groups. Statistical significance is indicated as follows: ns *p* > 0.05, *p ≤ 0.05, **p ≤ 0.01, ***p ≤ 0.001.

## Author Contributions

M.S. contributed to conceptualization, data curation, formal analysis, investigation, methodology, visualization, and writing the original draft. N.L. contributed to writing the original draft, data curation, and investigation. L.S. contributed to writing, reviewing, and editing. F.A. contributed to writing, reviewing, editing, data curation, and investigation. V.B. contributed to writing, reviewing, editing, data curation, and investigation. L.G. contributed to writing, reviewing, editing, data curation, and investigation. S.H. contributed to writing, reviewing, editing, data curation, and investigation. S.S. contributed to writing, reviewing, editing, and supervision. O.W. contributed to writing, reviewing, editing, and supervision. V.L. contributed to writing, reviewing, editing, and supervision. M.M. contributed to writing, reviewing, editing, and supervision. M.B. contributed to writing, reviewing, editing, and supervision. A.T.P. contributed to writing the original draft, methodology, supervision, and conceptualization. U.S.S. contributed to writing, reviewing, editing, supervision, and funding acquisition. A.T. contributed to writing, reviewing, editing, supervision, project administration, funding acquisition, and conceptualization.

## Conflicts of Interest

M.S., A.T., and U.S.S. have submitted a patent application related to the findings presented in this study. The authors declare no conflicts of interest.

## Supporting information




**Supporting Information**: smll73146‐sup‐0001‐SuppMat.docx.

## Data Availability

The data that support the findings of this study are available from the corresponding author upon reasonable request.
