# Supplementary material for: Extrahepatic Gene Editing In Vivo Using Organic Solvent‐Free Lipid Nanoparticles
Source: Small. 2026 Mar 31;22(27):e11489. doi: 10.1002/smll.202511489 (PMC13173322; doi:10.1002/smll.202511489)
Supplement: Supplementary file 1 — Supporting Information: smll73146‐sup‐0001‐SuppMat.docx. [file SMLL-22-e11489-s001.docx]

Extrahepatic Gene Editing *In Vivo* Using Organic Solvent-Free Lipid Nanoparticles

**Supporting Information**

*Michael Streiber^1,2^, Na Liu^3^, Laurianne Simon^4^, Franziska Adermann^1,2^, Vivien Bachmann^5^, Lucas Gath,^1,2^ Stephanie Hoeppener^1,2^, Stephanie Schubert^1,2^, Oliver Werz^2,5^, Vincent Lapinte^4^, Marie Morille^6,7^, Michael Bauer^2,3,8^, Adrian T. Press^2,3,8,9^, Ulrich S. Schubert^1,2,^*, Anja Traeger^1,2,^**

^1^Friedrich Schiller University Jena, Laboratory of Organic and Macromolecular Chemistry (IOMC), Humboldtstrasse 10, 07743 Jena, Germany

^2^Friedrich Schiller University Jena, Jena Center for Soft Matter (JCSM), Philosophenweg 7, 07743 Jena, Germany

^3^Jena University Hospital, Department of Anesthesiology and Intensive Care Medicine,

Am Klinikum 1, 07747 Jena, Germany

^4^ICGM, Univ. Montpellier, CNRS, ENSCM, Montpellier, France

^5^Friedrich Schiller University Jena, Department of Pharmaceutical/Medicinal Chemistry, Institute of Pharmacy, Philosophenweg 14, 07743 Jena, Germany

^6^Institut universitaire de France (IUF), Paris, France

^7^IRMB, Univ Montpellier, INSERM, Montpellier, France

^8^Jena University Hospital, Center for Sepsis Control and Care, Am Klinikum 1, 07747 Jena, Germany

^9^Friedrich Schiller University Jena, Medical Faculty, Kastanienstr. 1, 07747 Jena, Germany

*Correspondence to U. S. Schubert (ulrich.schubert@uni-jena.de) and A. Traeger (anja.traeger@uni-jena.de)

## **Supplementary Figures and Tables**

**Table S1. Overview of the used lipids for BLNP-1 in comparison to LNP mRNA‑1273.**

| Lipid | Lipid type | HLB | Structure | LNP | BLNP-1 |
| --- | --- | --- | --- | --- | --- |
| SM-102 | Ionizable lipid | 5.7* |  | 50.0 mol% | 81.3 mol% |
| Cholesterol | Helper lipid | 1.6* |  | 38.5 mol% | - |
| DSPC | Phospho-lipid | 7.2** |  | 10.0 mol% | 16.3 mol% |
| DMG-PEG | Stealth lipid | 17.1* |  | 1.5 mol% | - |
| CHEMS-  PMeOx_52_ | Stealth lipid | 15.3* |  | - | 2.4 mol% |

Hydrophilic lipophilic balance value (HLB) describes the hydrophobicity of molecules. * The calculation of HLB was performed with the freeware MarvinSketch V 23.4 based on the calculation method described by Griffin.[1, 2] ** HLB provided by Fukuhira *et al*.[3]


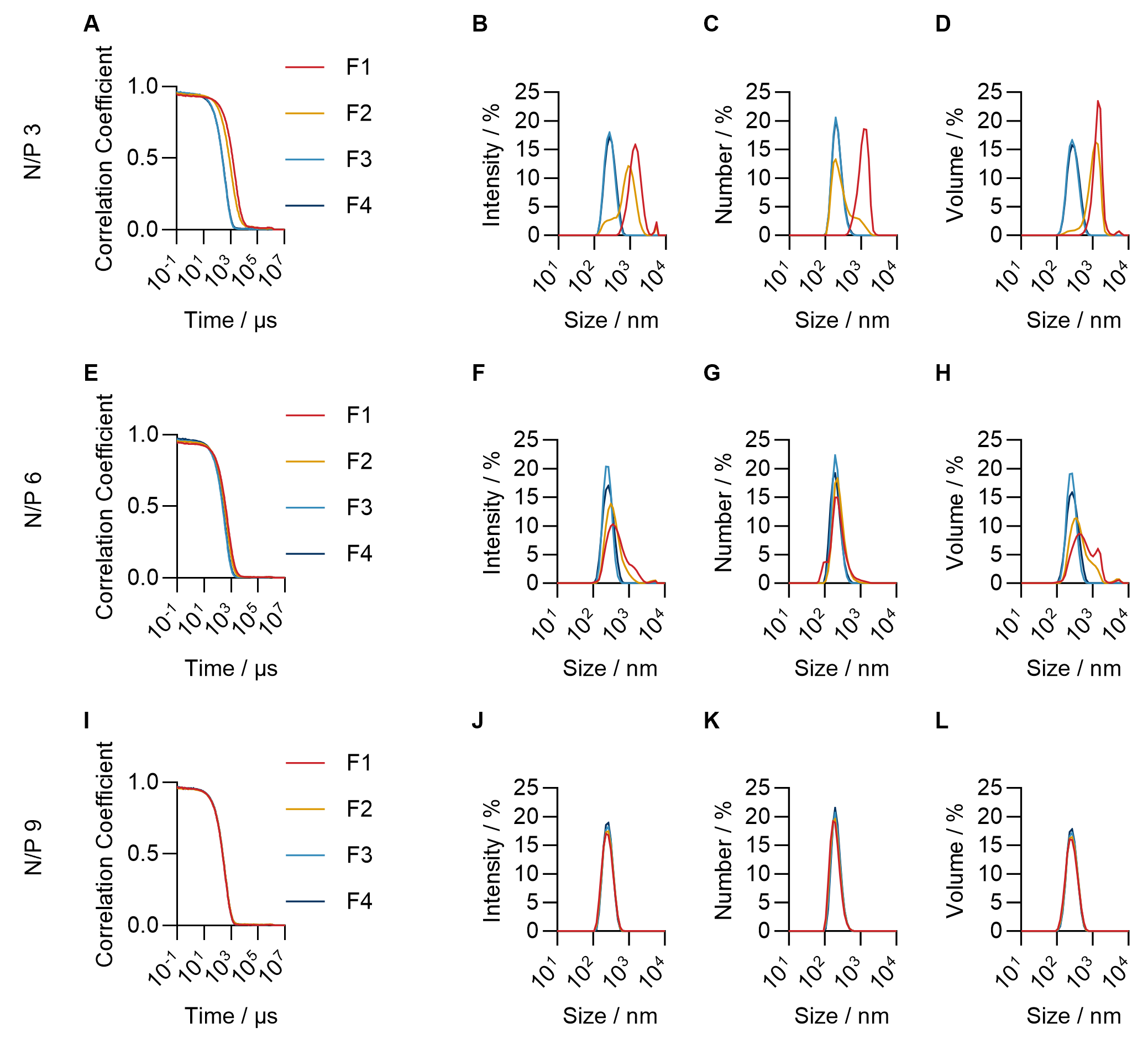


Figure S1. **Correla**tion **coefficient and size distribution of F1 to F4.** F1-F4 measured with DLS. Data shown is supplementary to Fig. 2. **A-D** Correlation coefficient and size distribution by intensity, number, and volume at N/P 3. **E-H** Correlation coefficient and size distribution by intensity, number, and volume at N/P 6. **I-L** Correlation coefficient and size distribution by intensity, number, and volume at N/P 9. Data shown as mean (n = 3 replicates).

**
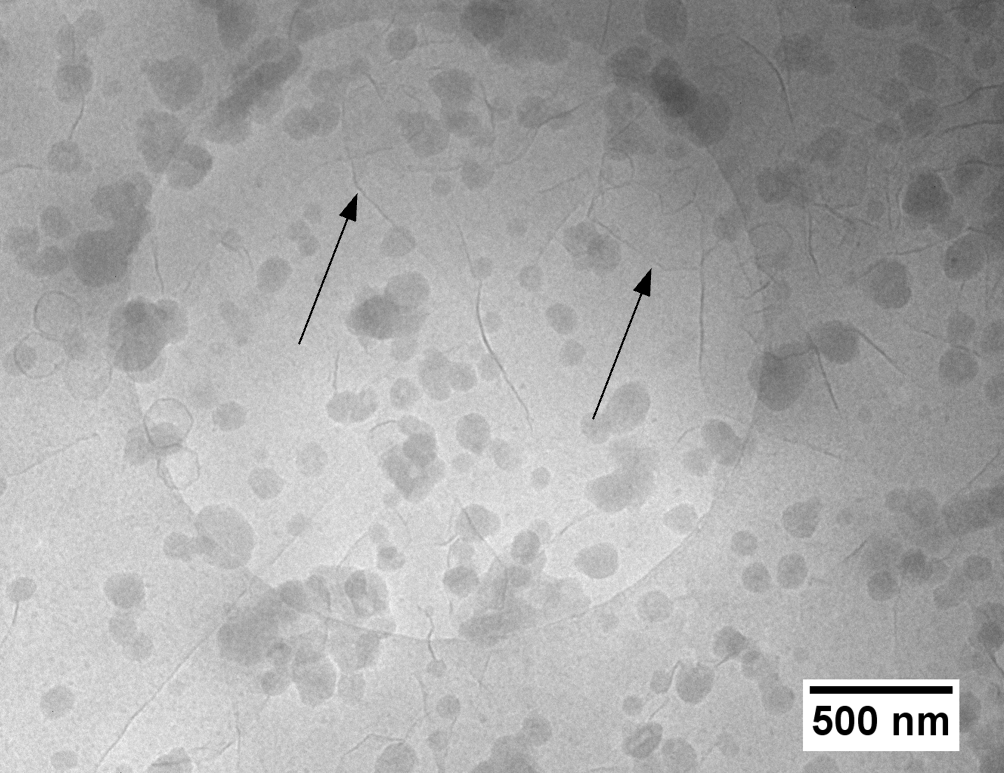
**

**Figure S2. Cryo-TEM image of aqueous vortex lipid formulation.** Aqueous lipid formulation containing SM-102, DSPC and CHEMS-PMeOx_52_ revealing sheets (indicated by the arrows) and other inhomogeneities.

**
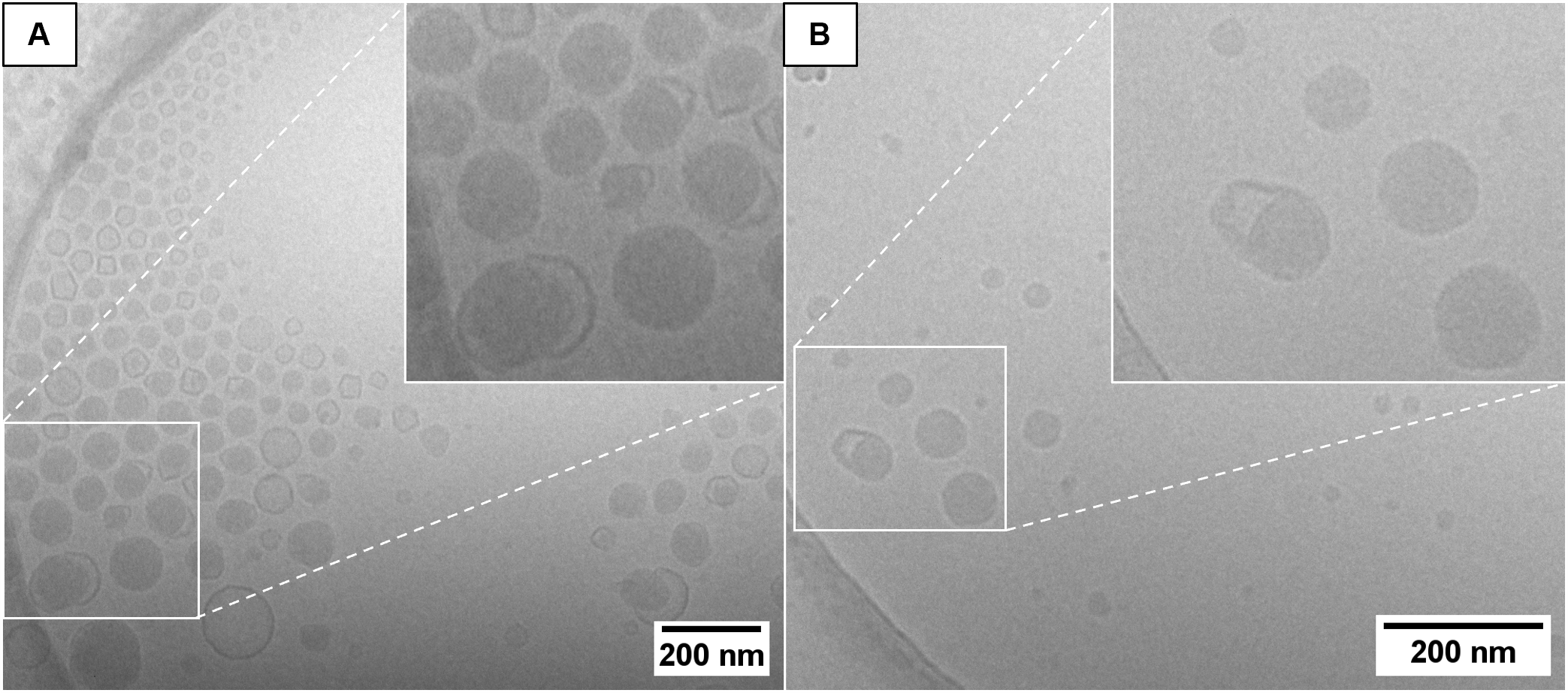
**

**Figure S3. Cryo-TEM image of BLNP. A** BLNP-1 (CHEMS-PMeOx_52_ as stealth-lipid) in aqueous buffer. 2.7 mM total lipid. **B** BLNP-2 (DMG-PEG as stealth lipid) in aqueous buffer. 2.7 mM total lipid. Spherical, compact nanoparticles are prevailed, also some vesicular structures.


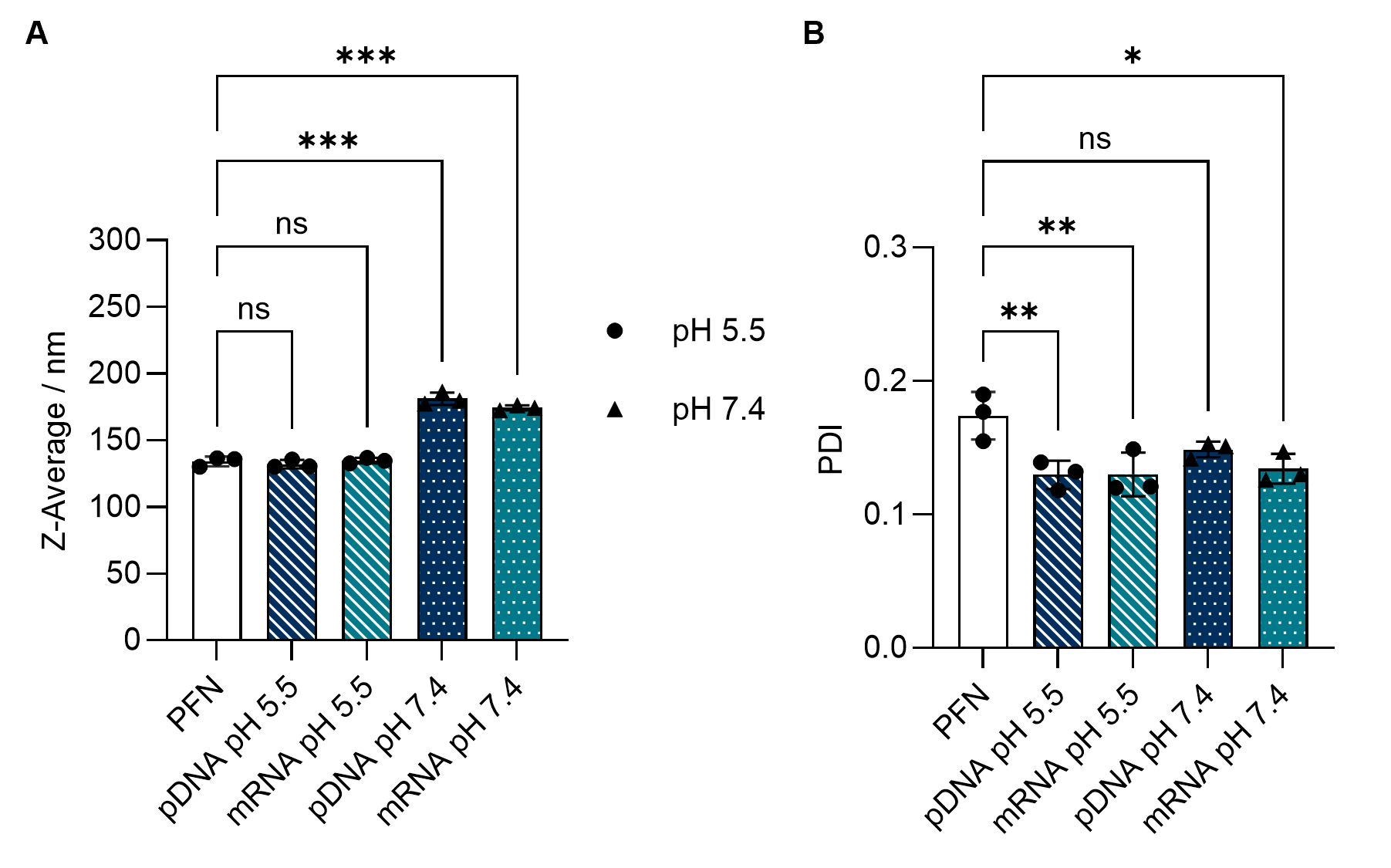


**Figure S4. DLS measurements of BLNP-1 at different formulation stages. A** Z‑Average of BLNP-1 unloaded (PFN), loaded with pDNA (GFP) and mRNA (GFP) at pH 5.5 prior to pH change and loaded with pDNA (GFP) and mRNA (GFP) at pH 7.4 revealing a significant size increase due to pH change. **B** PDI of BLNP-1 unloaded (PFN), loaded with pDNA (GFP) and mRNA (GFP) at pH 5.5 prior to pH change and loaded with pDNA (GFP) and mRNA (GFP) at pH 7.4. Data shown as mean ± s.d., individual data points applied (n = 3 replicates). * p ≤ 0.05, ** p ≤ 0.01 *** p ≤ 0.001, derived from a one-way analysis of variance (ANOVA).


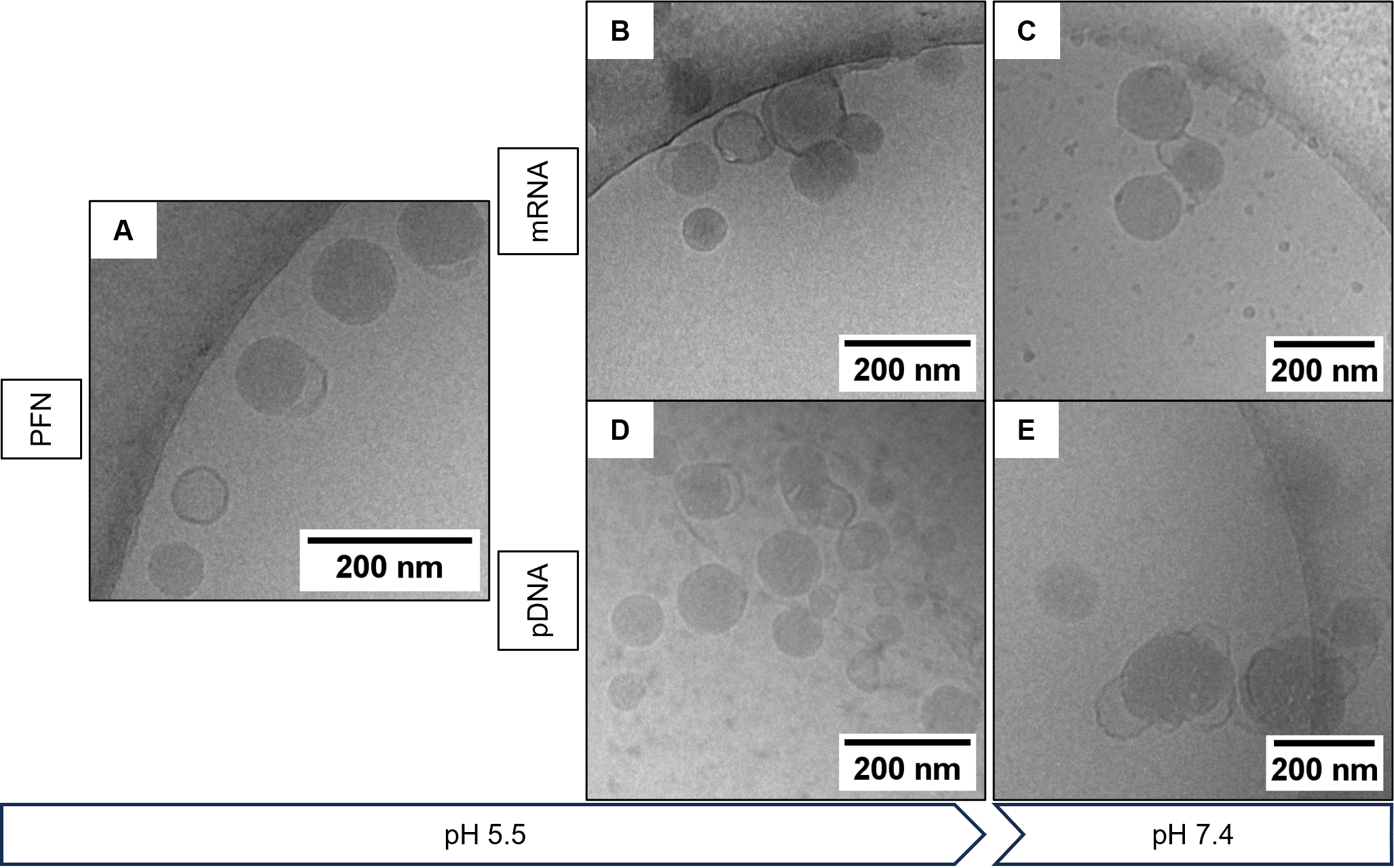


**Figure S5. Cryo-TEM image of BLNP-1 at different formulation stages. A** BLNP-1 prior to nucleic acid entrapment in aqueous buffer (PFN) at pH 5.5. **B** BLNP-1 loaded with mRNA (GFP) prior to pH change. **C** BLNP-1 loaded with mRNA (GFP) at pH 7.4. **D** BLNP-1 loaded with pDNA (GFP) prior to pH change. **E** BLNP-1 loaded with pDNA (GFP) at pH 7.4. Spherical, compact nanoparticles are prevailed, also some vesicular structures. All formulations were adjusted to 0.675 mM total lipid concentration.


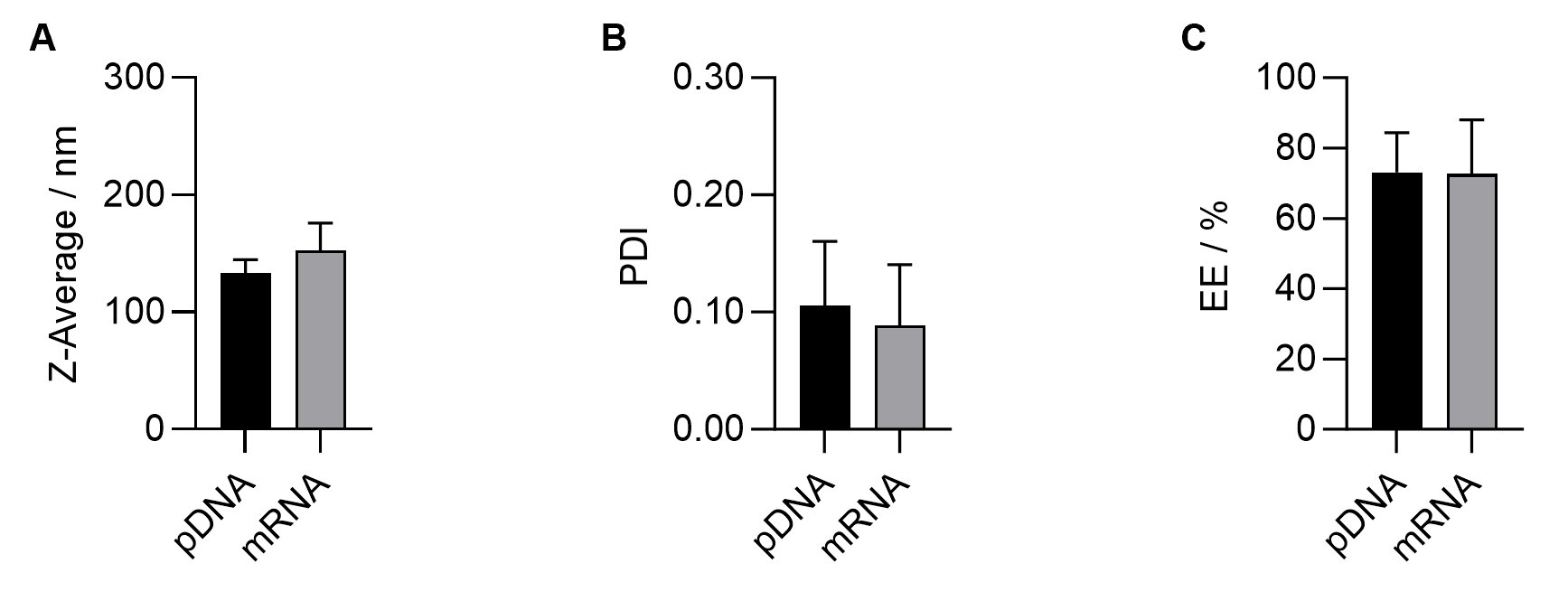


**Figure S6. Characterization of LNP.** LNP containing SM-102/Cholesterol/ DSPC/DMG-PEG (50/38.5/10/1.5). **A, B** DLS measurements (Z-Average, PDI) of LNP encapsulating pDNA (GFP) or mRNA (GFP) after purification. **C** Encapsulation efficiency (EE) determined by nucleic acid encapsulation efficiency assay. Data shown as mean + s.d. (n = 3 replicates).


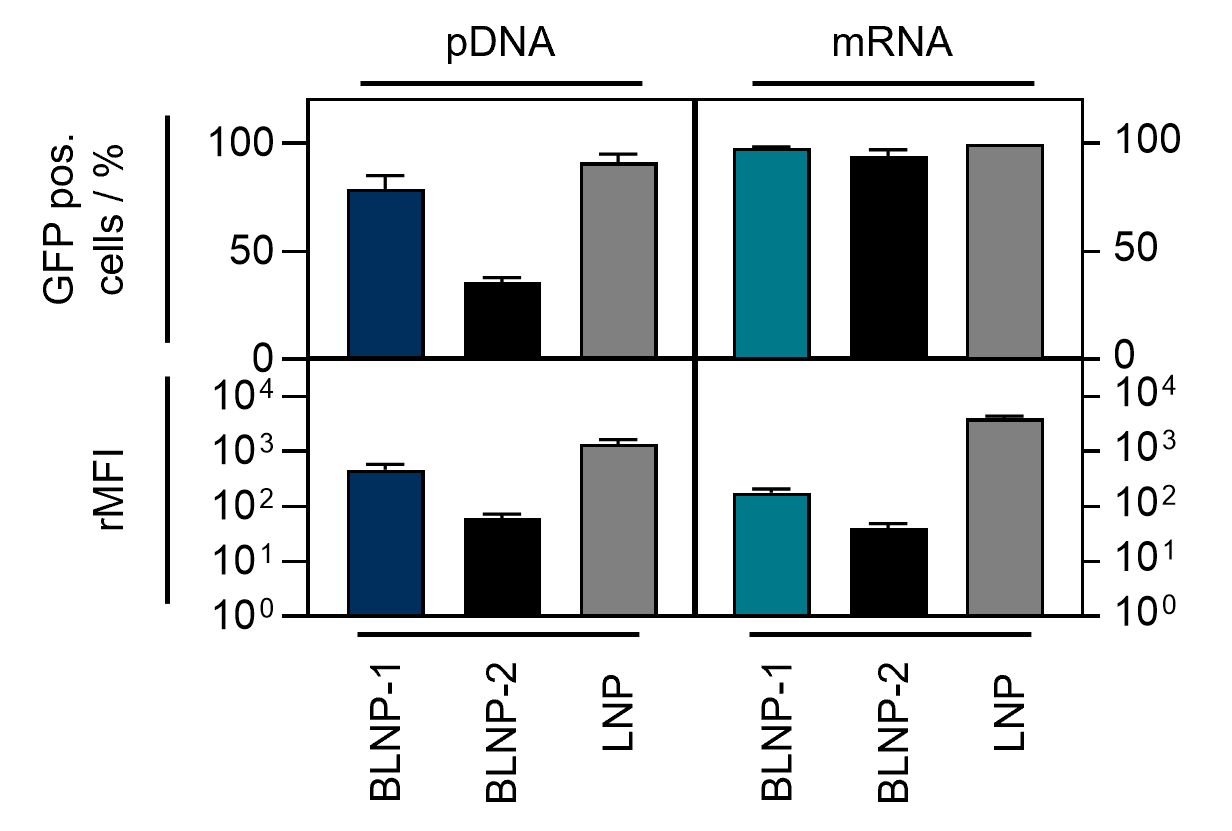


**Figure S7. Comparison of BLNP and LNP *in vitro*.** Results of transfection of HEK293T cells with pDNA (GFP) and mRNA (GFP) assay after 24 h applying 1.0 µg mL^-1^ nucleic acid analyzed *via* flow cytometry. Data shown as mean + s.d. (n = 3 biological replicates).


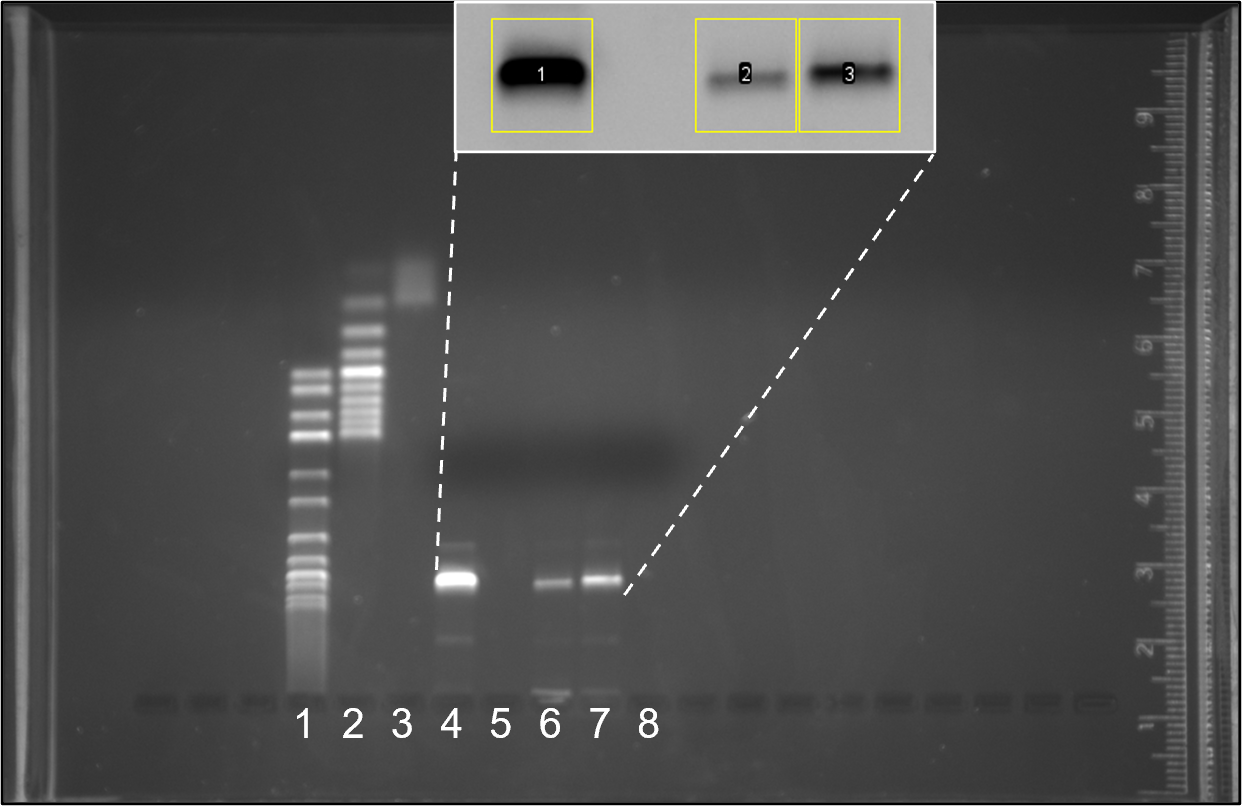


**Figure S8. Gel migration assay to determine free pDNA. A** 1% Agarose gel, supplemented with ethidium bromide for pDNA detection: **1** High Range Ladder, **2** 100 bp ladder, **3** 20 bp ladder, **4** pDNA (GFP) reference, **5** BLNP-1 (PFN) **6** BLNP-1, pDNA (GFP), **7** BLNP-2, pDNA (GFP), **8** BLNP-2 (PFN). Cornered signals were used for quantification of free nucleic acid with ImageJ V1.53t. Quantitative values are provided in Tab. S2.

**Table S2. Quantification of gel migration assay with pDNA.**

|  | **BLNP-1 (pDNA)** | **BLNP-2 (pDNA)** |
| --- | --- | --- |
| **Encapsulation / %** | 80.3 | 59.0 |

Analyzed gel image shown in Fig. S8.


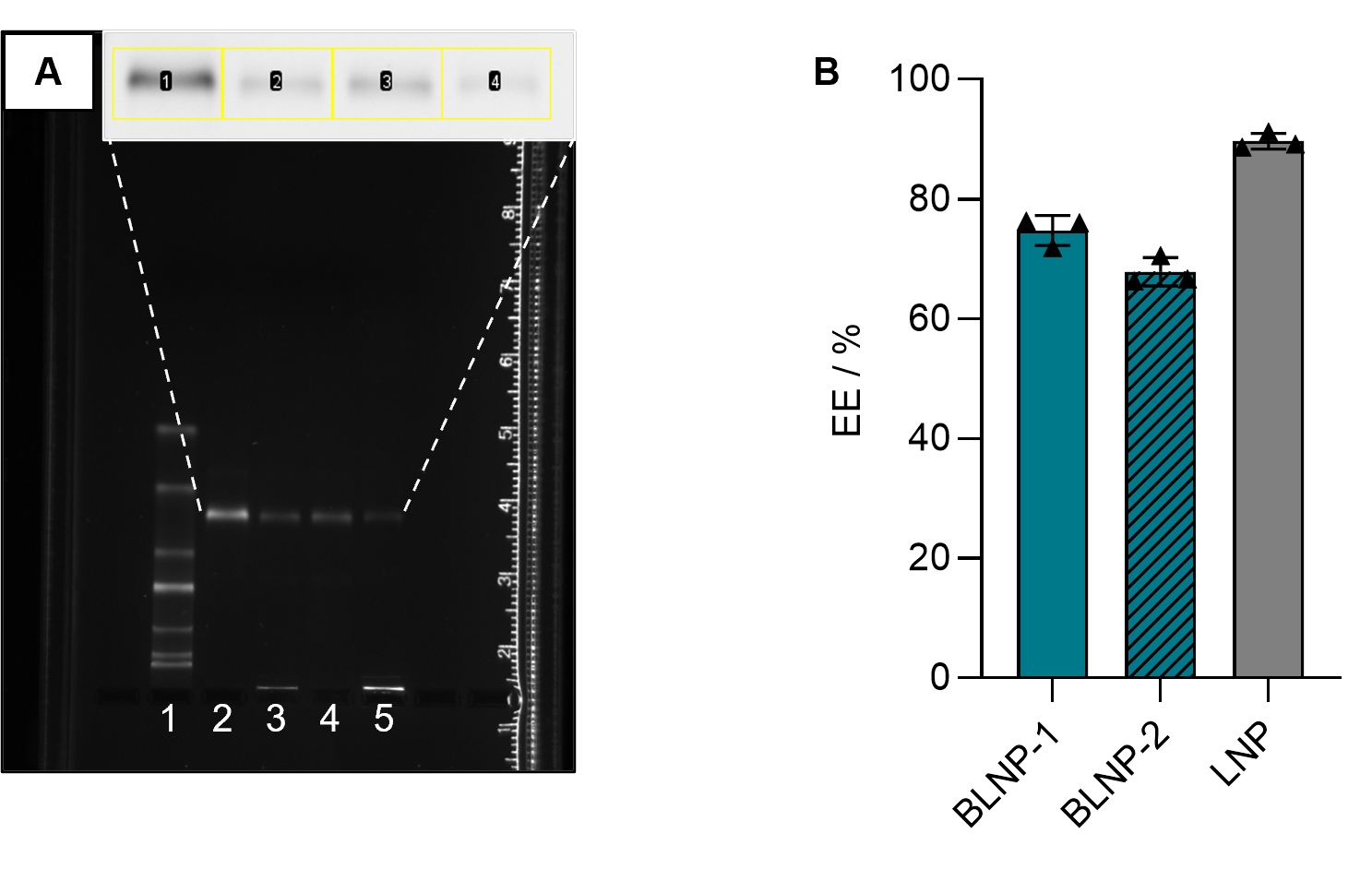


**Figure S9. Gel migration assay to determine free mRNA. A** Representative 2% (w/v) agarose gel, supplemented with SYBR Green II RNA gel stain for mRNA detection: **1** ssRNA Ladder, **2** mRNA (CRE) reference, **3** BLNP-1, mRNA (CRE), **4** BLNP-2, mRNA (CRE). **5** LNP, mRNA (CRE). BLNP-1 revealed less free nucleic acid compared to BLNP-2. Nucleic acid is detected in the sample pocket for BLNP and LNP. Cornered signals were used for quantification of free nucleic acid with ImageJ V1.53t. **B** The encapsulation efficiency (EE) was quantitatively analyzed using the gel assay described in A, which was evaluated with ImageJ V1.53t. Data shown as mean ± s.d., individual data points applied (n = 3 replicates).


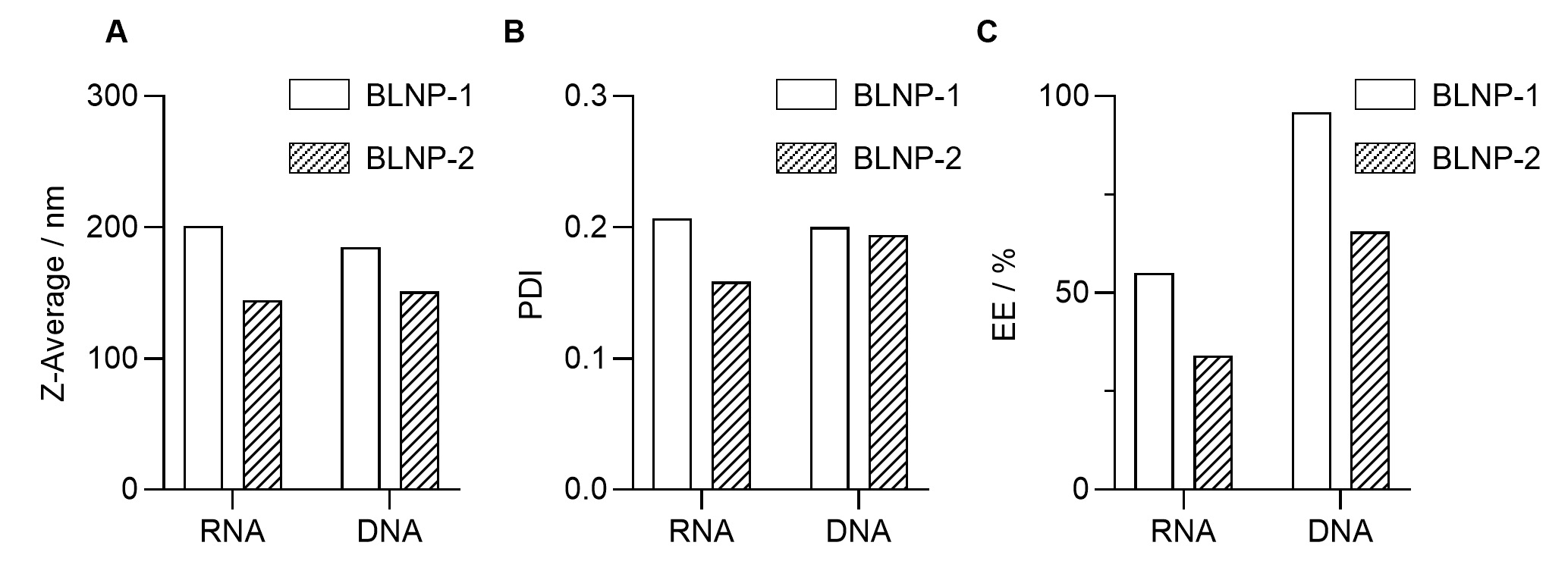


**Figure S10. Microfluidic formulation of nucleic acid-loaded BLNPs. A, B** DLS characterization of BLNP-1 and BLNP-2 encapsulating mRNA and pDNA, respectively. **C** Determination of encapsulation efficiency by gel migration assay. Data shown as a single replicate (n = 1).


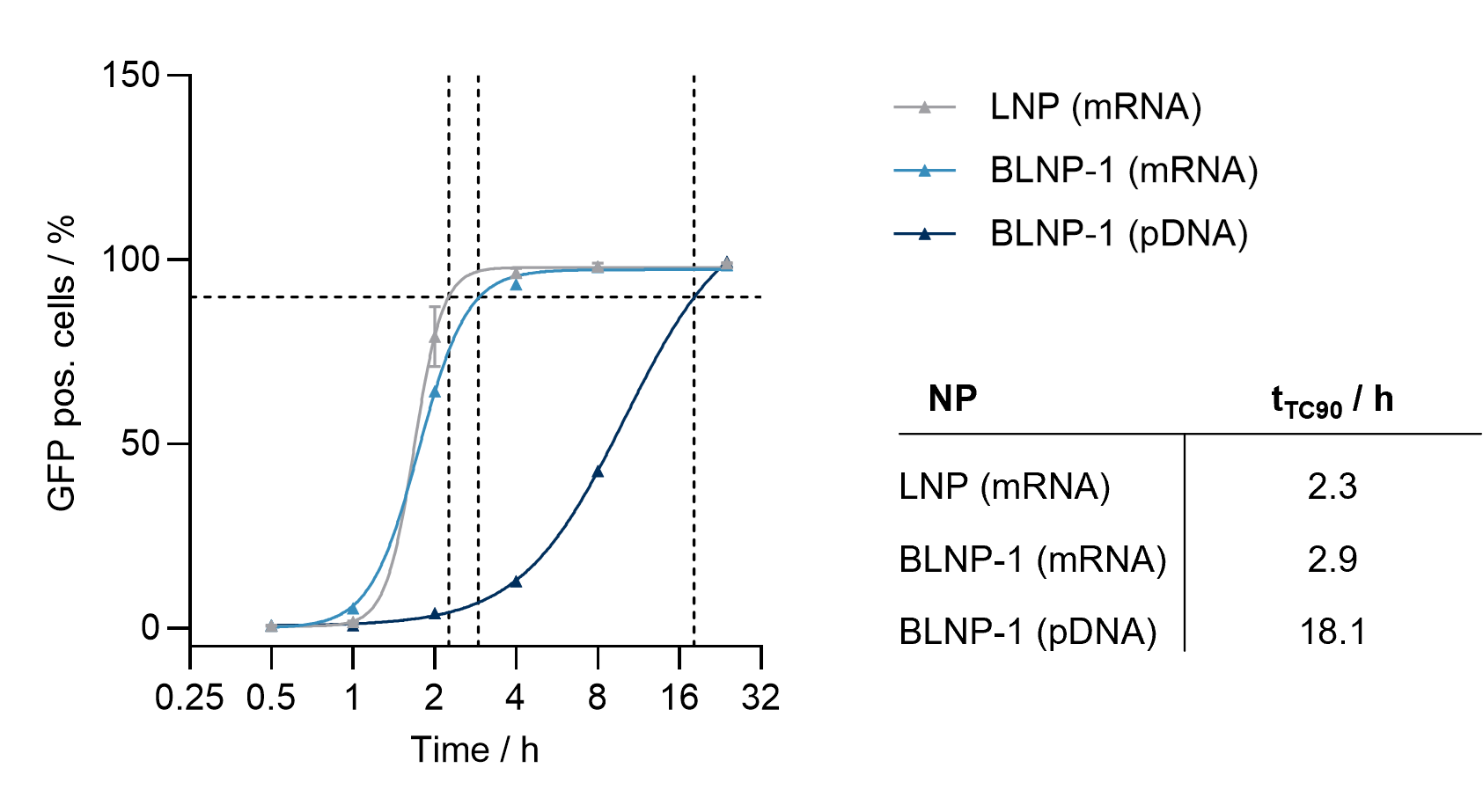


**Figure S11. *In vitro* transfection kinetics study.** Time-dependent transfection of HEK293T cells using 3.0 µg mL^-1^ nucleic acid. The data demonstrate that mRNA leads to faster protein expression, as indicated by the time to reach 90% transfected cells (TC_90_). For BLNP-1 loaded with mRNA, TC_90_ was 2.9 h, whereas BLNP-1 loaded with pDNA reached TC_90_ at 18.1 h. The standard LNP control exhibited slightly faster kinetics, with a TC_90_ of 2.3 h. Dotted lines mark TC_90_ values. BLNP data shown as single biological replicate. LNP (mRNA) (molar fraction of 46.3 mol% ALC-0315, 9.4 mol% DSPC, 42.7 mol% cholesterol, and 1.6 mol% of PEG-lipid) data was previously published by C. T. Holick, *et al*.[4] and was added for comparison reasons. Data acquisition and processing were conducted using the same methodology as applied to the BLNP formulations and is printed and adapted with permission from the publisher and corresponding author (https://doi.org/10.1002/smll.202411354, Figure S14, license: https://creativecommons.org/licenses/by/4.0/).


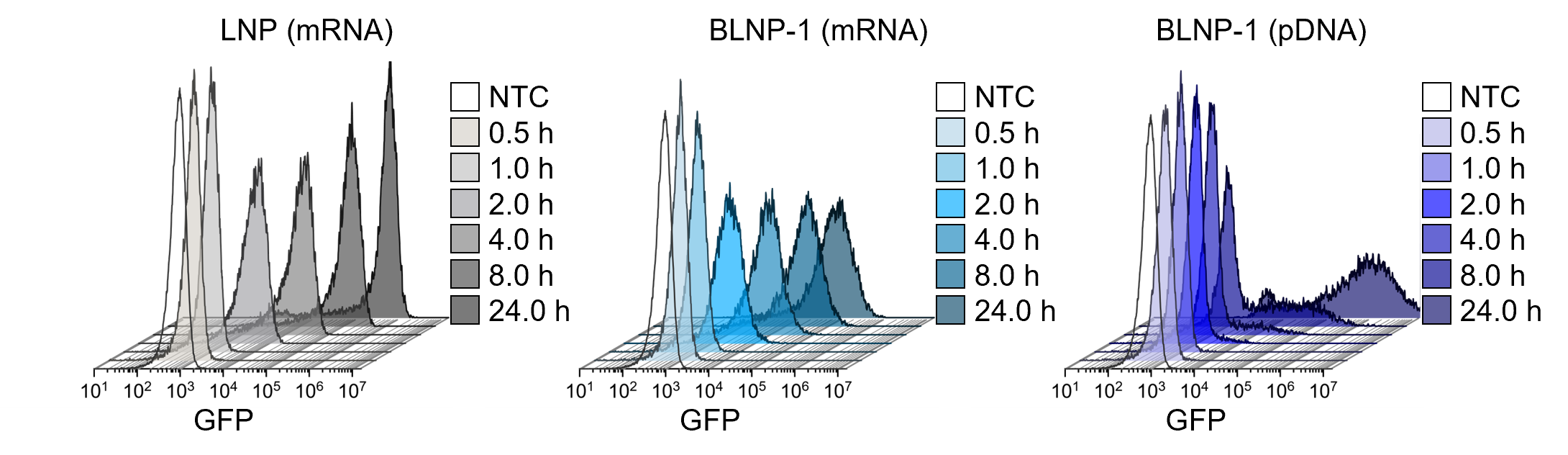


**Figure S12. Histograms of *in vitro* transfection kinetics study.** Normalized histograms of GFP expression levels analyzed by flow cytometry. Data are shown for one replicate consisting of 10,000 single cells. LNP (mRNA) (molar fraction of 46.3 mol% ALC-0315, 9.4 mol% DSPC, 42.7 mol% cholesterol, and 1.6 mol% of PEG-lipid) data was previously published by C. T. Holick, *et al*.[4] and was added for comparison reasons. Data acquisition and processing were conducted using the same methodology as applied to the BLNP formulations and is printed and adapted with permission from the publisher and corresponding author (https://doi.org/10.1002/smll.202411354, Figure S14, license: https://creativecommons.org/licenses/by/4.0/).


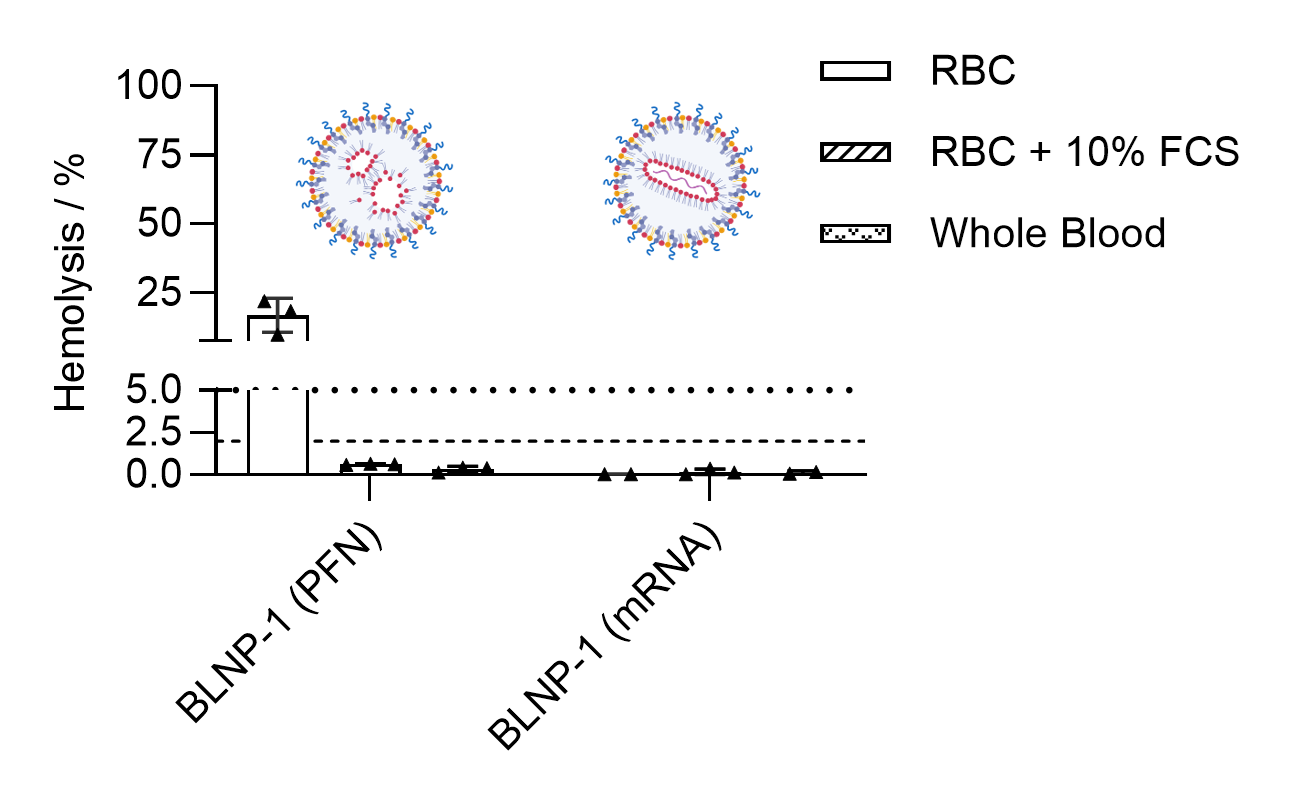


**Figure S13. Hemolysis assay in isolated RBCs and in the presence of serum.** A concentration of ≈ 130 µM total lipid (equivalent to 3.0 µg mL^-1^ mRNA, BLNP-1) and a maximal exposure condition of 1000 µM total lipid as PFNs without mRNA (BLNP-1 PFN) were tested. The hemolysis assay was adapted to approximate physiological conditions: blood samples were divided, with erythrocytes (RBCs) isolated from one fraction and measured, while a second fraction of isolated RBCs was supplemented with 10% FCS. Whole blood was additionally analyzed without erythrocyte isolation and showed no hemolytic effect in the presence of serum, most likely due to plasma protein binding. Data shown as mean ± s.d. (n = 3 biological replicates, individual data points applied).


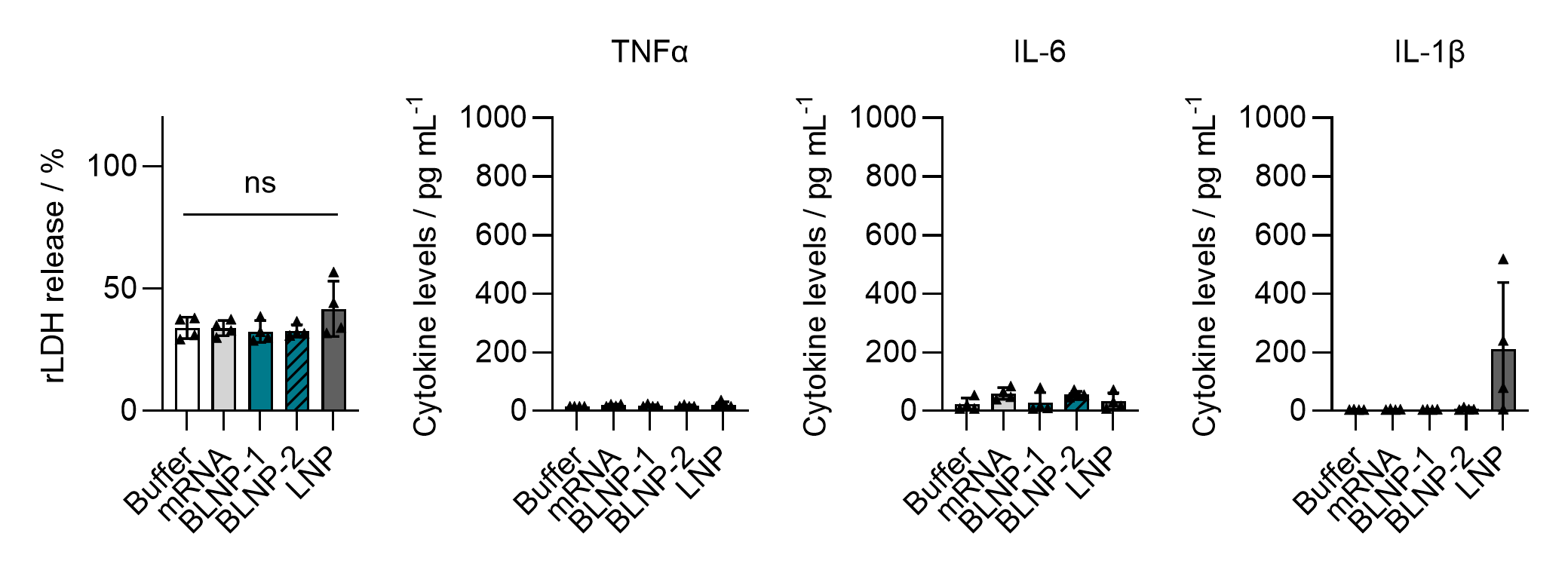


**Figure S14. Screening for cytotoxicity on primary human cells and immunomodulatory properties.** BLNP and LNP were applied to human peripheral blood mononuclear cells (PBMC) and polymorphonuclear leukocytes (PMNL) *ex vivo*, and cytotoxicity was investigated via LDH assay after 3 h of incubation. Additionally, the cytokine profile was determined via ELISA after 18 h of incubation. No significant cytotoxicity was observed for the nanoparticles compared to buffer alone or free mRNA. No relevant cytokine release was observed. Data shown as mean ± s.d. (n = 4 biological replicates; individual data points applied). ns p > 0.05 derived from a two-way analysis of variance (ANOVA).


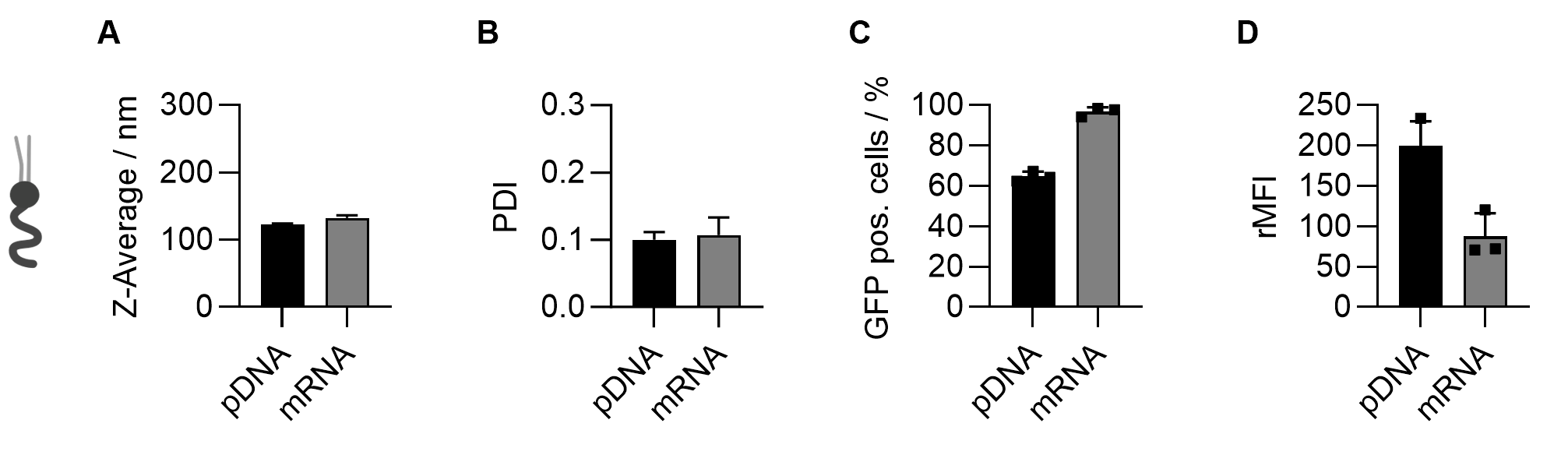


**Figure S15. Investigations of BLNP-2 formulation.** **A, B** DLS measurements (Z‑Average, PDI) of BLNP-2 loaded with pDNA (GFP) or mRNA (GFP). **C, D**Transfection investigations in HEK293T cells (GFP pos. cells, rMFI) of BLNP-2 loaded with pDNA (GFP) or mRNA (GFP). Data shown as mean + s.d. (A, B n = 3 replicates; C, D 3 biological replicates, individual data points applied).


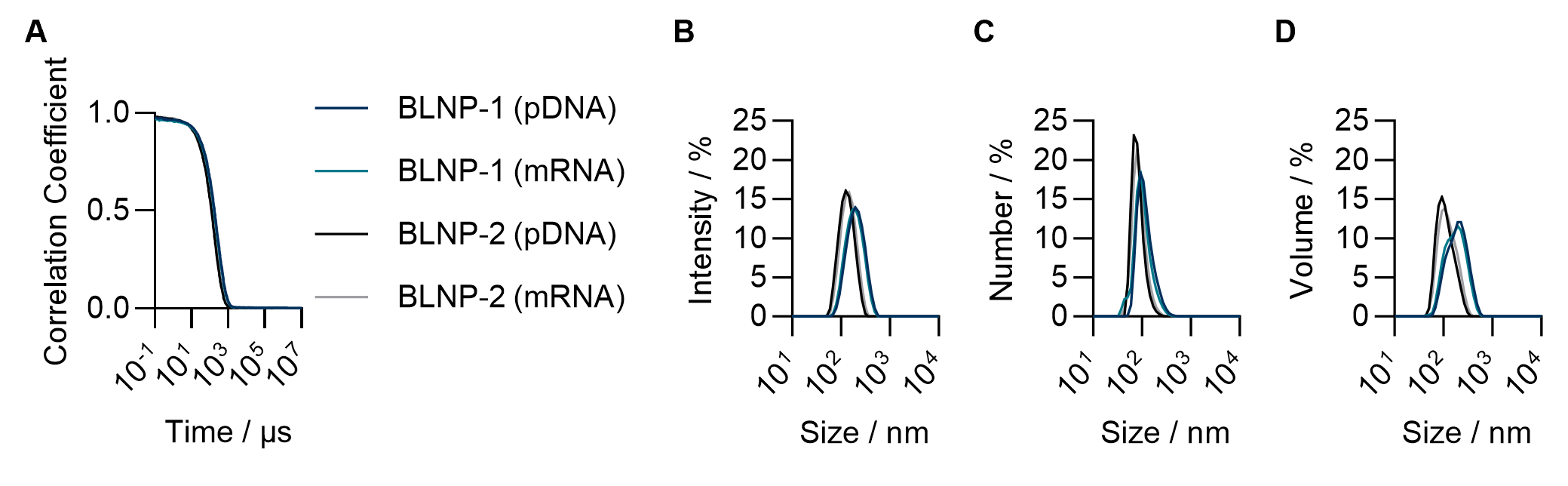


**Figure S16. Correlation coefficient and size distribution of BLNP.** **A-D** Correlation coefficient and size distribution by intensity, number, and volume of BLNP-1 encapsulating pDNA (GFP) or mRNA (GFP) and BLNP-2 encapsulating pDNA (GFP) or mRNA (GFP). Data shown as mean (n = 3 replicates).

**
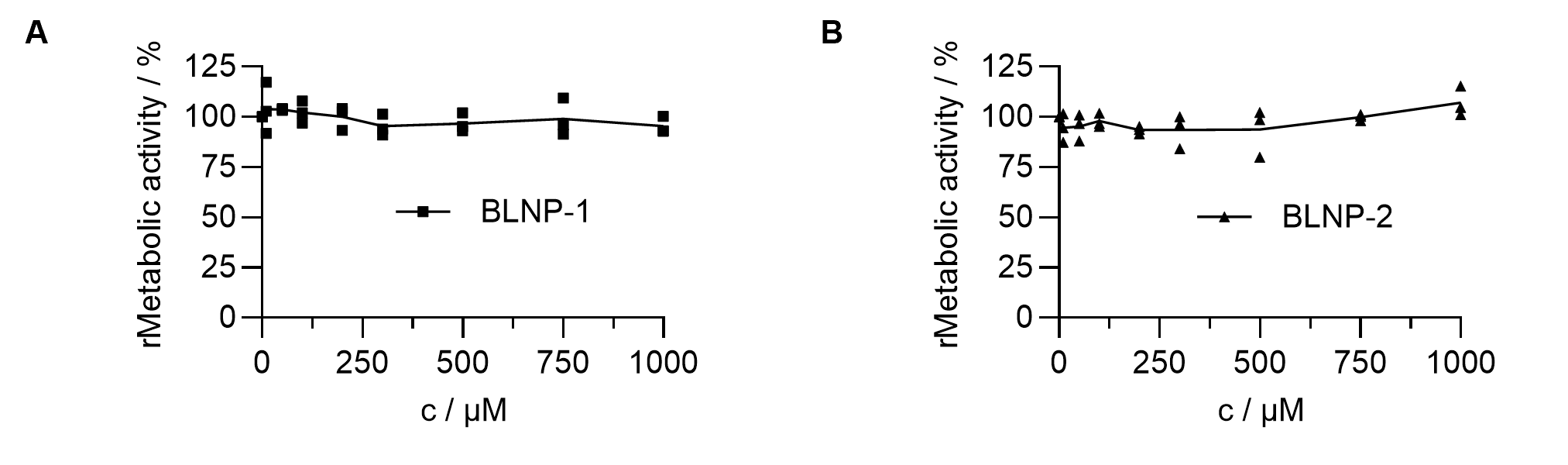
**

**Figure S17. PrestoBlue assay to determine the cytotoxicity of BLNP in L929 cells.** PrestoBlue assay analyzed after 24 h of incubation. **A** BLNP-1, **B** BLNP-2. The concentration corresponds to the total lipid amount. Fluorescence is shown relative to buffer control. Data shown as mean (connecting line) and single data points (n = 3 biological replicates).


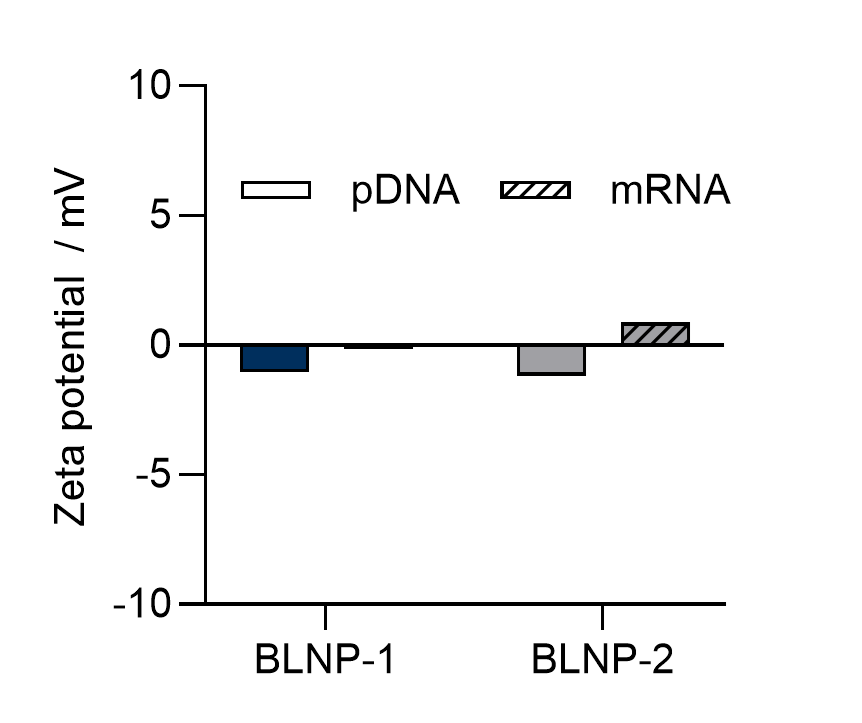


**Figure S18. Zeta potential measurement of BLNP.** BLNP-1 and BLNP-2 loaded with pDNA (GFP) and mRNA (GFP). Nanoparticles were diluted 1 + 9 prior to the measurement in PBS buffer (pH 7.4). Data shown as mean (n = 3 technical replicates).


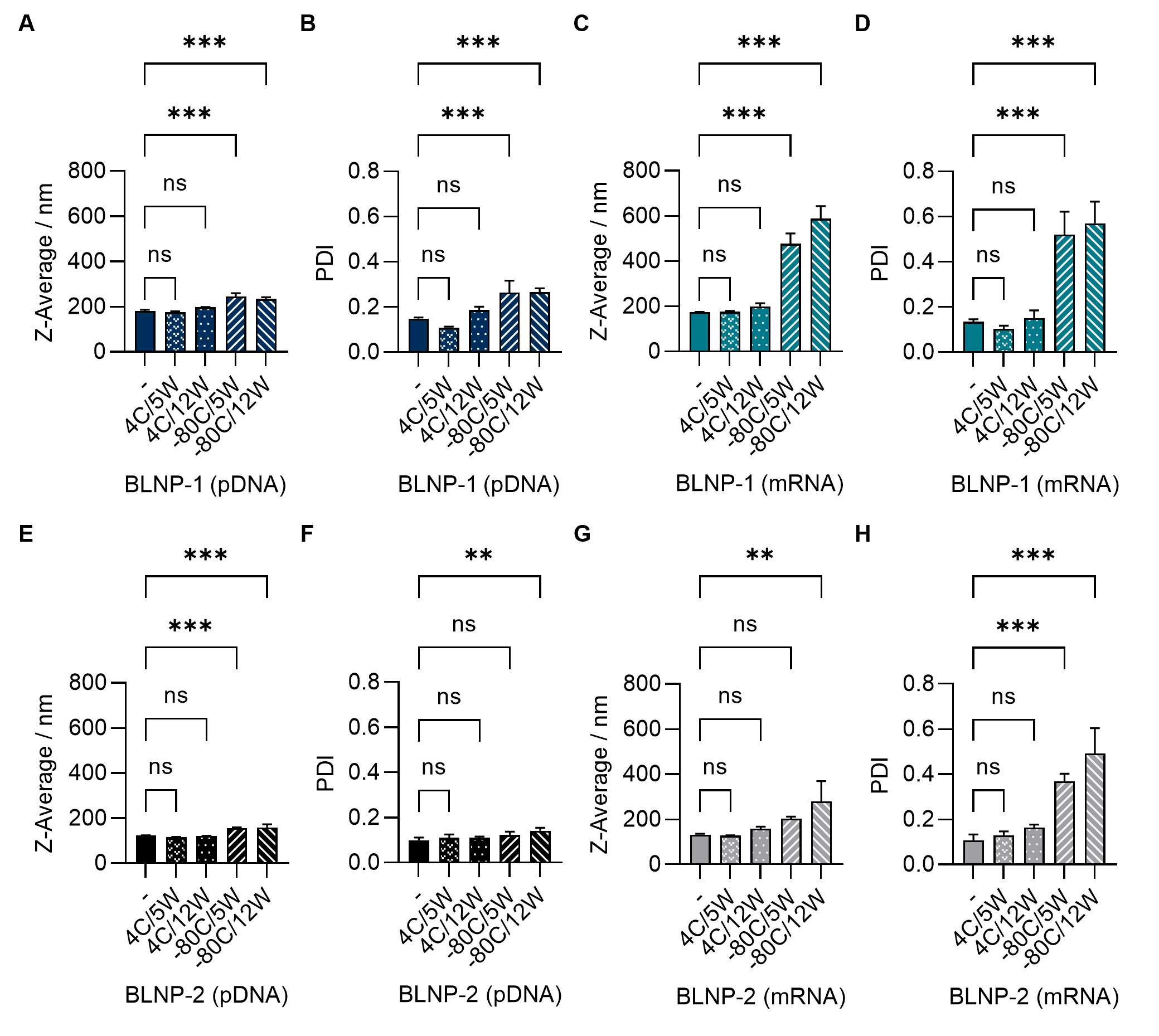


**Figure S19. Storage stability of BLNP-1 and BLNP-2. A, E** Z-Average of BLNP-1 and BLNP-2 loaded with pDNA (GFP) after 5 and 12 weeks of storage at 4 °C and –80 °C. **B, F** PDI of BLNP-1 and BLNP-2, pDNA (GFP) after 5 and 12 weeks of storage at 4 °C and –80 °C. **C, G** Z-Average of BLNP-1 and BLNP-2, mRNA (GFP) after 5 and 12 weeks of storage at 4 °C and –80 °C. **D, H** PDI of BLNP-1 and BLNP-2, mRNA (GFP) after 5 and 12 weeks of storage at 4 °C and –80 °C. No significant change in size was measured after storing at 4 °C. Data shown as mean + s.d. (n = 3 replicates). *** p ≤ 0.001 derived from a one-way analysis of variance (ANOVA).


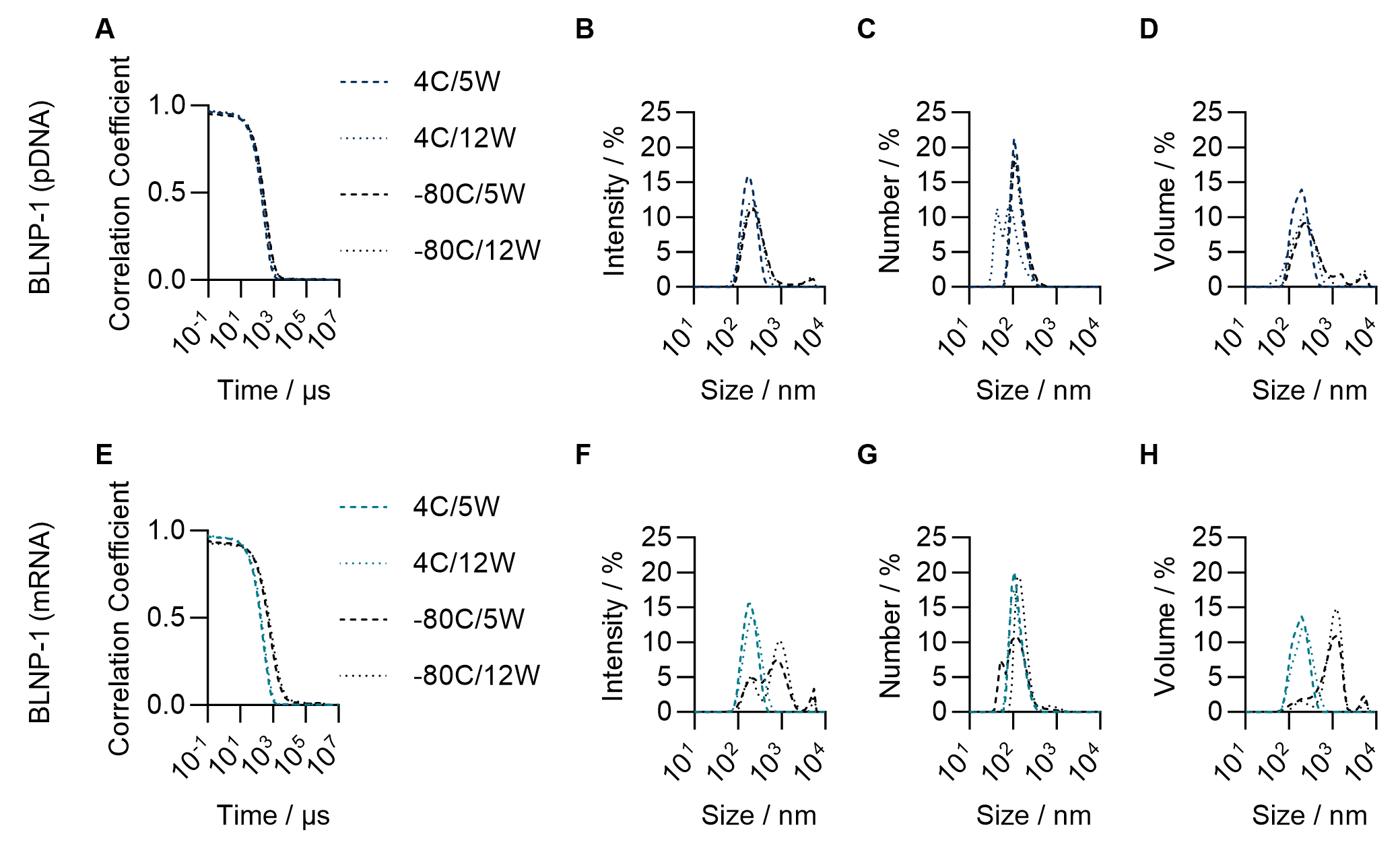


**Figure S20. Correlation coefficient and size distribution of BLNP-1 after storage. A‑D** Correlation Coefficient and size distribution by intensity, number, and volume of BLNP-1, pDNA (GFP) at different timepoints, stored at 4 °C and –80 °C. **E-H** Correlation Coefficient and size distribution by intensity, number, and volume of BLNP-1, mRNA (GFP) at different timepoints, stored at 4 °C and –80°C. Data shown as mean (n = 3 replicates).


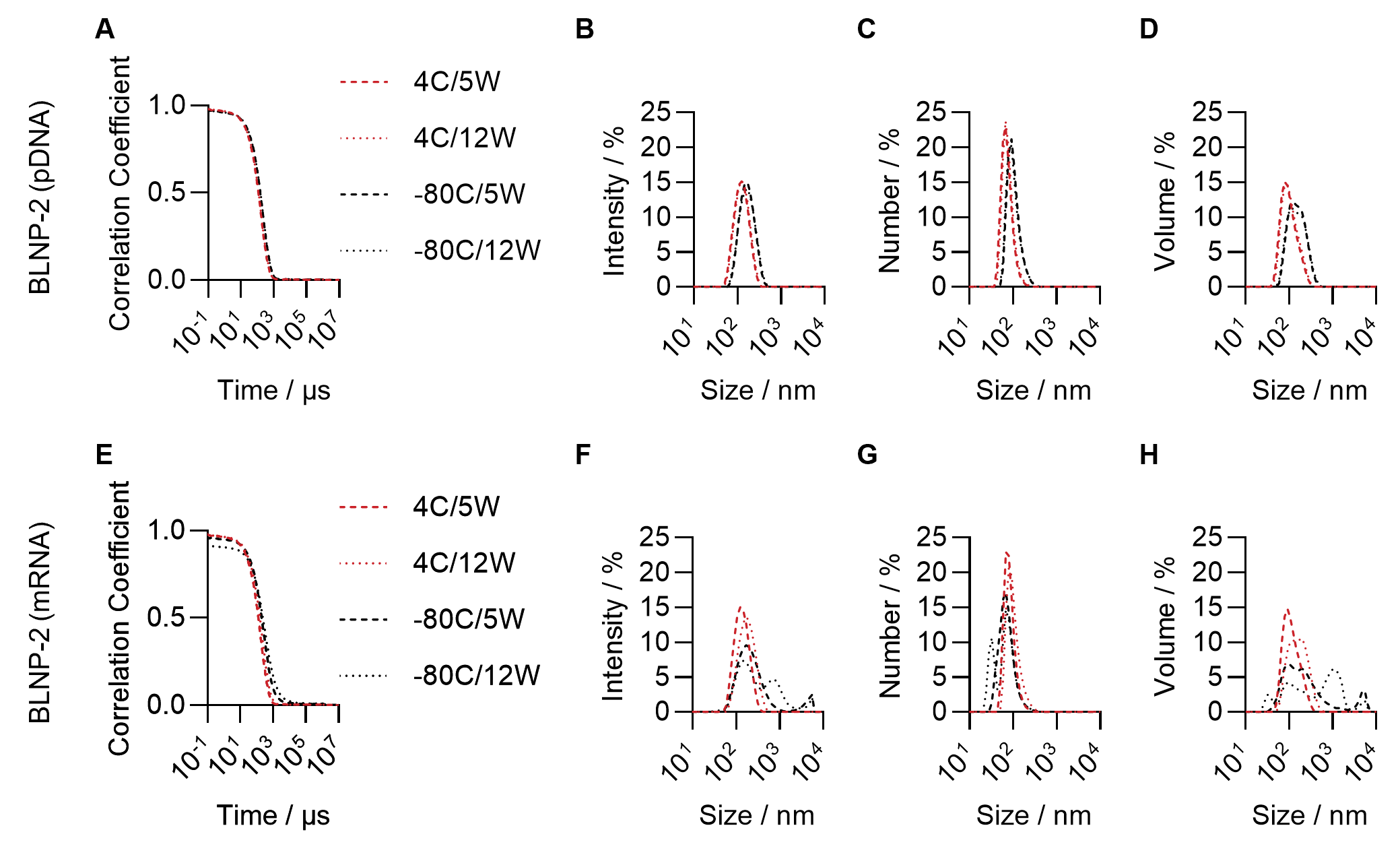


**Figure S21. Correlation coefficient and size distribution of BLNP-2 after storage. A-D** Correlation coefficient and size distribution by intensity, number, and volume of BLNP-2, pDNA (GFP) at different timepoints, stored at 4 °C and –80 °C. **E-H** Correlation coefficient and size distribution by intensity, number, and volume of BLNP-2, mRNA (GFP) at different timepoints, stored at 4 °C and –80°C. Data shown as mean (n = 3 replicates).


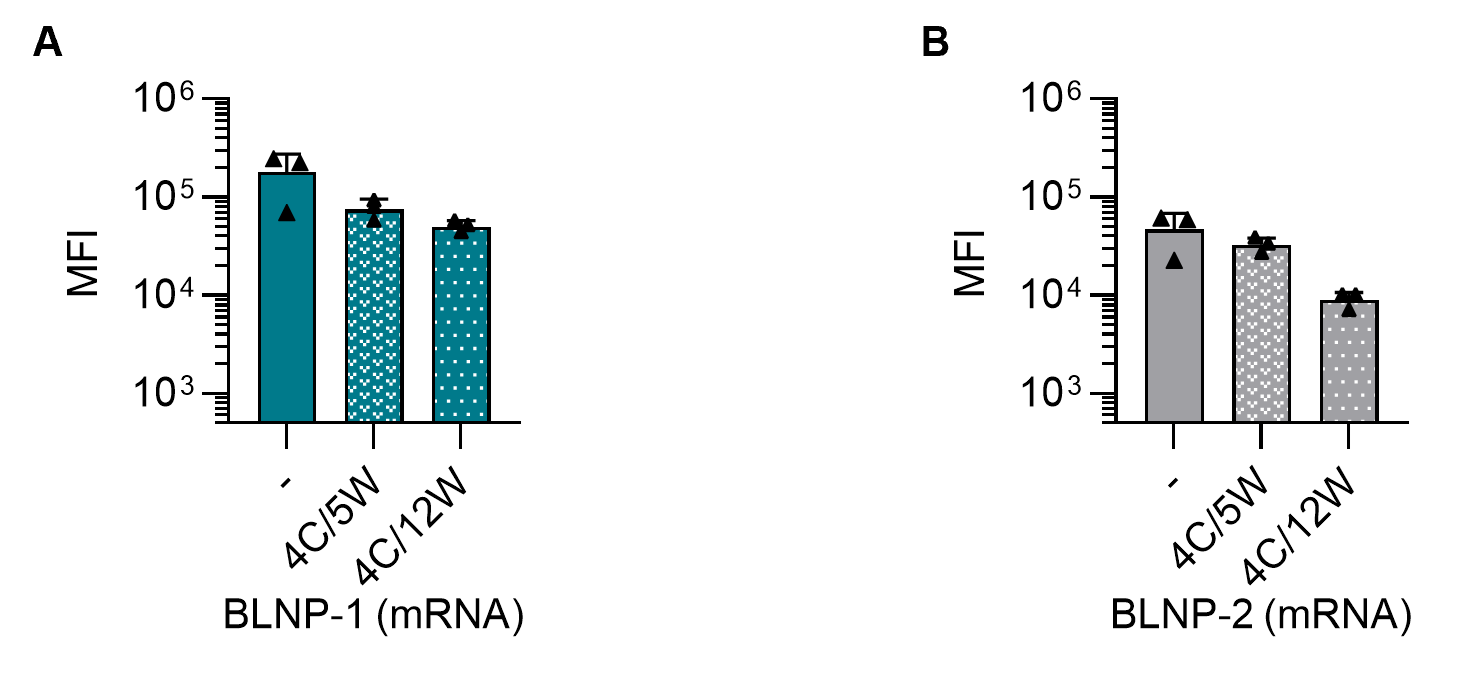


**Figure S22. Transfection after storage at 4 °C.** **A** BLNP-1 were stored at 4 °C for 5 and 12 weeks and used for transfection of HEK293T cells. **B** BLNP-2 were stored at 4 °C for 5 and 12 weeks and used for transfection of HEK293T cells. Data shown as mean ± s.d. (n = 3 biological replicates, individual data points applied)


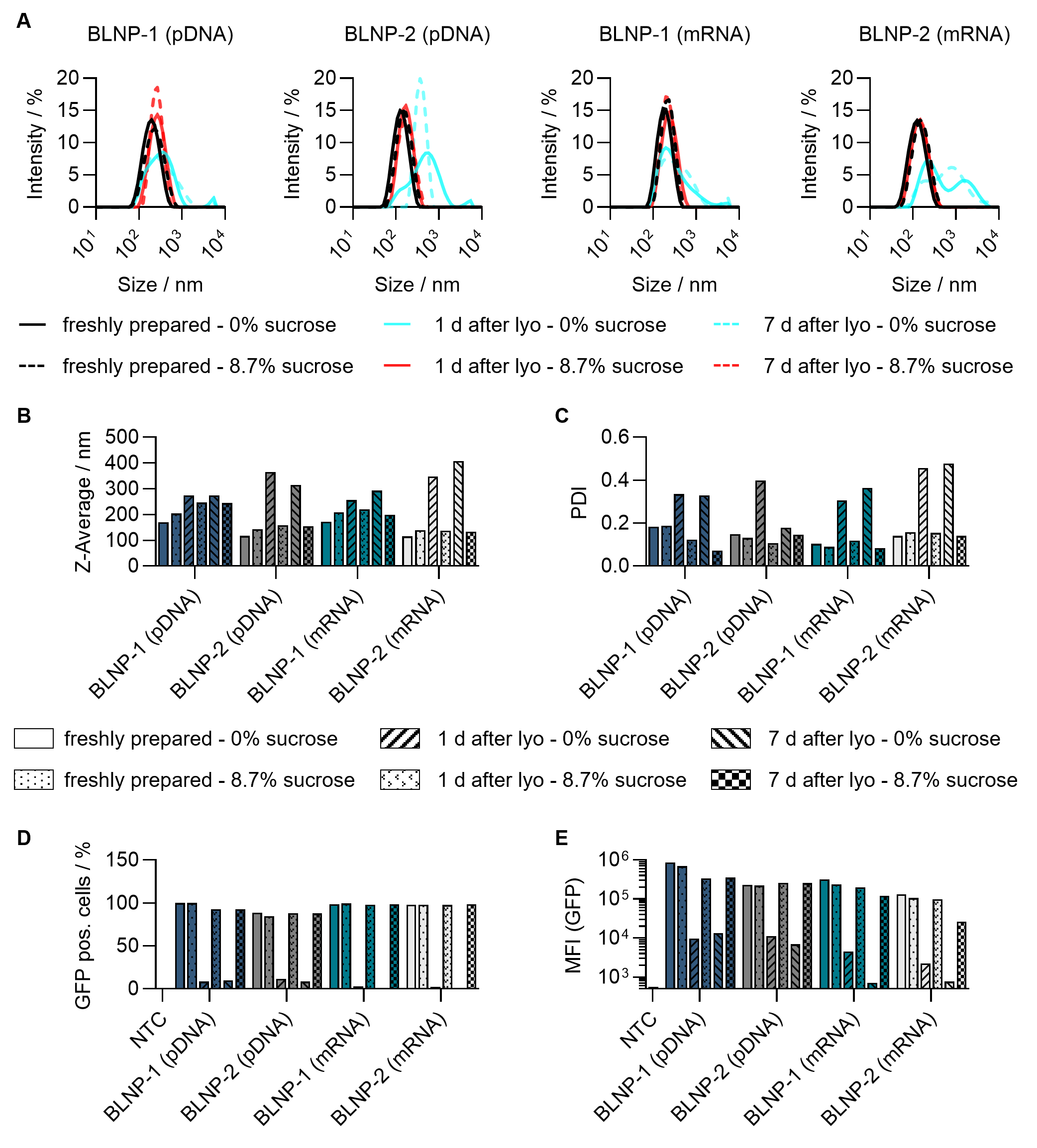
**Figure S23. Stability assessment of BLNP formulations after lyophilization.** BLNP-1 and BLNP-2 were freeze-dried in the presence or absence of cryoprotectant (8.7% sucrose) and stored at 4 °C for one day. Freshly prepared samples were used as controls and measured by DLS prior to the freeze-drying procedure. **A-C** After reconstitution in PBS, **A** DLS intensity distribution curves, **B** Z-Average diameter, and **C** PDI were assessed. **D, E** Transfection efficiency was evaluated in HEK293T cells using 3.0 µg mL^-1^ nucleic acid. The results demonstrate that formulations lyophilized with sucrose retained favorable physicochemical properties and biological activity. Data presented as single replicate (n = 1).


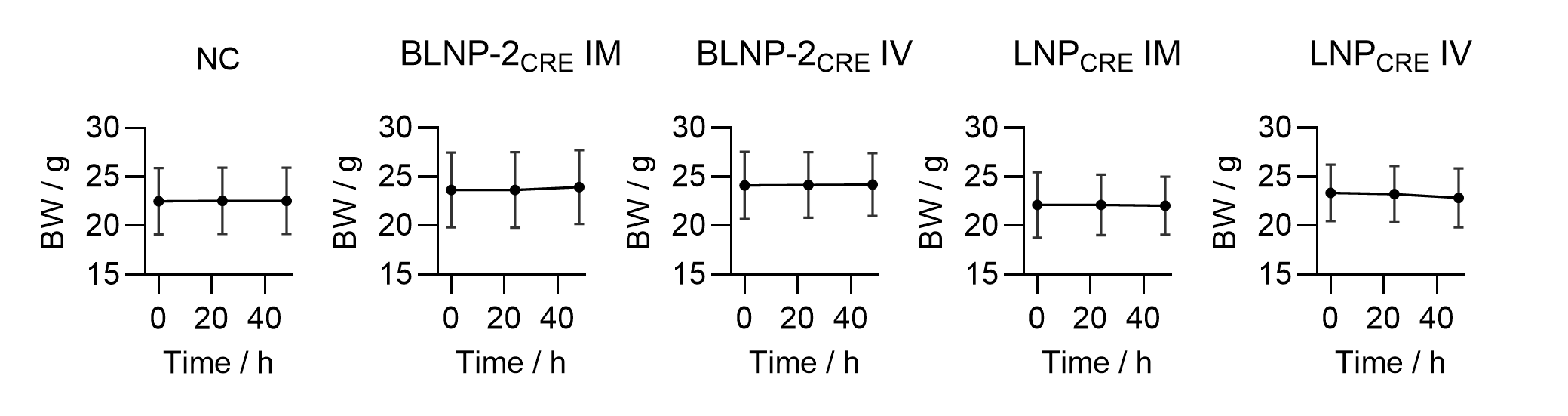


**Figure S24. Monitoring animal body weight (BW) during *in vivo* studies.** Body weight was assessed before treatment (0 h), 24 h after treatment, and 48 h after treatment, before the animals were sacrificed. Non-treated animals (NC) (n = 6) were compared with BLNP-2_CRE_ intramuscularly (IM) (n = 4), BLNP-2_CRE_ intravenously (IV) (n = 3), LNP_CRE_ IM (n = 4) and LNP_CRE_ IV (n = 4) treated animals. Data shown as mean ± s.d. No significant differences were derived from a two-way analysis of variance (ANOVA).


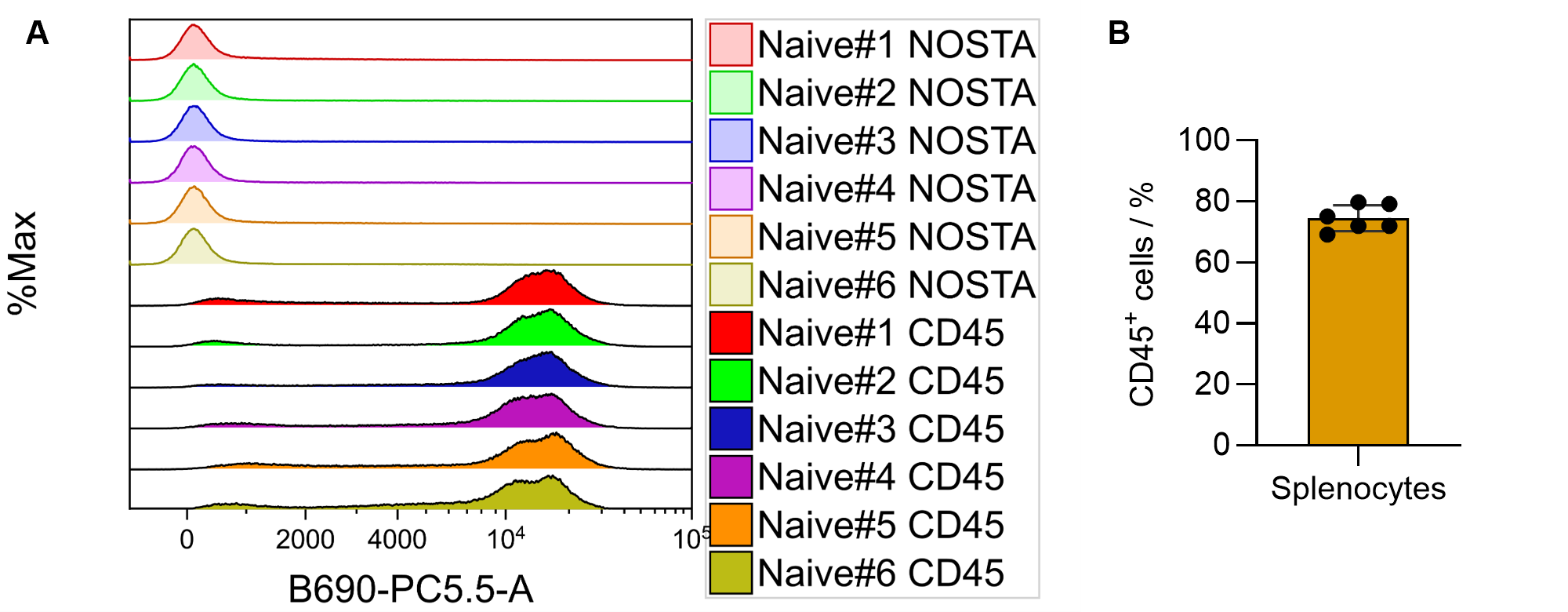


**Figure S25. Identification of CD45^+^splenocytes from the Ai9/Cre mouse model.** **A** Splenocytes were stained with PerCP-CD45 (CD45) antibodies and measured applying a 488 nm laser with a 690/50 nm bandpass filter. Histograms for six non treated (naive) mice were shown and non-stained (NOSTA) samples were compared with CD45 stained samples for each animal. **B** CD45^+^ cells were analyzed for each naive animal (n = 6, data shown as mean ± s.d., individual data points applied).


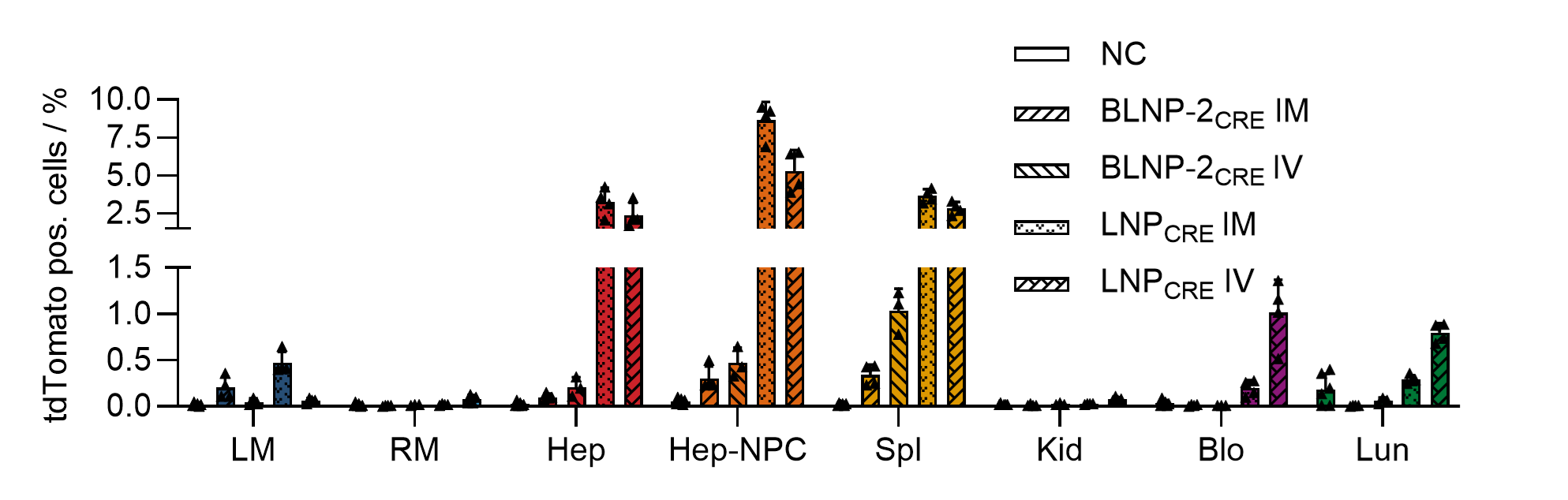


**Figure S26. Flow cytometry analysis of different tissues from the Ai9/Cre mouse model.** tdTomato pos. single cells in various tissues. Non-treated animals (NC) (n = 6) were compared with BLNP-2_CRE_ intramuscularly (IM) (n = 4), BLNP-2_CRE_ intravenously (IV) (n = 3), LNP_CRE_ IM (n = 4) and LNP_CRE_ IV (n = 4) treated animals. Data shown as mean + s.d. (individual data points applied). Gating according to Figure S31.

**
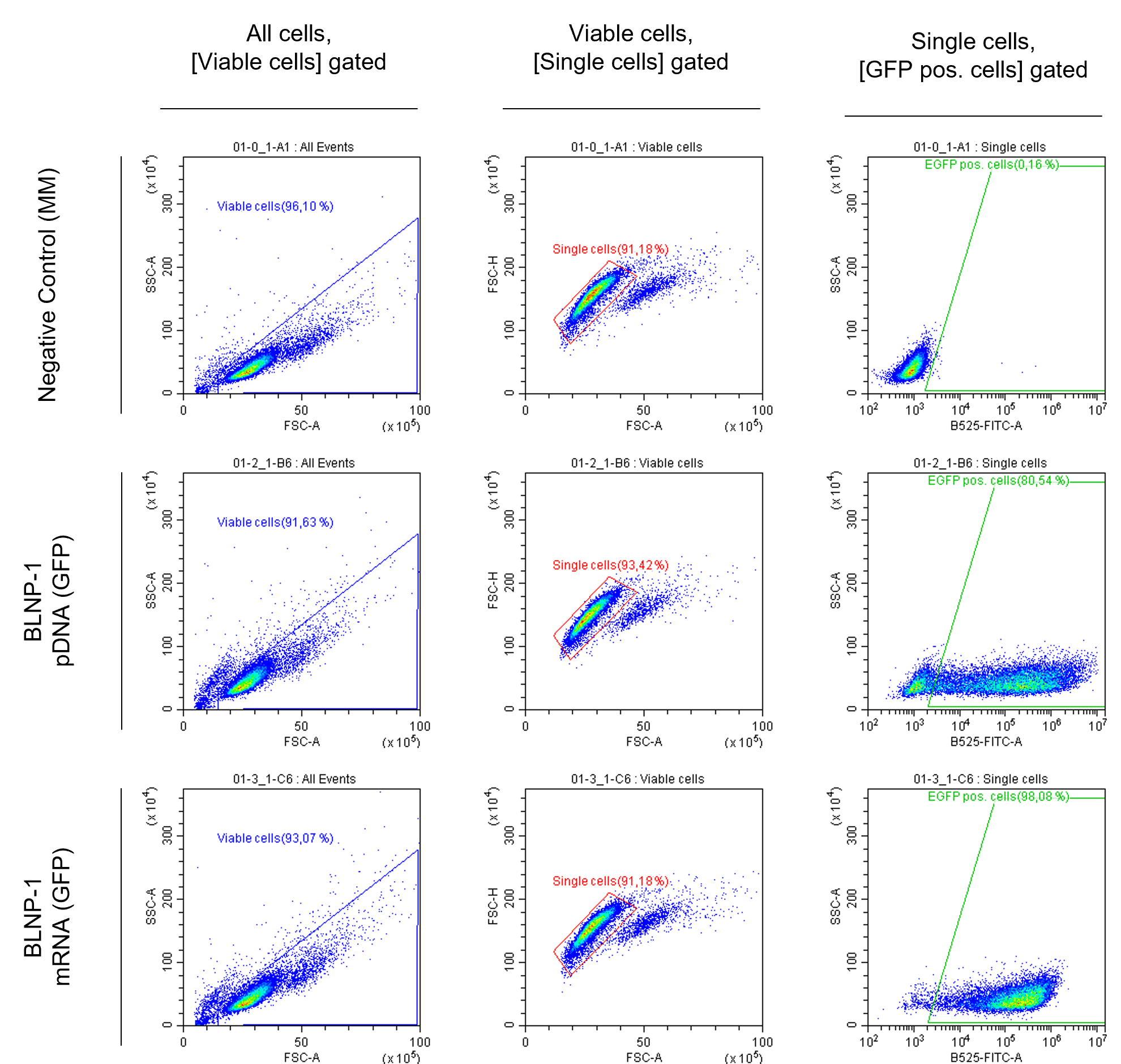
**

**Figure S27. Gating strategy: transfection of HEK293T cells with pDNA (GFP) and mRNA (GFP).** Viable single cells were analyzed by forward and sideward scatter (FSC/SSC). Fluorescence was measured at λEx = 488 nm with a 525/40 nm bandpass filter (FITC channel). Positive cells were identified by gating against the cells treated with only nucleic acid (MM). The analysis was conducted using CytExpert V 2.5.0.77.


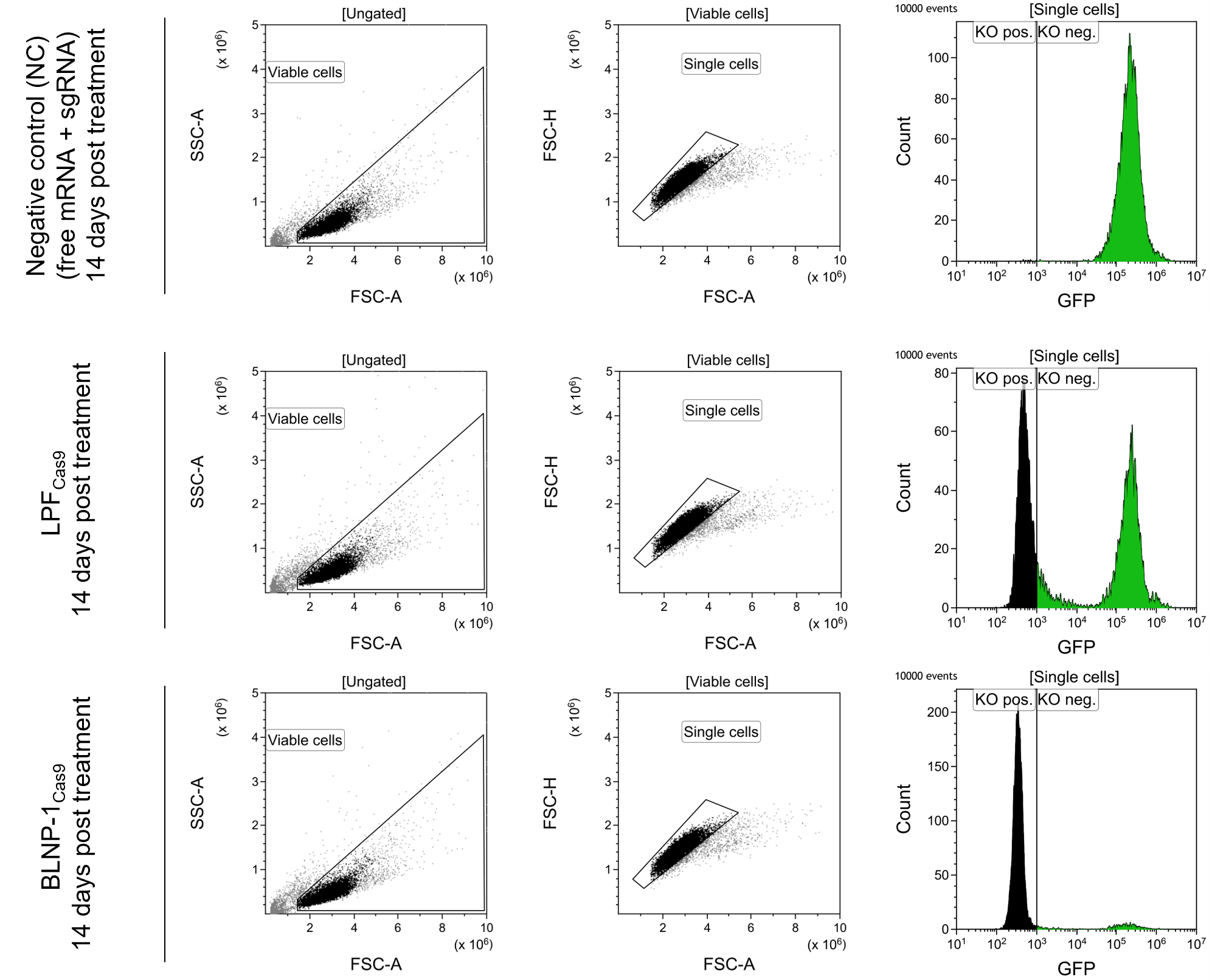


**Figure S28. Gating strategy: GFP knockout in HEK293-GFP stable cells using CRISPR-Cas9.** Viable single cells were analyzed by forward and sideward scatter (FSC/SSC). Fluorescence was measured at λEx = 488 nm with a 510/20 nm bandpass filter (B510GFPND1 channel) (GFP). The analysis was conducted using Kaluza version 2.2.1.


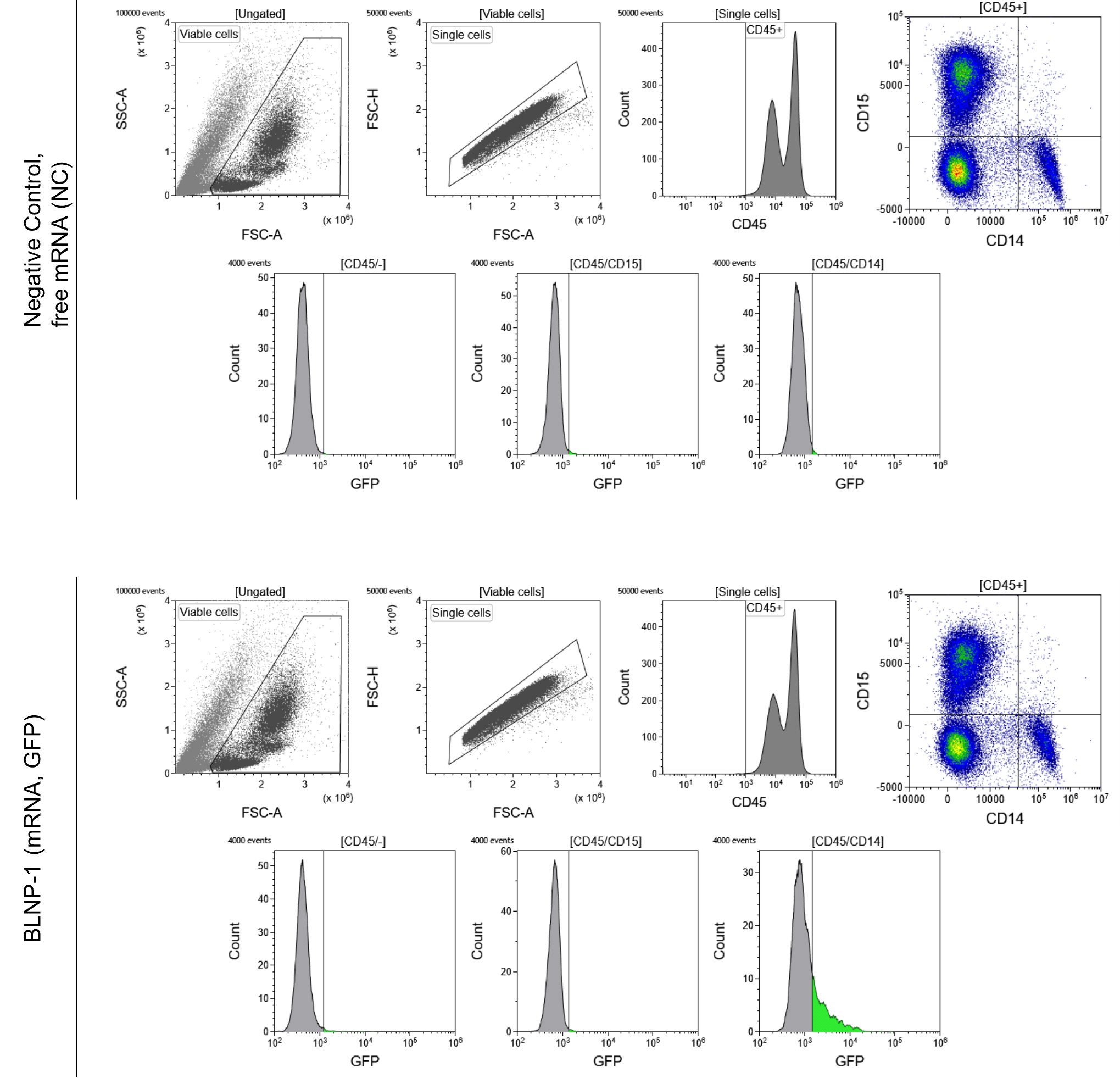


**Figure S29. Gating strategy: Transfection of human PBMC and PMNL with BLNP-1 (mRNA, GFP).** Viable single cells were analyzed by forward and sideward scatter (FSC/SSC). Single CD45^+^ cells were determined by applying a 561 nm laser with a 585/42 nm bandpass filter. CD15^+^ cells were determined by applying a 488 nm laser with a 690/50 nm bandpass filter. CD14^+^ cells were determined by applying a 638 nm laser with a 660/10 nm bandpass filter. GFP pos. cells were determined by applying a 488 nm laser with a 510/10 nm bandpass filter. GFP in different cell populations was investigated. The analysis was conducted using Kaluza version 2.2.1.


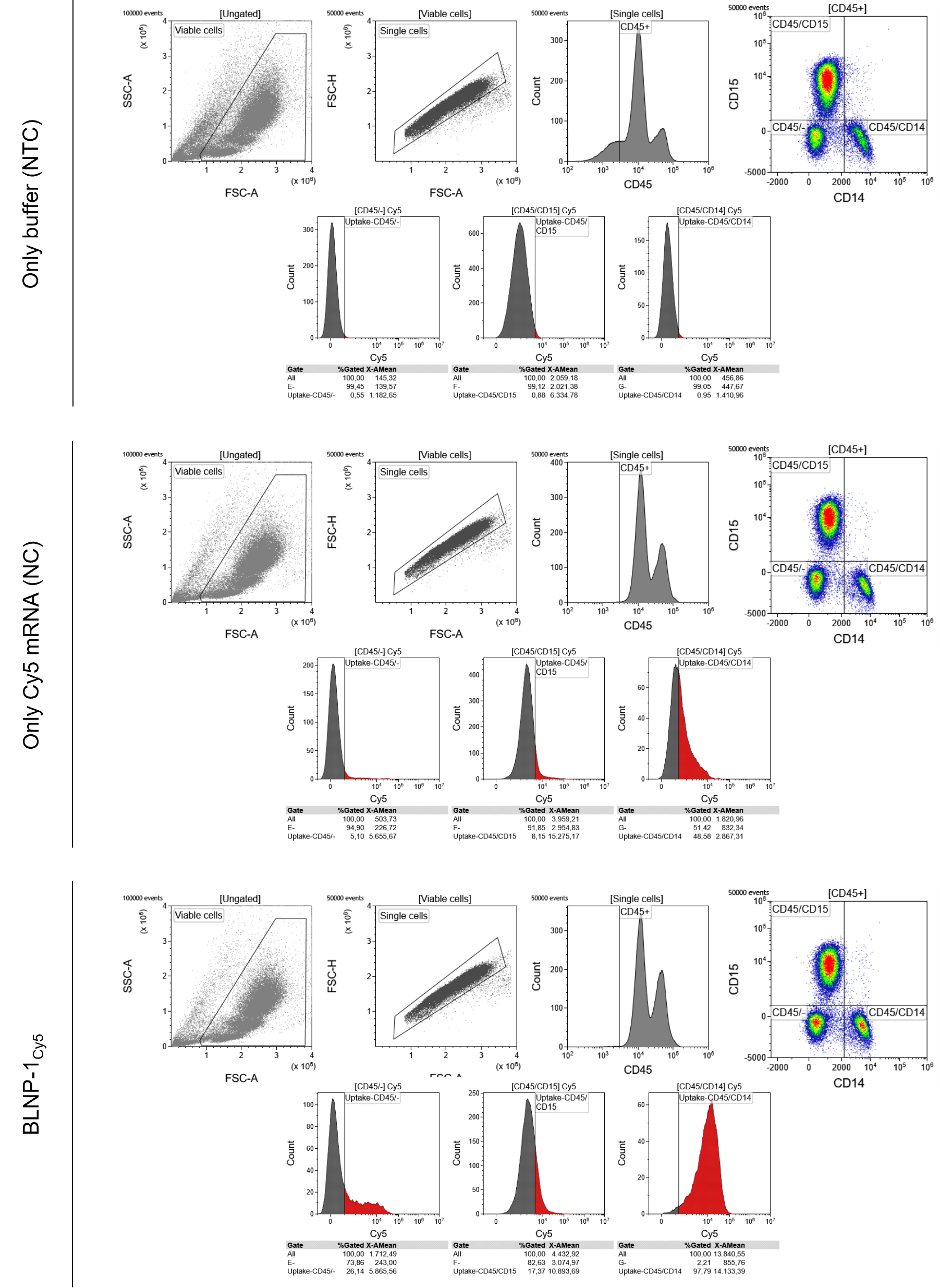


**Figure S30. Gating strategy: Uptake studies in human primary PBMC and PMNL with BLNP‑1_Cy5_.** Viable single cells were analyzed by forward and sideward scatter (FSC/SSC). Single CD45^+^ cells were determined by applying a 561 nm laser was with an 585/42 nm bandpass filter. CD15^+^ cells were determined by applying a 488 nm laser with a 690/50 nm bandpass filter. CD14^+^ cells were determined by applying a 405 nm laser with a 525/40 nm bandpass filter. Cy5 was detected by applying a 638 nm laser using a 660/10 nm bandpass filter. Cy5 in different cell populations was investigated. The analysis was conducted using Kaluza version 2.2.1.


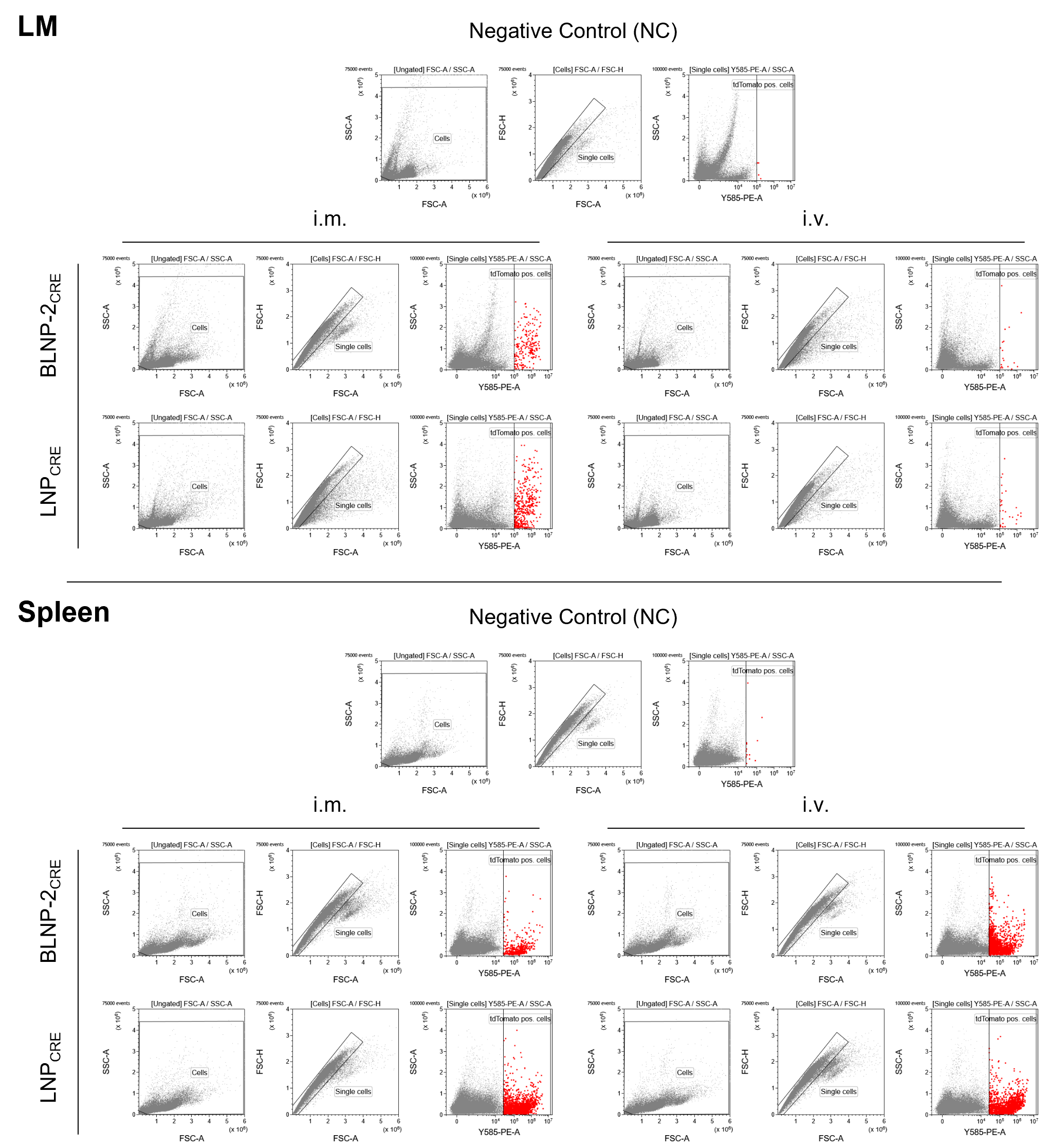


**Figure S31. Gating strategy: *In vivo* transfection of Ai9/Cre reporter mice.** BLNP‑2_CRE_ were administered i.m. and i.v. (5 µg mRNA) to Ai9 mice and after 48 h cells from different tissues, injected muscle (LM), non-injected muscle (RM), liver (hepatocytes (Hep), hepatic non-parenchymal cells (Hep-NPC)), spleen, kidney, blood and lung were collected and analyzed by flow cytometry. The analysis was conducted using Kaluza version 2.2.1.


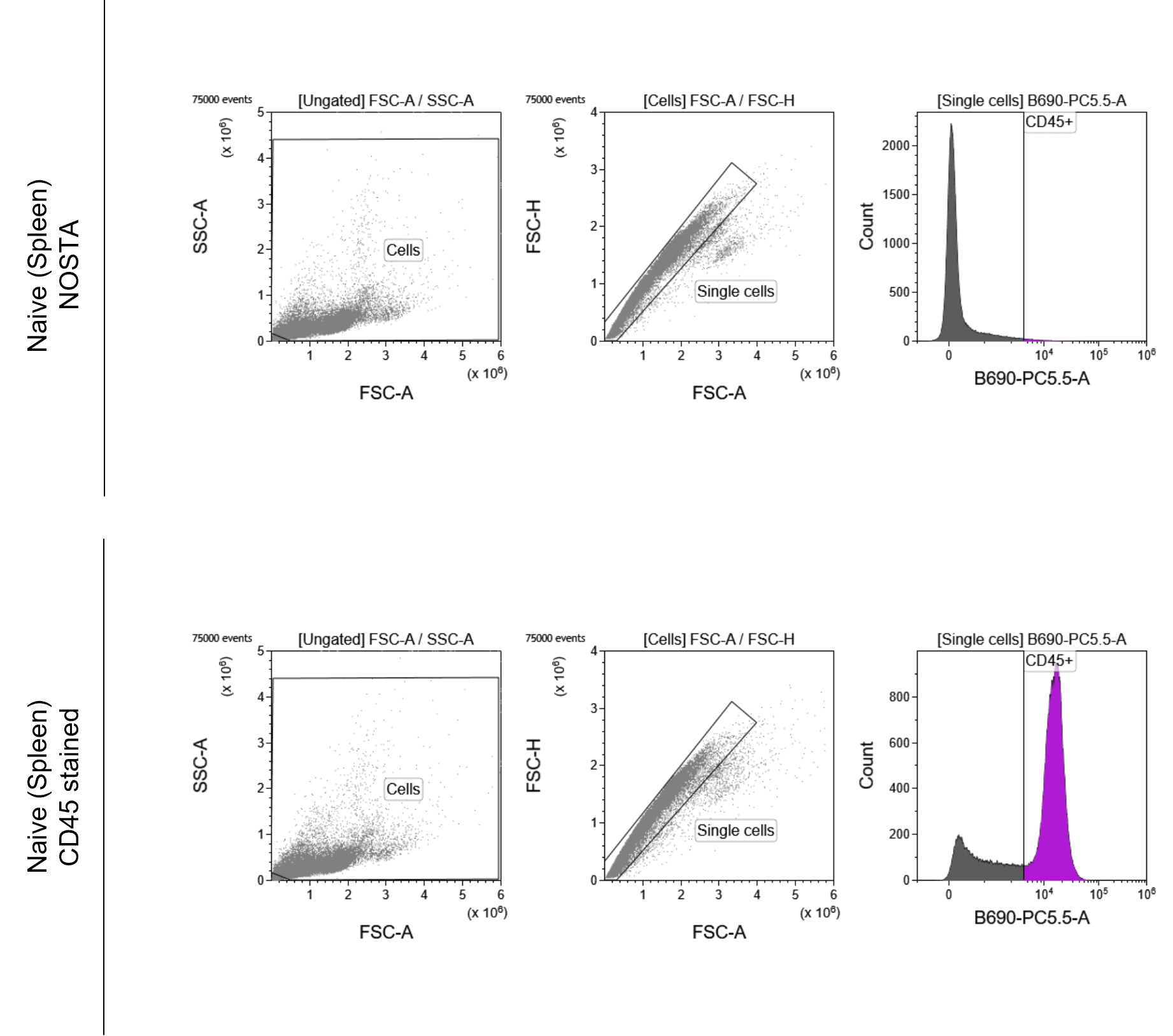


**Figure S32. Gating strategy: Identification of CD45^+^ cells of splenocytes of Ai9/Cre reporter mice.** Single cells were identified by FSC/SSC. PerCP-CD45 antibodies were measured using a 488 nm laser with a 690/50 nm bandpass filter. The analysis was conducted using Kaluza version 2.2.1.


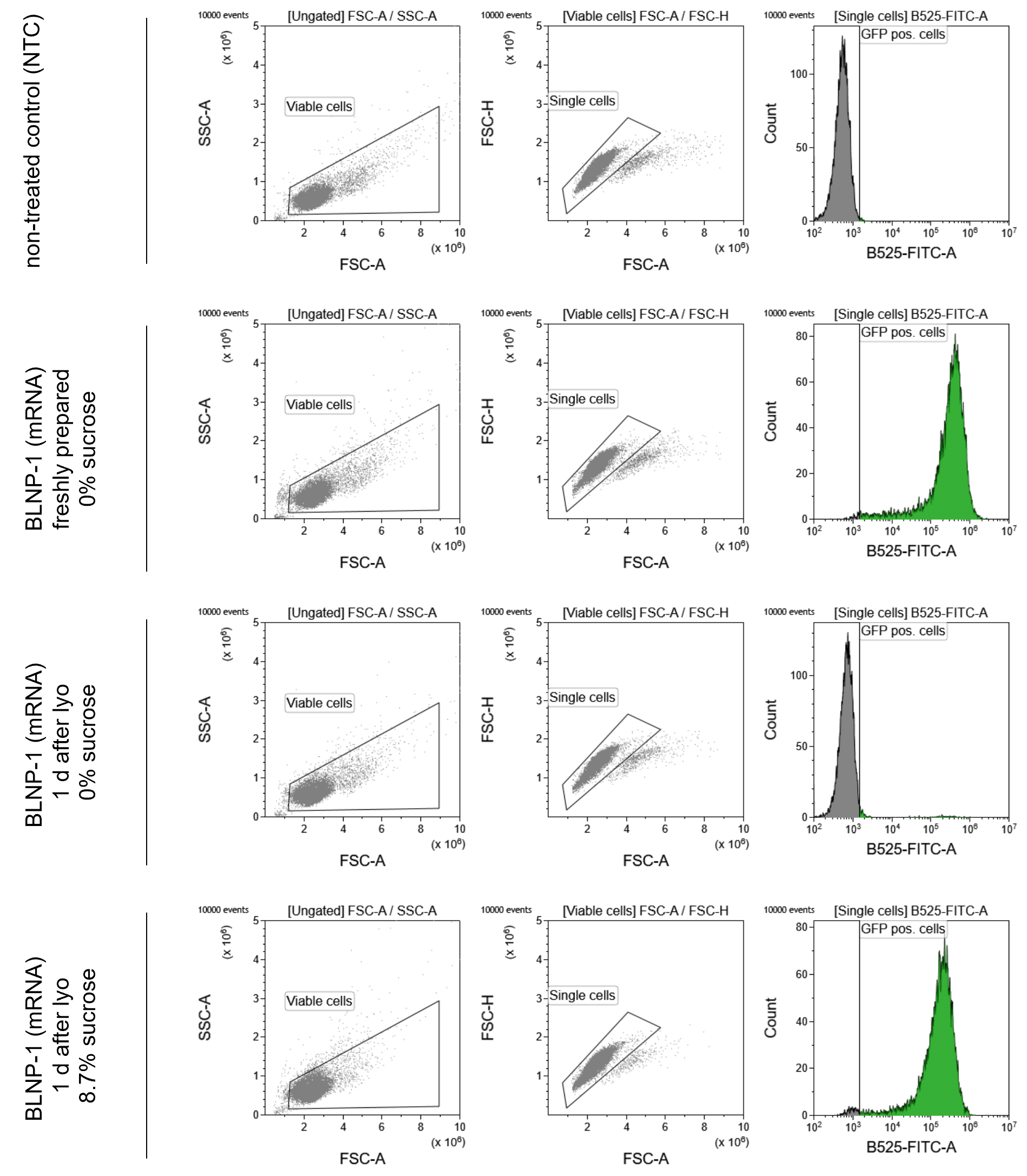


**Figure S33. Gating strategy: transfection of HEK293T cells with pDNA (GFP) and mRNA (GFP) after lyophilization.** Viable single cells were analyzed by forward and sideward scatter (FSC/SSC). Fluorescence was measured at λEx = 488 nm with a 525/40 nm bandpass filter (FITC channel). Positive cells were identified by gating against non-treated cells (NTC). The analysis was conducted using Kaluza version 2.2.1.

## **Supplementary Methods**

**Aqueous lipid formulation by vortex mixing (F1-F4)**

For preparation of lipid formulations by vortex mixing stock solutions of the ionizable lipid SM‑102 (Hölzel, Cat# DCC-DC52025-1g), the phospholipid DSPC (Avanti, Cat# 850365P) and the stealth lipid CHEMS-PMeOx_52_[5] were prepared in 50 mM citrate buffer with a pH value of 4.0 (citrate). The lipids were mixed in citrate to get the indicated molar ratio of lipids. Subsequently, a mixture of mEGFP-N1 pDNA (Addgene, Cat# 54767, isolated with the EndoFree Plasmid Mega Kit (5) (Qiagen Cat#12381) (pDNA (GFP)) in 20 mM HEPES (Capricorn, Cat# HEP-B), supplemented with 5% (v/v) glucose (Sigma-Aldrich, Cat# 49163) (HBG) with a concentration of 60 µg mL^-1^ acid was prepared. Particles were formed by mixing the lipid mixture and pDNA mixture 1 + 1 to receive a pDNA concentration of 30 µg mL^-1^.

**Formulation of BLNP by microfluidics**

Stock solutions of the ionizable lipid SM-102 (Hölzel, Cat# DCC-DC52025-1g), the phospholipid DSPC (Avanti, Cat# 850365P), the stealth lipid DMG-PEG 2000 (DMG-PEG) (Avanti, Cat# 880151P-1g) and the CHEMS-PMeOx_52_[5] were prepared in 20 mM sodium acetate buffer with a pH value of 5.5 (Invitrogen^TM^, Cat# 10679075) diluted with UltraPure water (Invitrogen^TM^, Cat# 10977035). The lipids were equilibrated to room temperature and subsequently homogenized in an ultrasonic bath for 15 min. Afterwards, the lipids were mixed with sodium acetate buffer to reach the defined molar ratio of 82.3/16.3/2.4 mol% (SM-102/DSPC/DMG-PEG or CHEMS-PMeOx_52_). An ultrasonic homogenizer (Carl Roth, Cat# 1T6C.1) operating at 50 W and 100% amplitude was utilized to generate unloaded particles. Following a short incubation period at room temperature, the particles were loaded into a 1 mL glass syringe (Setonic Cat# 2624016) that was integrated into a neMESYS low pressure syringe pumping system (Cetoni, Cat# NEM-B101-02 E). For the genetic material, either tdTomato mRNA (OZ Bioscience, Cat# mRNA2-1000) or tdTomato-N1 pDNA (Addgene, Cat# 54642, isolated with the EndoFree Plasmid Mega Kit (Qiagen Cat# 12381)) was diluted in 20 mM sodium acetate buffer and loaded into a 250 µL glass syringe (Setonic Cat# 2624815) embedded in the syringe pumping system. The particles were formulated to reach a final concentration of genetic material of 60 µg mL^-1^ at a N/P molar ratio of 6 by mixing the genetic material and lipids 1 + 1 in a chip herringbone mixer (Microfluidic ChipShop, Cat# 10000019) at a flow rate of 1000 µL min^-1^. Following a brief incubation period at room temperature, the loaded particles were diluted in a 1 + 1 ratio with PBS (Capricorn Scientific, Cat# PBS-1A). The CETONI Elements software (Version 20220926, Cetoni GmbH, Korbussen, Germany ) was used for the microfluidic system.

**Formulation of control LNP by vortex mixing**

For the preparation of control lipid nanoparticles by vortex mixing, a previously described method was adapted.[4] Stock solutions of the ionizable lipid SM-102 (Hölzel, Cat# DCC-DC52025-1g), the phospholipid DSPC (Avanti, Cat# 850365P), cholesterol (Sigma-Aldrich, Cat# C8667-5g) and the stealth lipid DMG-PEG 2000 (DMG-PEG) (Avanti, Cat# 880151P-1g) were prepared in ethanol. The lipids were mixed for each formulation with a total molarity of 15 mM and a molar fraction of 50/10/38.5/1.5 mol% (SM-102/DSPC/Chol/DMG-PEG). Required amounts of mRNA or pDNA (GFP mRNA and pDNA similar to BLNP formulations) were prepared in 50 mM citrate buffer pH 4 (ThermoFischer, Cat# J61249, diluted with UltraPure DNase/RNase-free distilled water (ThermoFischer, Cat# 10977035)). LNP were formulated by mixing the organic phase and the aqueous phase at a 1:3 volume ratio and a molar N/P ratio of 6 in a 1.5 mL Eppendorf tube using a Vortex 2 (IKA) on max. speed for a total of 15 s. A comparable method was described by Wang *et.al.* and is suitable for LNP preparation.[6] The LNP were slowly diluted with the same volume of PBS buffer pH 7.4 (Alfa Aesar, Cat# J62036, diluted with UltraPure DNase/RNase-free distilled water (ThermoFischer, Cat# 10977035)) as the formulation volume to stabilize the initial formulation.

**Purification of LNP**

After formulation and dilution, the LNP were purified to remove remaining ethanol. The formulations were transferred into Amicon Ultra-4 Centrifugal Filters (30,000 g mol-1 MWCO) and washed by ultrafiltration with PBS buffer 20-fold of the volume of the diluted formulation. They were centrifuged in a swing bucket rotor with 3000 rcf at 4 °C using a 5804 R centrifuge (Eppendorf, Germany) until the retentate volume in the filter was at or below 80 µL. The purified LNPs were collected, and their volume was adjusted to 90 µL.

**Dynamic light scattering and electrophoretic light scattering**

Hydrodynamic diameter (Z-Average), polydispersity index (PDI) and zeta potential were determined by dynamic and electrophoretic light scattering (DLS/ELS) (Zetasizer Ultra, Zetasizer Nano-ZS, Malvern Instruments, Worcestershire, U.K.). For Z-Average and PDI determination nanoparticles prepared as described above were measured in UV cuvettes consisting of polystyrene (Brand GmbH + Co K, Cat# Cat# 7591 50G) in 75 µL buffer (F1-F4, BLNP) or diluted 1 + 9 with PBS (LNP). The instrument was operated at a temperature of 25 °C with a 633 nm He-Ne laser and at a backscattering angle of 173°. Three independent batches were measured to determine hydrodynamic diameters and PDI values. The zeta-potential was characterized using the DTS1070 capillary cuvette (Malvern Panalytical) after 1 + 9 dilution of the nanoparticles (n=1).

**Lyophilization of BLNP**

To evaluate the stability of BLNPs under clinically relevant storage conditions, additional freeze-drying experiments were conducted. BLNP-1 and BLNP-2 were formulated as previously described using GFP mRNA and pDNA. Following formulation, the samples were supplemented with either phosphate-buffered saline (PBS) or a 1:1 mixture with a sucrose (CarlRoth, Cat# 8890.1) solution (17.4% w/v), resulting in a final sucrose concentration of 8.7% (w/v) to act as a cryoprotectant. Prior to freeze-drying, the samples were frozen at –80 °C for 2 h and subsequently lyophilized for 18 h using a Christ Alpha 2-4 LD plus freeze-dryer (Martin Christ, Osterode am Harz, Germany). The lyophilized samples were stored at 4 °C and reconstituted in PBS prior to use. For analysis, dynamic light scattering (DLS) measurements and transfection experiments in HEK293T cells (3.0 µg mL^-1^ nucleic acid) were performed. Prior to the flow cytometry measurement, the supernatant was removed, the cells were detached using trypsin-EDTA (Capricorn, Cat# TRY-1B) and the cells were resuspended in PBS. Flow cytometry data were analyzed using Kaluza software, version 2.2.1, and gating was performed according to the strategy shown in **Fig. S33**.

**Cryo-transmission electron microscopy (cryo‑TEM)**

BLNP were prepared as described above. Cryo-TEM investigations were performed on a FEI Tecnai G^2^ 20 transmission electron microscope at an acceleration voltage of 200 kV. Samples were blotted onto a Quantifoil grid (R 2/2, Quantifoil, 8.5 µL of solutions with a concentration of 2.7 mM) utilizing a Vitrobot Mark IV preparation unit (blotting time 1 s, offset -6 mm). Samples were vitrified utilizing liquid ethane as a cryogen. After blotting, the sample temperature was always maintained at a temperature below –165 °C. The grids were loaded into the cryo-holder (Gatan 626) using the Gatan cryo transfer stage. Images were acquired with a CCD camera (MegaView, Olympus Soft Imaging Solutions, Muenster, Germany). Contrast adjustments and image analysis was performed by ImageJ (Version 1.52a, National Institutes of Health, Bethesda, MD, U. S.).[7]

**Nucleic acid encapsulation efficiency (EE)**

Encapsulation efficiency of LNP formulation was determined using the Quant-iT RiboGreen RNA reagent (Thermo Fisher Scientific Inc., Cat# R11491) adapting the manufacturer’s protocol. Briefly, particle suspensions were incubated with the RiboGreen reagent in the presence and absence of 5 µL mL^-1^ Triton X-100 (Sigma-Aldrich, Cat# X100-100ML) and 100 µg mL^-1^ heparin (Alfa Aesar, Cat# A16198) in Tris-EDTA (TE) buffer (adjusted to pH 7.5 from TE buffer (1X) pH 8.0 (AppliChem GmbH, Cat# A0386,0500)). Fluorescence intensity was measured using an Infinite M200 Pro or Spark plate reader (Tecan Group, Switzerland) at an excitation wavelength of 485 nm and an emission wavelength of 535 nm. The total amount of genetic material bound to RiboGreen dye after release from the particle was compared to the unencapsulated amount bound to RiboGreen dye in the absence of Triton X-100 and heparin and the EE was calculated. All results were obtained from three independent samples, measured in triplicates.

**Gel migration assay for pDNA detection**

For pDNA detection 1% agarose gel was prepared. The agarose (Carl Roth, Cat#HP30.2) solution was supplemented with ethidium bromide (Carl Roth, Cat# 2218.1) (1:10,000). 20 µL of BLNP-1 and BLNP-2 (pDNA, GFP), produced as described above, supplemented with Green Gel Loading Buffer (Jena Bioscience, Cat# PCR-254-gr) were added to the gel to get a final pDNA amount of 250 ng per sample. Free pDNA and PFNs were treated in the same way as references. In addition, 10 µL of ladders (Jena Bioscience, Cat# M-204, Cat# M-214S, Cat# M-212) were added. Electrophoresis was performed for 2 h in TAE buffer using a PowerPac Basic Power Supply (Bio-Rad) at 60 V. Gel images were obtained using Imager CHEMI Premium (VWR, U.S.) with ethidium bromide gel settings (automatic exposure, TLUM - Mid Wave, Filt 605). Images were analyzed with ImageJ Version 1.54j.

**Gel migration assay for mRNA detection**

For mRNA detection 2% agarose gel supplemented with SYBR Green II RNA gel stain (Invitrogen, Cat# #S7564) (1:10,000) was prepared. 20 µL of BLNP-1_CRE_ and BLNP-2_CRE_ or LNP_CRE_, produced as described above, supplemented with RNA Loading Dye (New England Biolabs, Cat# B0363A) were added to the gel to get a final mRNA amount of 250 ng per sample. Free mRNA was treated in the same way as references. In addition, a commercial ladder (New England Biolabs, Cat# N0362S) was added as indicated by the manufacturer. Electrophoresis was performed for 2 h in TBE buffer using a PowerPac Basic Power Supply (Bio-Rad) at 60V. Gel images were obtained using Imager CHEMI Premium (VWR, U.S.) with gel green settings (automatic exposure, TLUM - Mid Wave, Filt 525). Images were analyzed with ImageJ Version 1.54j.

**PrestoBlue assay for determination of cytotoxicity in L-929 cells**

The biological assay is adapted from Solomun *et al.*[7] To determine cytotoxicity, L-929 cells (CLS Eppelheim, Cat# 400260) were seeded in a 96-well plate with a density of 0.1×10^6^ cells mL^-1^ in 100 µL and incubated for 24 h at 37 °C in humidified 5% (v/v) CO_2_ atmosphere in test medium. 1 h prior treatment, the medium was changed to 90 µL fresh test medium. Cells were treated with unloaded BLNP up to a total lipid concentration of 1000 µM. 24 h after treatment, the medium was replaced by a 10% (v/v) solution of PrestoBlue cell viability reagent (Thermo Fisher Scientific, Cat# A13262) diluted with culture medium. The cells were incubated for 45 min. Fluorescence intensity (λEx = 560 nm, λEm = 590 nm) was measured with an Infinite M200 Pro plate reader (Tecan Group, Switzerland). Cells treated with buffer were used as a control, and the viability was calculated relative to the buffer control (NC) after subtracting the blank (PrestoBlue diluted in medium 1:10 without cells). Metabolic activity values were obtained by following equation:

$$rMetabolic acitivity / \%= \frac{{{Sample}_{Mean}-Blank}_{Mean}}{{NC}_{Mean}-{Blank}_{Mean}}\times100$$

**Hemolysis assay for determination of blood compatibility**

The erythrocyte hemolysis assay is adapted from Streiber *et al.*.[8] The interaction of nanoparticles with cellular membranes was detected by measuring the release of hemoglobin from erythrocytes (RBCs) due to potential membrane lysis. The blood from human healthy volunteers provided by the Department of Transfusion Medicine of the University Hospital, Jena, was collected in tubes with citrate. Blood from at least three different donors was used. To isolate RBCs the blood was centrifuged at 4500 × g for 5 min. The pellet was washed three times with PBS. Aliquots of RBCs were mixed with an equal volume of sample solution in PBS to receive the final concentration (test solution).

The test solutions were incubated for 1 h at 37 °C. To determine hemolysis the tubes were centrifuged at 2400 × g for 5 min and the supernatant was transferred to a 96-well plate in three technical replicates. To measure the hemolytic effect of the polymers the absorbance was measured at λ = 544 with λ = 630 nm as reference bandwidth. As a positive control 1% Triton X‑100 (TX) was used (100% hemolysis) and pure PBS was used as negative control (PBS, 0% hemolysis). The hemolysis (%) was calculated as follows:

$$Hemolysis \left( \% \right) = \frac{Absorbance_{Sample}-Absorbance_{PBS}}{Absorbance_{TX}-Absorbance_{PBS}}\times100$$

Values below 2% hemolysis are classified as non-hemolytic, values between 2 to 5% are slightly hemolytic and values above 5% are hemolytic.

For whole blood analysis of BLNP whole blood was used without isolation of erythrocytes and for 10% FCS samples 10% (v/v) PBS were replaced by FCS and added to the isolated erythrocytes prior sample treatment.

**Cell isolation and culture of human primary PBMC and PMNL**

Leukocyte concentrates from freshly withdrawn peripheral blood from male and female healthy volunteers (18 to 75 years, without details about ancestry, race or ethnicity) were provided by the Department of Transfusion Medicine at the University Hospital of Jena, Germany. The experimental procedures were approved by the local ethical committee (approval no. 505001/17) and were performed in accordance with the guidelines and regulations. Written informed consent was obtained from patients. Peripheral blood mononuclear cells (PBMC) and polymorphonuclear leukocytes (PMNL) were separated using dextran (Sigma Aldrich, Cat# 31392; CAS: 9004-54-0) sedimentation of erythrocytes, followed by centrifugation (2000 rpm, 10 min, 20 °C) on lymphocyte separation medium (Histopaque®-1077, Sigma Aldrich, Cat# 10771) to pellet granulocytes, as described previously.[9] Contaminating erythrocytes in the pelleted neutrophils were removed by hypotonic lysis for 40 s using water, and lysis was stopped by adding PBS. PMNL were then washed twice in ice-cold phosphate buffer saline (PBS) and finally resuspended in PBS. The PBMC fraction on top of the lymphocyte separation medium was washed with ice-cold PBS twice. Afterwards, a 1:1 mixture of PBMC and PMNL was seeded in a 24-well plate (0.5×10^6^ cells) in RPMI 1640 supplemented with 1% human serum (tcs Biosciences, Cat# CS100-500-E1), 2 mM L-glutamine, 100 U mL^-1^ penicillin and 100 µg mL^-1^ streptomycin for 1 h for adherence of the cells (37 °C, 5% (v/v) CO_2_).

**Cytokine analysis for investigation of immunomodulatory properties of BLNP**

To assess cytokine release from human immune cells *via* enzyme-linked immunosorbent assay (ELISA), a 1:1 mixture of PBMC and PMNL isolated as described above were seeded in 96-well plates (0.25×10^6^ cells, 225 µL) in RPMI 1640 (Sigma Aldrich, Cat# R8758) supplemented with 5% fetal calf serum, 2 mM L^-1^ glutamine, 100 U mL^-1^ penicillin and 100 μg mL^-1^ streptomycin. Cells were treated with 25 µL BLNP-1, BLNP-2 and LNP to achieve final concentrations of 3.0 µg mL^-1^ mRNA (CRE) for 18 h (37 °C, 5% CO_2_) and levels of cytokines measured by ELISA. Free mRNA (CRE) and only buffer were used as controls. All materials were purchased from R&D systems TNF-alpha: Cat# DY210-05; IL-6: Cat# DY206; IL-1beta/IL-1F2: Cat# DY201-05). Assays were performed according to guidelines from the manufacturer. Standard curves were obtained for each cytokine and used for quantification.

**Assessment of membrane integrity of human monocytes by LDH assay**

Analysis of membrane integrity was performed by LDH assay with human PBMC and PMNL, following an established protocol.[10] The release of LDH was measured using CytoTox96 Non-Radioactive Cytotoxicity Assay (Promega GmbH, Cat# G1780). Briefly, a 1:1 mixture of PBMC and PMNL isolated as described above were seeded in 96‑well plates (0.25×10^6^ cells, 225 µL) in RPMI 1640 (Sigma Aldrich, Cat# R8758) supplemented with 5% fetal calf serum, 2 mM L^-1^ glutamine, 100 U mL^-1^ penicillin and 100 μg mL^-1^ streptomycin. Cells were treated with 25 µL BLNP-1, BLNP-2 and LNP to achieve final concentrations of 3.0 µg mL^-1^ mRNA (CRE). Free mRNA (CRE) and only buffer were used as control. Cells treated with Triton‑X represent 100% LDH release. After 3 h incubation time in humidified 5% (v/v) CO_2_ atmosphere, 50 µL of supernatant from each well was transferred in a 96-well plate with black bottom after centrifugation (250 × g, 4 min, RT). Afterwards, 50 µL of substrate mixture was added and incubated for 30 min at RT in the dark. Finally, 50 µL of stop solution were added and photometric measurement was performed at 490 nm using a Multiskan Spectrum plate reader (Thermo Fisher Scientific).

sgRNA information

Sequence: G*G*U*CUACAAGACCCGCGCCG + modified Scaffold

The sgRNA sequence was obtained with the freeware http://crispor.tefor.net/. As reference sequence for GFP the following sequence was used:

ATGGTGAGCAAGGGCGAGGAGCTGTTCACCGGGGTGGTGCCCATCCTGGTCGAGCTGGACGGCGACGTAAACGGCCACAAGTTCAGCGTGTCCGGCGAGGGCGAGGGCGATGCCACCTACGGCAAGCTGACCCTGAAGTTCATCTGCACCACCGGCAAGCTGCCCGTGCCCTGGCCCACCCTCGTGACCACCCTGACCTACGGCGTGCAGTGCTTCAGCCGCTACCCCGACCACATGAAGCAGCACGACTTCTTCAAGTCCGCCATGCCCGAAGGCTACGTCCAGGAGCGCACCATCTTCTTCAAGGACGACGGCAACTACAAGACCCGCGCCGAGGTGAAGTTCGAGGGCGACACCCTGGTGAACCGCATCGAGCTGAAGGGCATCGACTTCAAGGAGGACGGCAACATCCTGGGGCACAAGCTGGAGTACAACTACAACAGCCACAACGTCTATATCATGGCCGACAAGCAGAAGAACGGCATCAAGGTGAACTTCAAGATCCGCCACAACATCGAGGACGGCAGCGTGCAGCTCGCCGACCACTACCAGCAGAACACCCCCATCGGCGACGGCCCCGTGCTGCTGCCCGACAACCACTACCTGAGCACCCAGTCCGCCCTGAGCAAAGACCCCAACGAGAAGCGCGATCACATGGTCCTGCTGGAGTTCGTGACCGCCGCCGGGATCACTCTCGGCATGGACGAGCTGTACAAGTAA

Selected genome:

Homo sapiens - Human - UCSC Feb. 2009 (GRCh37/hg19) + SNPs: 1000Genomes, ExaC

Protospacer Adjacent Motif (PAM):

20bp-NGG - Sp Cas9, SpCas9-HF1, eSpCas9 1.1

## **Supplementary References**

[1] W. C. Griffin, "Classification of Surface-Active Agents by “HLB”", *J. Cosmet. Sci.* **1949**, *5* (4), 311-326.

[2] W. C. Griffin, "Calculation of HLB Values of Non-Ionic Surfactants", *J. Soc. Cosmet. Chem.* **1954** *1*, 249-56.

[3] Y. Fukuhira, H. Yabu, K. Ijiro, M. Shimomura, "Interfacial tension governs the formation of self-organized honeycomb-patterned polymer films", *Soft Matter* **2009**, *5* (10), 2037-2041.

[4] C. T. Holick, T. Klein, C. Mehnert, et al., "Poly(2-ethyl-2-oxazoline) (POx) as poly(ethylene glycol) (PEG)-lipid substitute for lipid nanoparticle formulations", *Small* **2025**, *21* (16), e2411354.

[5] L. Simon, L. S. Reichel, B. T. Benkhaled, et al., "Polyoxazolines with Cholesterol Lipid Anchor for Fast Intracellular Delivery", *Macromol. Biosci.* **2024**, *24* (12), e2400148.

[6] X. Wang, S. Liu, Y. Sun, et al., "Preparation of selective organ-targeting (SORT) lipid nanoparticles (LNPs) using multiple technical methods for tissue-specific mRNA delivery", *Nat. Protoc.* **2023**, *18* (1), 265-291.

[7] J. I. Solomun, G. Cinar, P. Mapfumo, et al., "Solely aqueous formulation of hydrophobic cationic polymers for efficient gene delivery", *Int. J. Pharm.* **2021**, *593*, 120080.

[8] M. Streiber, N. E. Göppert, V. Bachmann, et al., "Tailoring cationic poly(2-oxazoline)s: Minimizing toxicity and immunogenic response for biological applications", *Mater. Today Chem.* **2025**, *44*, 102549.

[9] K. Günther, C. Ehrhardt, O. Werz, P. M. Jordan, "Protocol for lipid mediator profiling and phenotyping of human M1- and M2-monocyte-derived macrophages during host-pathogen interactions", *STAR Protoc.* **2024**, *5* (3), 103142.

[10] B. Shkodra-Pula, C. Kretzer, P. M. Jordan, et al., "Encapsulation of the dual FLAP/mPEGS-1 inhibitor BRP-187 into acetalated dextran and PLGA nanoparticles improves its cellular bioactivity", *J. Nanobiotechnology* **2020**, *18* (1), 73.
